# Epidemiology of sleep disturbances among medical students in the Middle East and North Africa: a systematic review and meta-analysis

**DOI:** 10.7189/jogh.15.04099

**Published:** 2025-04-25

**Authors:** Sonia Chaabane, Karima Chaabna, Salina Khawaja, Jasmine Aboughanem, Ravinder Mamtani, Sohaila Cheema

**Affiliations:** Institute for Population Health, Weill Cornell Medicine-Qatar, Doha, Qatar

## Abstract

**Background:**

Sleep disturbances and their associated health issues are common among medical students. Despite this, the epidemiology of sleep disturbances among medical students in the Middle East and North Africa (MENA) region remains inadequately understood. Our objective was to synthesise the prevalence of sleep disturbances, including poor sleep quality, insufficient sleep duration, and excessive daytime sleepiness (EDS), and their variation in relation to academic performance and stress levels.

**Methods:**

We performed a systematic review and meta-analysis. Two independent reviewers searched PubMed, Web of Science, Google Scholar, and the reference lists of relevant studies and reviews up to May 2024. We assessed the quality of the included studies using a risk of bias tool. We performed meta-analyses using random-effects models and used Cochran’s Q between-subgroups statistic to test for differences across subgroups. We used the *I^2^* statistic to assess the statistical heterogeneity. Further, we assessed the publication bias using Doi plots.

**Results:**

We included 150 studies conducted in 16 MENA countries. We found that 59.1% of medical students suffer from poor sleep quality (Pittsburgh Sleep Quality Index mean (x̄) = 8.5; 95% confidence interval (CI) = 7.0–10.1), 59.8% have insufficient sleep duration (<7 hours per night) averaging 6.1 hours per night (95% CI = 5.4–6.9), and 38.4% experience EDS (Epworth Sleepiness Scale x̄ = 8.6; 95% CI = 8.0–9.1). Our results indicate a significantly higher prevalence of poor sleep quality among students with moderate or high stress levels during the preclinical training period and in low-income MENA countries. A significantly higher prevalence of insufficient sleep duration was found among students during preclinical academic years. A significantly higher prevalence of EDS was found among students in public medical schools and those in low-income MENA countries. We observed no differences in poor sleep quality and EDS between students with poor and good academic performance.

**Conclusions:**

Our findings highlight the substantial prevalence of sleep disturbances among MENA medical students. Medical schools must address this critical issue with targeted, locally informed, and culturally appropriate interventions. Further research is needed to assess the association between sleep disturbances and identify factors for tailored interventions that mitigate the adverse consequences on medical students’ health and well-being.

**Registration:**

Open Science Framework BF2A6.

Sleep disturbances, including poor sleep quality, insufficient sleep duration, and excessive daytime sleepiness (EDS), are prevalent across countries and age groups and are considered a public health epidemic [1–4] with high economic costs [5–7]. Medical students are particularly vulnerable to sleep disturbances as they experience high levels of stress [8,9] due to excessive workload, tight schedules, contact with severely ill and dying patients, and career-related decision-making stress [10,11]. Furthermore, higher prevalence of anxiety, depression, and sleep disorders [8,12–15] have been reported among medical students compared to non-medical students and the general population [16–18]. These indicators of psychological distress are linked to sleep disturbances [19–26]. Negative effects of disturbed sleep on medical students might include the alteration of neurocognitive function [27,28], elevated unhealthy dietary behaviours [29–31], and weight gain [20,32–34]. Additionally, poor sleep quality and insufficient sleep duration contribute to the development of several chronic diseases and comorbidities, including metabolic syndrome [35,36], respiratory infection [37,38], and cardiovascular diseases [20,39]. Also, EDS is significantly associated with psychological distress [24] and suicidal ideation [26] in medical students.

The Middle East and North Africa (MENA) region includes countries of West and South Asia and North Africa and hosts the highest number of medical schools in their respective continents [40]. In addition to shared lifestyle behaviour and cultural values, MENA countries share similar medical curricula and sociocultural factors [41,42] that are essential in assessing the extent of sleep disturbances and driving the implementation of tailored preventive measures and sleep health policies that, in turn, will improve medical students’ health in the region [7].

A recent call for action highlighted the need for more comprehensive sleep health data in several MENA countries [7]. However, existing systematic reviews on sleep disturbances among medical students either merged various sleep disturbances and sleep disorders [43], discussed sleep disturbance with a limited scope [44–46], did not provide pooled estimates [43], included limited data on MENA countries [43–48], or did not explore the variation in sleep disturbances on students’ stress level [43,44,46,47]. Moreover, several recent individual studies have been published on this topic in MENA countries. Our aim was to synthesise the prevalence of common sleep disturbances, including poor sleep quality, insufficient sleep duration, and EDS, and their variation in relation to academic performance and stress levels among medical students in the MENA region.

## METHODS

We developed the methodology for this systematic review based on the Cochrane Handbook for Systematic Review of Interventions [49] and we followed the AMSTAR-2 checklist and the PRISMA guidelines (Table S1 in the **Online Supplementary Document**) [50]. We developed and registered the research protocol a priori at Open Science Framework.

### Literature search strategy

Two independent reviewers searched PubMed, Web of Science, and the ten first pages on Google Scholar for relevant literature. We developed the database selection and search strategy in consultation with a specialised librarian. The latest search was conducted on 13 May 2024. The search included a combination of controlled vocabulary terms and text words related to sleep disturbances and medical/university students (Box S1 in the **Online Supplementary Document**). Two independent reviewers also manually searched the reference lists of included primary studies, relevant reviews, and the internal literature database we developed, titled ‘Mental Health in University Students.’

### Eligibility criteria

#### Outcomes of interest

Our primary outcome of interest was the prevalence of sleep disturbances among medical students. The specific measures to report were: 1) the average score and prevalence of poor sleep quality, 2) the average mean (x̄) duration of sleep at night and the prevalence of insufficient sleep duration per night (<7 hours) [51], 3) the average score and prevalence of EDS, and 4) the variability in the outcomes of interest according to the population and study characteristics, stress level, and academic performance.

We defined cases of poor sleep quality and EDS as those with abnormal scores according to the scoring system (cut-offs) recommended by the utilised tool. Sleep durations using any measurement tools were eligible to be included in this systematic review. We excluded data on perceived sleep quality or EDS because the individual perception of sleep quality contradicts the actual sleep quality assessed by a tool [52]. We also excluded studies mixing sleep disturbances or sleep disorders.

#### Population of interest

We included medical students enrolled in a medical school located in one of the 20 MENA countries – Algeria, Bahrain, Djibouti, Egypt, Iraq, Jordan, Kuwait, Lebanon, Libya, Morocco, Oman, Pakistan, Palestine, Qatar, Saudi Arabia, Sudan, Syria, Tunisia, the United Arab Emirates, and Yemen. The list of MENA countries used in this study was based on the one developed in a series of published systematic reviews characterising population health in MENA [15,41]. We included studies that included mixed university students only if specific data on medical students (undergoing a medical degree or a Bachelor of Medicine and a Bachelor of Surgery) were available.

### Study design

We included grey literature and any type of observational studies, such as cross-sectional, case-control, or cohort studies. However, we excluded reviews, case reports, letters to the editor, preprints, commentaries, and clinical trials.

### Multi-stage screening and data extraction

We performed duplicate removal, and multi-stage screening using the Rayyan software (Rayyan Systems, Cambridge, Massachusetts, USA). Two independent reviewers conducted the title and abstract screening, full-text screening, and data extraction. Any discrepancies in the previous steps were resolved with a third reviewer to achieve consensus. We excluded studies reported in languages other than English, Arabic, French, and/or Urdu (languages spoken by the authors of this systematic review).

We utilised a standardised extraction template to extract the study, medical student, and outcome characteristics.

### Quality assessment

The methodological quality of the included studies was assessed independently by two reviewers using a validated risk of bias (RoB) tool for prevalence studies [53]. Each included study was assigned a low or high RoB for each assessed item. No summary quality score was computed as per the COSMOS-E guidance [54]. The synthesis of study quality was based on a summary of the low and high risk of bias assessment for each quality domain.

### Synthesis

We conducted meta-analyses to estimate the weighted average (pooled) prevalence of poor sleep quality, insufficient sleep duration, and EDS using the DerSimonian-Laird random-effects model [55]. The random-effects model with the logit transformation of the proportion was used to conduct the meta-analyses pooling prevalence measures and their 95% confidence intervals (CI). We used the Freeman-Tukey double arcsine transformation in the analyses involving the pooling of proportions, using the command ‘sm = PFT’ in *R*, version 4.0.3 (R Core Team, Vienna, Austria) [56]. We computed Clopper-Pearson CIs for individual prevalence measures. If not reported, we calculated the prevalence of poor sleep quality, insufficient sleep duration, and EDS based on raw data reported in the study. We excluded from the meta-analysis studies with insufficient information to compute prevalence data of sleep disturbance outcomes or reporting exclusively median data. We utilised the ‘metaprop’ and ‘metamean’ functions in *R* to conduct the meta-analyses.

We included in the meta-analysis only x̄ scores generated using validated tools and cut-offs for measuring poor sleep quality and EDS. We excluded from the meta-analysis x̄ scores or x̄ sleep duration reported without the corresponding standard deviation (SD). The Pittsburgh Sleep Quality Index (PSQI) and Epworth Sleepiness Scale (ESS) were the most used tools for assessing poor sleep quality and EDS, respectively. Then, random-effects meta-analyses were conducted to estimate the PSQI and ESS x̄ scores, as well as the x̄ sleep duration per night, along with their corresponding 95% CIs.

To explore variability in the prevalence and men score estimates, we conducted subgroup meta-analyses by sex (male, female), country income as per the World Bank country income classification [57], academic training period (preclinical year (first and second year), clinical years (third to sixth year), late clinical years (seventh and eight)), the type of medical school (private, public), stress level (stressed (moderate and high levels), not stressed (low and mild levels)), specific stressors (the COVID-19 pandemic/lockdown, assessment periods), and academic performances (good academic performance group includes students with excellent, very good, good, ≥70% on the percentage scale, 3–5 on a five-point scale, or 3–4 on a four-point scale; poor academic performance includes students with pass, fail, <70% in the percentage scale, <3 on a five-point scale or four-point scale) [58,59].

We assessed the impact of the COVID-19 period on the prevalence of sleep disturbances by classifying data collection time into three main categories: 1) studies conducted between 2020–21 (most likely during the COVID-19 pandemic period), 2) studies conducted before 2020 (most likely before the COVID-19 pandemic period), and 3) studies conducted after 2021 (most likely after the COVID-19 pandemic). If the data collection time was missing, we considered two years before publication as the estimated average time to publication [60].

The recommended sleep duration for health benefits is ≥7 hours per night [61,62]. When a study reported the prevalence of insufficient sleep duration based on different sleep duration cut-offs, we merged and reclassified the cases prior to the inclusion in the meta-analysis into two main groups: sufficient sleep duration (≥7 hours) and insufficient sleep duration (<7 hours). Prevalence measures reported for each academic year were combined and classified into preclinical, clinical, and late clinical according to the medical school curriculum followed by the country where the study was conducted. Cochran’s Q between-subgroups statistic was used to test for differences in prevalence and mean (x̄) score estimates across subgroups [63], with statistical significance considered at a *P*-value ≤0.05.

We used the *I^2^* statistic to assess the heterogeneity between studies [64]. Heterogeneity was considered as substantial when *I^2^*>50% [65]. Univariable and multivariable random-effects meta-regression were used to further explore between-studies heterogeneity [63]. We utilised logit transformation during the meta-regression to estimate odds ratios (OR) and corresponding 95% CIs to measure the magnitude of relative changes in the pooled prevalence of poor sleep quality, insufficient sleep duration, and EDS according to changes in the study-level factors [66]. These later factors with *P* < 0.2 in the univariable regression model were included in the multivariate regression model. Statistical significance was considered at *P* ≤ 0.05 in the multivariate analysis. We conducted meta-regression analyses using ‘metareg’ function in the *R*.

### Publication bias

We assessed the publication bias using the Doi plot, a method that allows a better visual representation of asymmetry compared to the conventional funnel plot and Egger’s test [67,68]. In the Doi plot, the prevalence of each sleep disturbance (effect estimate) is plotted on the x-axis against the normal-quantile (absolute Z-score) for each study on the y-axis [67].

The prevalence of each sleep disturbance was transformed to the log-odds scale for better statistical properties for the meta-analysis [68]. We also estimated the LFK index to detect and quantify the symmetry of study effects in the Doi plot, with an LFK index of zero indicating complete symmetry. The closer the LFK index value is to zero, the more symmetrical the Doi plot is and the lower the risk of publication bias. LFK index values beyond −1 and +1 were deemed consistent with asymmetry and potential publication bias [67]. In cases where we identified publication bias, we estimated the 95% prediction interval to provide the distribution of true outcome measures around the pooled prevalence [69,70].

### Certainty of evidence

We assessed the certainty of the body of evidence using the GRADE approach. As per similar evidence syntheses of observational cohort and cross-sectional studies [65,71], we utilised considerations of the GRADE approach as: 1) RoB or limitations in the detailed design and implementation, 2) unexplained heterogeneity or inconsistency of results of the included studies, 3) precision of the meta-analysis effect estimates, and 4) probability of publication bias [72]. Other GRADE considerations, such as the indirectness of evidence or dose response, were irrelevant to this research question.

## RESULTS

**Figure 1:**
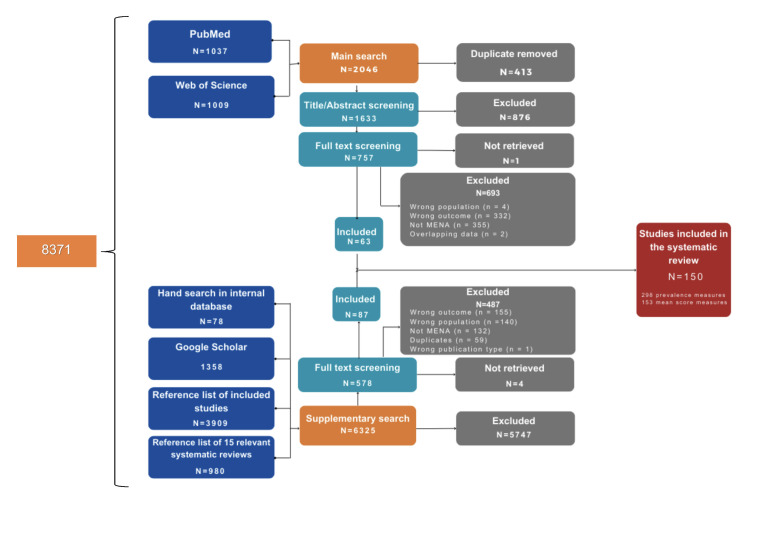
Primary study selection flowchart.

We identified a total of 8371 records through the literature search, and we included 150 primary studies in the systematic review and meta-analysis (**Figure 1**). Most included studies were cross-sectional (n/N = 147/150, 98%), with two cohort studies and one case-control study. The included studies were conducted in Bahrain, Egypt, Iraq, Jordan, Morocco, Lebanon, Oman, Pakistan, Qatar, Kuwait, Saudi Arabia, Sudan, Syria, Tunisia, the United Arab Emirates, and Yemen. They were published between 2003–24 and included a total of 52 149 medical students (Table S2 and S3 in the **Online Supplementary Document**).

### Pooled estimates of poor sleep quality

In the meta-analysis, we included a total of 170 prevalence measures of poor sleep quality retrieved from 72 studies (Table S3 in the **Online Supplementary Document**). The overall pooled prevalence of poor sleep quality in MENA medical students was 59.1% (95% CI = 52.8–65.0) (Figure S1 in the **Online Supplementary Document**). We found statistically significant differences in the prevalence of poor sleep quality between stress levels, academic training periods, and country income levels (**Table 1**; Table S4 in the **Online Supplementary Document**).

**Table 1:** Meta-analysis of sleep disturbances prevalence, mean scores, and mean sleeping time in MENA medical students*

	Sleep quality		Sleep duration		Daytime sleepiness	
	**Poor**	**x̄ score**	**Insufficient (<7 hours)**	**x̄ duration**	**Excessive**	**x̄ score**
**Subgroups**	**Weighted average prevalence, % (95% CI)**	**Weighted PSQI score, x̄ (95% CI)**	**Weighted average prevalence, % (95% CI)**	**Weighted sleep duration in hours, x̄ (95% CI)**	**Weighted average prevalence, % (95% CI)**	**Weighted ESS score, x̄ (95% CI)**
Countries†						
*High-income*	59.2 (49.7–68.0)	8.0 (7.0–9.0)	61.7 (55.2–67.9)	5.7 (4.7–6.7)	41.6 (36.0–47.4)	9.4 (8.2–10.5)
*Upper-middle-income*	42.1 (23.8–62.8)	13.1 (6.6–19.6)	63.1 (51.6–73.3)	NA	31.4 (26.2–37.0)	7.2 (6.7–7.7)
*Lower-middle income*	59.0 (49.7–67.7)	8.3 (4.7–11.8)	58.9 (48.8–68.3)	6.5 (5.4–7.5)	30.7 (20.7–42.9)	8.5 (7.5–9.6)
*Low-income*	73.5 (64.2–81.1)	NA	48.9 (35.3–62.5)	7.0 (6.7–7.3)	59.7 (46.9–71.4)	9.7 (9.3–10.1)
Sex						
*Male*	61.0 (54.6–67.0)	9.1 (7.0–11.3)	56.3 (48.7–63.7)‡	6.3 (4.9–7.7)‡	26.7 (18.1–37.5)	9.7 (7.4–11.9)
*Female*	67.0 (61.1–72.4)	9.5 (6.6–12.4)	NA	7.2 (7.0–7.5)	37.7 (18.6–61.3)	9.2 (4.7–13.6)
Training period§						
*Preclinical*	64.1 (55.7–71.8)	5.9 (4.3–7.6)	60.7 (53.9–67.2)	6.0 (5.1–6.9)	40.3 (22.6–61.0)	7.8 (7.5–8.1)
*Clinical*	59.5 (47.7–70.3)	10.1 (7.0–13.3)	44.3 (31.2–58.2)	5.7 (4.0–7.4)	47.0 (35.4–59.0)	8.9 (7.2–10.5)
*Late clinical*	37.2 (30.9–43.8)	NA	NA	6.2 (6.0–6.3)	38.1 (31.7–44.7)	NA
Data collection time¶						
*Before 2020*	57.4 (50.7–63.8)	8.2 (5.9–10.5)	59.0 (53.9–63.8)	6.6 (6.3–7.0)	37.8 (31.2–44.8)	9.0 (8.2–9.8)
*2020–21*	58.0 (41.2–73.1)	9.5 (8.9–10.1)	64.5 (44.8–80.3)	1.3 (1.2–1.4)	46.9 (33.7–60.5)	NA
*After 2021*	71.3 (47.3–87.3)	11.0 (7.7–14.4)	59.1 (53.3–64.7)	4.8 (1.6–8.0)	38.1 (31.7–44.7)	NA
Type of medical school						
*Public*	59.5 (52.9–65.9)	9.0 (6.9–11.1)	60.1 (54.1–65.8)	6.3 (5.6–7.0)	40.8 (36.2–45.5)	9.2 (8.3–10.1)
*Private*	58.5 (40.3–74.6)	6.9 (5.9–7.9)	53.5 (44.4–62.4)	5.5 (2.7–8.3)	31.6 (27.6–35.9)	7.7 (7.2–8.2)
Characteristics of the study period						
*Regular teaching period*	58.8 (52.2–65.1)	NA	60.5 (55.6–65.2)	NA	38.1 (31.9–44.8)	8.9 (8.1–9.8)
*Assessment period*	NA	NA	NA	NA	44.7 (39.5–50.0)	9.7 (9.3–10.1)
*During COVID-19 with quarantine*	62.2 (46.7–75.6)	NA	15.5 (11.3–20.6)	NA	NA	NA
*During Ramadan*	NA	NA	63.6 (56.9–70.0)	NA	NA	NA
Academic performance‖						
*Good*	65.7 (49.8–78.7)	10.7 (6.4–14.9)	NA	7.1 (6.5–7.6)	40.3 (27.7–34.9)	NA
*Poor*	72.4 (48.5–87.9)	14.4 (11.7–17.1)	NA	7.4 (7.0–7.8)	25.0 (12.7–41.2)	NA
Stress**						
*Stressed*	79.5 (67.2–88.1)	NA	NA	NA	NA	NA
*Not stressed*	53.5 (39.2–67.3)	NA	NA	NA	NA	NA

CI – confidence interval, ESS – Epworth Sleepiness Scale, NA – not available, PSQI – Pittsburgh Sleep Quality Index, x̄ – mean

*Weighted average prevalence measures were obtained using a random-effect model. *P*-value ≤0.05 was considered statistically significant. Pooled prevalence measures for each subgroup include only studies reporting the relevant stratified prevalence measures for the subgroup. Studies with mixed levels of the subgroup or unclear information about the subgroup were excluded from this analysis and are presented in Table S3 and S4 in the **Online Supplementary Document**. The total number of prevalence measures in each outcome may then vary according to the subgroup analysis. The weighted mean sleep quality score is calculated using only studies reporting the mean PSQI score. The weighted mean score is calculated using only studies that reported the mean ESS score.

†MENA low-income countries include Sudan, Syria, and Yemen. Lower-middle-income countries include Algeria, Djibouti, Egypt, Lebanon, Morocco, Pakistan, Palestine, and Tunisia. Upper-middle-income countries include Iraq, Jordan, and Libya. High-income countries include Bahrain, Kuwait, Oman, Qatar, Saudi Arabia, and the United Arab Emirates, according to the World Bank classification [73].

‡Includes studies with a male population only.

§Preclinical year (first and second year), clinical years (third to sixth year).

¶Before 2020 refers to the period likely before the COVID-19 pandemic. 2020–21 is likely during the COVID-19 pandemic and after 2021 is likely the after COVID-19 pandemic.

║The good academic performance group includes students with grades of excellent, very good, good, ≥70% on the percentage scale, 3–5 on a five-point scale, or 3–4 on a four-point scale. Poor academic performance includes pass, fail, <70% in the percentage scale, <3 on a five-point scale or four-point scale.

**The stressed group includes students with moderate and high levels of stress as recommended by the Kessler Psychological Distress Scale (moderate and high levels of stress require specialist referral).

Out of the 53 studies reporting an x̄ score of poor sleep quality, we included 39 studies counting 60 PSQI x̄ score measures of poor sleep quality in the different subgroup meta-analyses (Table S3 and S4 in the **Online Supplementary Document**). We found that the pooled PSQI x̄ = 8.5 (95% CI = 7.0–10.1) (Figure S2 in the **Online Supplementary Document**), which is included in the range score from five to 10, indicating poor sleep quality [74]. Our analysis showed that the pooled x̄ PSQI scores in all subgroups were higher than the cut-off score of ‘5′ indicating a predominance of students with poor sleep quality [61,75] (**Table 1**; Table S5 in the **Online Supplementary Document**). We did not find a statistically significant difference in the x̄ PSQI score between students with good and poor academic performance (Table S5 in the **Online Supplementary Document**). No PSQI x̄ score data according to stress levels was available.

Results of the multivariate meta-regression analyses showed that prevalence measures of poor sleep quality retrieved from studies that included a one sex population only or used measurement tools other than the PSQI were significantly lower compared to studies that included both sex students and used the PSQI tool to measure poor sleep quality. Also, we found that the prevalence measures retrieved from studies conducted after 2021 are significantly higher compared to those from studies conducted before 2020. The study characteristics listed above are likely to be significant contributors to the observed between-study high heterogeneity (**Table 2**).

**Table 2:** Meta-regression models for sleep disturbance prevalence, mean scores, and mean sleeping time in MENA medical students*

	Studies	Univariable regression model		Multivariable regression model	
	**n**	**OR (95% CI)**	***P*-value**	**OR (95% CI)**	***P*-value**
**Poor sleep quality**					
Gender					
*Mixed male/female population*	67	ref	ref	ref	ref
*One sex population†*	4	0.75 (0.26–2.19)	0.60	1.44 (0.56–3.72)	0.45
*Not reported*	1	0.09 (0.01–0.77)	0.03	0.09 (0.02–0.53)	0.007‡
Sampling method					
*Probability*	28	ref	ref		
*Non-probability/unclear*	44	0.85 (0.51–1.43)	0.54		
Sample size (n)					
*>100*	69	ref	ref		
*≤100*	3	0.80 (0.22–2.89)	0.74		
Measurement tools					
*PSQI*	62	ref	ref	ref	ref
*Other validated tools*	4	0.30 (0.11–0.82)	0.02	0.18 (0.07–0.48)	0.0006‡
*Non-validated/unclear*	6	0.21 (0.09–0.49)	0.0002	0.16 (0.07–0.34)	<0.0001‡
The response rate in %					
*≥75*	23	ref	ref		
*<75 or unclear*	49	1.15 (0.67–1.97)	0.62		
Data collection time§					
*Before 2020*	55	ref	ref	ref	ref
*2020–21*	9	1.03 (0.48–2.19)	0.95	1.96 (0.99–3.88)	0.054
*After 2021*	8	1.83 (0.82–4.09)	0.14	2.34 (1.19–4.57)	0.013‡
Study period					
*During regular teaching period*	67	ref	ref		
*During COVID-19 period with quarantine*	5	1.16 (0.43–3.12)	0.78		
Academic period¶					
*Pre-clinical*	6	ref	ref		
*Clinical*	9	0.74 (0.24–2.27)	0.60		
*Late clinical*	1	0.27 (0.03–2.72)	0.27		
*Mixed/unknown*	56	0.64 (0.26–1.61)	0.35		
University type					
*Public*	58	ref	ref		
*Private*	9	0.96 (0.44–2.07)	0.91		
*Mixed private and public/unknown*	5	0.82 (0.30–2.23)	0.69		
Countries classification					
*High-income*	34	ref	ref		
*Low-income*	3	1.93 (0.54–6.93)	0.31		
*Lower-middle-income*	32	0.99 (0.59–1.67)	0.97		
*Upper-middle-income*	3	0.50 (0.14–1.78)	0.29		
**Insufficient sleep duration**					
Gender					
*Mixed male/female population*	67	ref	ref		
*One gender population†*	6	0.86 (0.41–1.83)	0.70		
Sampling method					
*Probability*	27	ref	ref		
*Non-probability/unclear*	46	1.18 (0.77–1.81)	0.44		
Sample size (n)					
*>100*	65	ref	ref		
*≤100*	8	1.17 (0.60–2.30)	0.60		
Measurement tools					
*PSQI*	21	ref	ref	ref	ref
*ESS*	1	0.49 (0.08–2.81)	0.42	1.39 (0.22–8.88)	0.73
*GHQ-12*	1	1.25 (0.22–7.00)	0.80	1.11 (0.23–5.40)	0.89
*SLEEP-50*	1	0.74 (0.13–4.17)	0.74	0.68 (0.13–3.71)	0.66
*QoL*	1	1.22 (0.22–6.86)	0.82	2.68 (0.45–16.06)	0.28
*BCSQ-12*	1	0.29 (0.05–1.64)	0.16	0.33 (0.07–1.67)	0.18
*YIAT*	1	0.28 (0.05–1.55)	0.14	0.25 (0.05–1.19)	0.08
*Self-developed or adapted questionnaire/unclear*	51	1.10 (0.71–1.72)	0.67	1.17 (0.77–1.80)	0.46
Response rate in %					
*≥75*	22	ref	ref		
*<75% or unclear*	45	0.87 (0.56–1.37)	0.55		
Data collection time§					
*Before 2020*	59	ref	ref		
*2020–21*	11	1.26 (0.71–2.40)	0.44		
*After 2021*	3	1.00 (0.36–2.81)	1.00		
Study period					
*During the regular teaching period*	72	ref	ref	ref	ref
*During COVID-19 period with quarantine*	1	0.12 (0.02–0.66)	0.015	0.09 (0.02–0.47)	0.004‡
Academic period¶					
*Pre-clinical*	8	ref	ref		ref
*Clinical*	5	0.43 (0.16–1.14)	0.09	0.40 (0.14–1.19)	0.10
*Mixed/unknown*	60	0.82 (0.43–1.56)	0.54	0.85 (0.46–1.55)	0.59
University type					
*Public*	56	ref	ref		ref
*Private*	12	0.78 (0.45–1.35)	0.38	0.78 (0.44–1.38)	0.40
*Mixed private and public/unknown*	5	2.00 (0.90–4.44)	0.09	1.74 (0.83–3.64)	0.14
Countries classification					
*High-income*	35	ref	ref		ref
*Low-income*	6	0.59 (0.28–1.28)	0.18	0.77 (0.36–1.64)	0.50
*Lower-middle-income*	26	0.89 (0.57–1.39)	0.60	1.18 (0.73–1.88)	0.50
*Upper-middle-income*	6	1.06 (0.50–2.28)	0.88	1.14 (0.55–2.36)	0.72
**Excessive sleep duration**					
Gender					
*Mixed male/female population*	29	ref	ref		
*One gender population†*	3	0.60 (0.24–1.49)	0.27		
Sampling method					
*Probability*	16	ref	ref		
*Non-probability/unclear*	16	0.73 (0.43–1.23)	0.23		
Sample size (n)					
*>100*	32	ref	ref		
*≤100*	0	NA	NA		
Measurement tools					
*Validated*	31	ref	ref		
*Non-validated*	1	0.98 (0.21–4.52)	0.98		
Response rate in %					
≥75	9	ref	ref		
<75 or unclear	23	0.82 (0.46–1.47)	0.50		
Data collection time§					
*Before 2020*	29	ref	ref		
*2020–21*	2	1.46 (0.50–4.28)	0.49		
*After 2021*	1	1.01 (0.22–4.58)	0.99		
Study period					
*During the regular teaching period*	31	ref	ref		
*During assessment period*	1	1.31 (0.29–5.93)	0.73		
Academic period¶					
*Pre-clinical*	3	ref	ref		
*Clinical*	4	1.33 (0.43–4.10)	0.62		
*Late clinical*	1	0.91 (0.17–4.96)	0.91		
*Mixed/unknown*	24	0.86 (0.35–2.12)	0.74		
University type					
*Public*	27	ref	ref	ref	ref
*Private*	2	0.66 (0.23–1.90)	0.45	0.92 (0.37–2.32)	0.86
*Mixed private and public/unknown*	3	0.46 (0.19–1.12)	0.09	0.34 (0.16–0.75)	0.01‡
Countries classification					
*High-income*	15	ref	ref	ref	ref
*Low-income*	3	2.12 (0.92–4.90)	0.08	2.82 (1.27–6.24)	0.01‡
*Lower-middle-income*	13	0.64 (0.39–1.06)	0.08	0.65 (0.40–1.05)	0.08
*Upper-middle-income*	1	0.65 (0.17–2.50)	0.53	0.60 (0.17–2.07)	0.42

BCSQ-12-SS – burnout, clinical subtypes questionnaire, CI – confidence interval, ESS – Epworth Sleepiness Scale, GHQ-12 – general health questionnaire, GSS – general sleep scale, NA – not applicable, PSQI – Pittsburgh Sleep Quality Index, QoL – quality of life, ref – reference, SQS – sleep quality scale, YIAT – Young’s Internet Addiction Test

*Significant variables with *P* < 0.2 are included in the multivariate regression model. Associations between study-level factors and the prevalence of the sleep disturbances are reported as odds ratios, with their corresponding 95% confidence intervals.

†The study population included only male studies.

‡Significant results (*P* ≤ 0.05).

§Before 2020 refers to the period likely before the COVID-19 pandemic, 2020–21 likely during the COVID-19 pandemic, and after 2021 likely the after COVID-19 pandemic.

¶Preclinical year (first and second year), clinical years (third to sixth year).

‖MENA low-income countries include Sudan, Syria, and Yemen. Lower-middle-income countries include Algeria, Djibouti, Egypt, Lebanon, Morocco, Pakistan, Palestine, and Tunisia. Upper-middle-income countries include Iraq, Jordan, and Libya. High-income countries include Bahrain, Kuwait, Oman, Qatar, Saudi Arabia, and the United Arab Emirates, according to the World Bank classification [73].

### Pooled estimates of insufficient sleep duration

We retrieved a total of 73 prevalence measures of insufficient sleep duration (<7 hours per night) from 73 studies and included them in the meta-analysis (Table S3 in the **Online Supplementary Document**). We found that the overall pooled prevalence of insufficient sleep duration at night among the MENA medical students is 59.8% (95% CI = 54.7–64.6) (Figure S3 in the **Online Supplementary Document**). We found statistically significant differences in the prevalence of insufficient sleep duration between students studying in preclinical and clinical training years and according to the characteristics of the study period (**Table 1**; Table S4 in the **Online Supplementary Document**).

While 20 studies reported the x̄ sleep duration at night (Table S3 in the **Online Supplementary Document**), our meta-analyses included 17 studies, encompassing 31 x̄ sleep duration times at night (Table S3–5 in the **Online Supplementary Document**). Overall, we found that the pooled sleep duration at night is x̄ = 6.1 (95% CI = 5.4–6.9) (Figure S4 in the **Online Supplementary Document**), which is below the recommended seven to nine hours of sleep per night [51]. The pooled x̄ sleep duration at night remained below the recommended sleep duration (x̄ = 6.4; 95% CI = 5.8–67.0) following the exclusion of data from the study by Meo (2022), reporting the extremely low sleep duration of 1.3 hours per night (Table S2 in the **Online Supplementary Document**). The pooled x̄ sleep duration at night was below the recommended duration in all subgroups except for female students and students in low-income countries. We also found significant differences in sleep durations at night according to sex, data collection time, and country income. No statistically significant differences in the x̄ sleep duration were found between students with good and poor academic performance or between those who were stressed and not stressed (Table S5 in the **Online Supplementary Document**).

The multivariate meta-regression analyses showed that the prevalence measure retrieved from a study conducted during the COVID-19 pandemic with quarantine was significantly lower than that conducted during the regular teaching period (**Table 2**). This characteristic of the study period likely significantly contributes to the observed between-study heterogeneity (Table S4 in the **Online Supplementary Document**).

### Pooled estimates of EDS

Out of 33 studies with data on EDS, we included 32 in our meta-analyses, including 55 prevalence measures of EDS (Table S3 in the **Online Supplementary Document**). Overall, the pooled prevalence of EDS in MENA medical students was 38.4% (95% CI = 32.3–44.8) (Figure S5 in the **Online Supplementary Document**). We found statistically significant differences in the EDS prevalence according to the country income and university type (public *vs.* private) (**Table 1**; Table S4 in the **Online Supplementary Document**). No statistically significant differences in the EDS prevalence were found between students with good and poor academic performance (Table S4 in the **Online Supplementary Document**). No data on the prevalence or mean score of EDS according to stress levels were available.

We included only 14 studies reporting x̄ scores of EDS using the ESS tool in the meta-analyses (Table S3 in the **Online Supplementary Document**). The pooled x̄ ESS score was 8.6 (95% CI = 8.0–9.1) (Figure S6 in the **Online Supplementary Document**), which is below the cut-off score of 10, indicating normal levels of daytime sleepiness [53,54,76]. The pooled x̄ ESS scores indicate normal levels of daytime sleepiness across all subgroups (**Tables 1**; Table S5 in the **Online Supplementary Document**).

The multivariate meta-regression analyses showed that prevalence measures retrieved from studies that included public and private medical school students were significantly lower than those from those that included public medical school students only (**Table 2**). We found that prevalence measures retrieved from studies conducted in low-income MENA countries were significantly higher compared to those from studies conducted in high-income MENA countries. These study characteristics are likely to be significant contributors to the observed between-study high heterogeneity (Table S4 in the **Online Supplementary Document**).

### The risk of biases in the included studies

**Figure 2:**
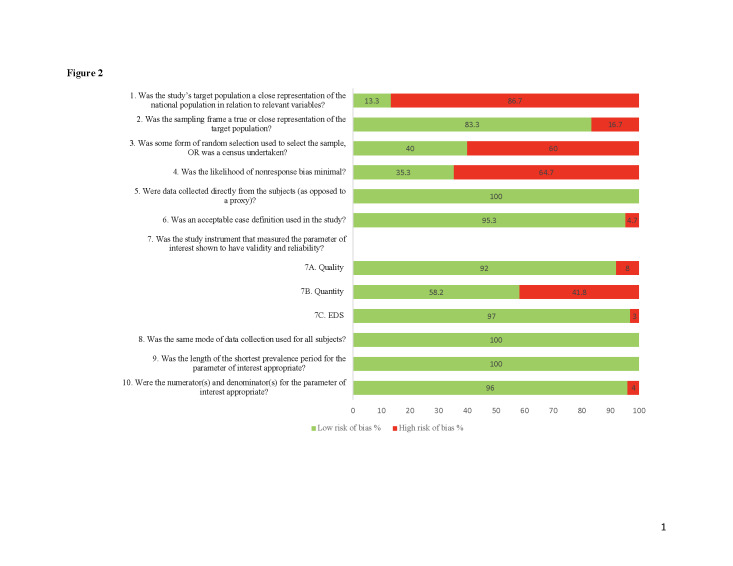
Summary of the quality assessment of the included primary studies. The assessment of the methodological quality included studies was performed using the risk of bias tool for prevalence studies [77]. 7A includes only studies with poor sleep duration data (n = 87), 7B includes only studies with insufficient sleep duration (n = 91), and 7C includes only studies with excessive daytime sleepiness as data (n = 33).

Regarding the external validity, 86.7% of the studies (n/N = 130/150) had a high RoB related to the representativeness of the national and target populations. Additionally, only 40% (n/N = 60/150) of the included studies used a random sampling method, and 64.7% (n/N = 97/150) had a high likelihood of nonresponse bias. However, the sampling frame of most of the included studies (n/N = 125/150, 83.3%) was a true or close representation of the target population (**Figure 2**; Table S6 in the **Online Supplementary Document**).

A low RoB is assigned to the internal validity as all included studies used the same mode of data collection for all subjects, collected data directly from the targeted population, used an appropriate length of the prevalence period for sleep disturbances, and included appropriate numerators and denominators for the studied sleep disturbances. However, most of the included studies used an acceptable case definition of the sleep disturbance of interest (n/N = 143/150, 95.3%) and employed a validated instrument to measure each sleep disturbance, which reinforces the internal validity of our results (**Figure 2**; Table S6 in the **Online Supplementary Document**).

### The probability of publication bias

**Figure 3:**
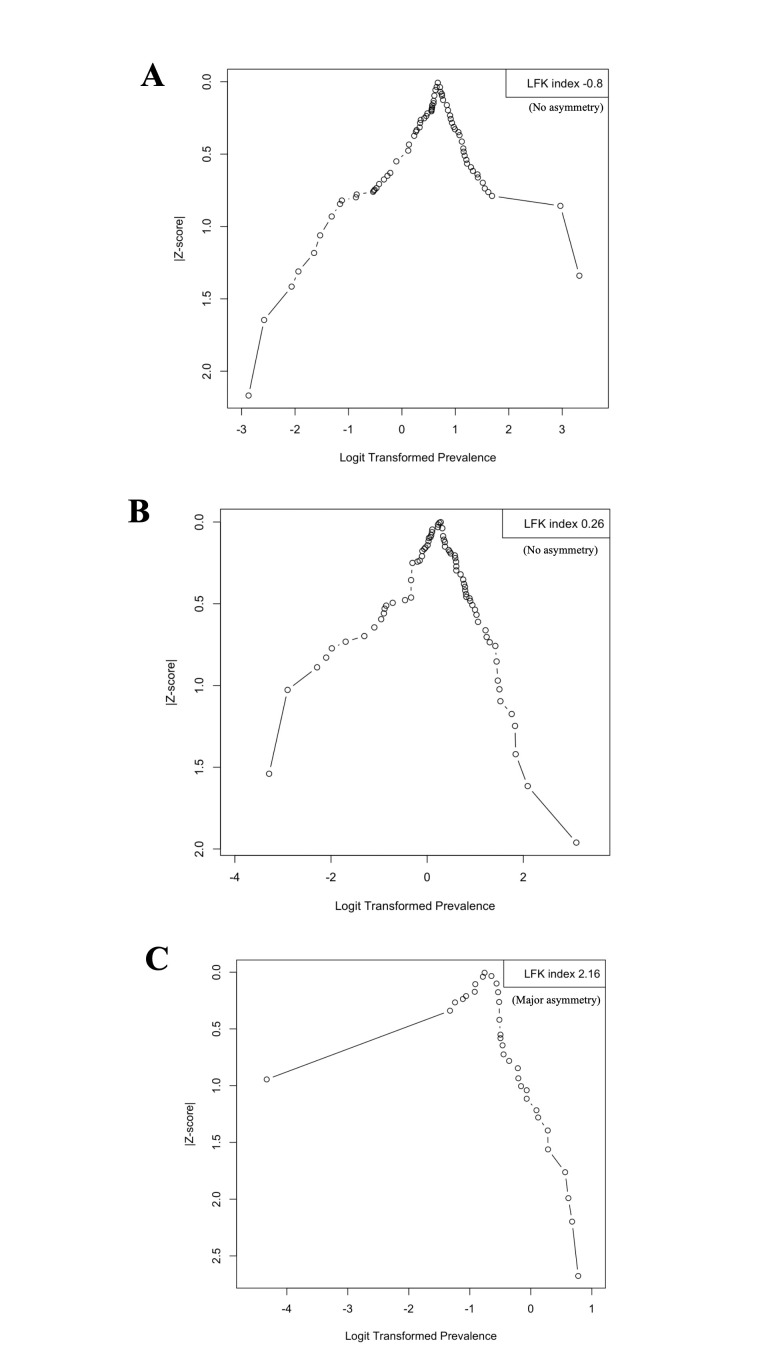
Publication bias assessed via the Doi plot using the prevalence of sleep disturbances as an effect estimate and the Luis Furuya-Kanamori index. The prevalence of each sleep disturbance (effect estimate) is plotted on the x-axis against the normal-quantile (absolute Z-score) for each study on the y-axis. The dots on the Doi plot represent individual prevalence measures reported by each included study. **Panel A.** Poor sleep quality. **Panel B** Insufficient sleep duration. **Panel C.** Excessive daytime sleepiness.

The visual inspection of the Doi plots and LFK index is consistent, with no evidence of publication bias for poor sleep quality (LFK = –0.8) and insufficient sleep duration (LFK = 0.26) (**Figure 3**, Panel A and B). However, the visual inspection of the Doi plot and LFK index indicates a positive asymmetry and evidence of publication bias for EDS (LFK = 2.16), suggesting that studies with higher prevalence are more frequently published (**Figure 3**, Panel C). Additionally, we found that the 95% prediction interval for the pooled prevalence of EDS is 11.6–74.8% (data not shown).

### Certainty of the evidence

Overall, despite potential constraints in external validity for the three sleep disturbance outcomes and the possibility of publication bias for EDS data, the pooled prevalence estimates for these outcomes demonstrate robustness attributed to the strong internal validity of the included studies. However, we found that the between-studies heterogeneity likely impacts the precision of the pooled prevalence estimates. Consequently, we rated the certainty of the available evidence as moderate.

## DISCUSSION

In our systematic review, we included 150 studies conducted across 16 of the 20 MENA countries. We found that sleep disturbances, particularly poor sleep quality and insufficient sleep duration at night, are substantially prevalent among medical students. Most students exhibited normal levels of daytime sleepiness. However, the pooled sleep duration at night was below the recommended sleep time of seven hours per night. Overall, high-risk groups for these sleep disturbances included students in public medical schools, those studying in preclinical years those with moderate or high-stress levels, and those in low-income MENA countries.

### Poor sleep quality

Our MENA pooled prevalence of poor sleep quality was 59.1%, with subgroups ranging from 42.1–73.5%, which was comparable to the global estimates in recent meta-analyses, ranging between 39.8–72.9% [44,46–48,55,56]. The lower poor sleep quality prevalence was reported among medical students in China (25.00–27.38%) [78] could be explained by the higher cutoff scores (seven or eight) used in the Chinese PSQI version, which may underestimate the prevalence compared to studies using a PSQI cutoff of ‘5′ as those included in our systematic review. Comparing medical students with other university students is challenging because most of the available literature on sleep disturbances combines both medical and non-medical university student groups [57–59]. Future collection of data focusing on each type of university students can help identify high risk students and related risk factors. The pooled mean PSQI score of 8.5 estimated in our meta-analysis is higher than the x̄ score estimated among medical students globally, ranging between 5.1–6.3, reinforcing the predominance of MENA medical students with poor sleep quality [46–48].

We found a higher prevalence of poor sleep quality among medical students in low-income MENA countries, which could be attributed to local environment and conditions [47], higher life stressors, and the lifestyle [63]. The important differences between countries in the extent of poor sleep quality among both medical and university students [44,46,47,56,57,63], could be explained by cultural differences across countries [47,60] and/or the country’s income level, as found in our study and others [62].

While existing literature emphasises a worsening of sleep problems during the clinical training years among medical students [15,60,62,64], mainly explained by the involvement in patient care and night shifts during clinical rotations [44,66], we found a significantly higher prevalence of poor sleep quality during the preclinical training period. This could be explained by the transition to university life, managing a high academic load as compared to high school, and a competitive environment, all of which contribute to elevated stress levels and sleep disturbances during these initial years [60,67–69]. Our results open new avenues for understanding and addressing sleep health issues among medical students, particularly during the preclinical education phase.

### Insufficient sleep duration

Our pooled prevalence of insufficient sleep duration at night of 59.8% in the MENA medical students was comparable to the global prevalence estimates from the previous meta-analysis (58.7%) [46] and higher than the prevalence estimates among Chinese university students (43.9%) [70]. Our data indicate that MENA medical students sleep 6.1 hours per night, which is similar to the global average for medical students (6.3–6.45 hours per night) [44,46–48], but lower than the seven hours per night reported among Chinese university students [70], suggesting that medical students could be more affected by insufficient sleep duration than other university students [72]. Caution is needed when interpreting the significantly higher prevalence of insufficient sleep duration found among students in their preclinical academic years and during Ramadan due to limited data. Inconsistently with other studies [44,62], our data do not indicate a difference in the prevalence of insufficient sleep duration between countries with different income levels.

### EDS

The pooled x̄ ESS score of 8.6 estimated in our meta-analysis is similar to x̄ ESS score estimated among medical students globally (8.9) [48] suggesting a predominance of students with normal daytime sleepiness (ESS score <10) [54]. While our pooled prevalence of EDS (38.4%) is comparable to the global prevalence in medical students (31.0–35.9%) [43–45,48], it represents a non-negligible proportion of medical students affected by EDS. A similar prevalence of EDS was found in Indian university students (17–44%) [55] suggests that EDS might be similarly distributed among medical and non-medical university students.

Our findings align with significant differences in EDS prevalence among medical students across countries [43–45,47,48]. The significantly higher EDS prevalence found among students in public medical schools should be interpreted with caution, given the limited number of prevalence measures from private schools (n = 2) compared to public schools (n = 27).

### Sleep disturbances and sex

The difference in sleep disturbances between sexes remains unclear. Our study, along with others [56,59,62,78–83], found no differences in poor sleep quality or EDS between male and female students. However, other studies reported that female students experience a higher prevalence of sleep problems compared to male students [55,84].

### Sleep disturbances and COVID-19

We did not find differences in poor sleep quality, insufficient sleep duration, or EDS among MENA medical students after the COVID-19 pandemic, which is consistent with previous findings in medical students globally [44].

### Sleep disturbances and stress

Our data suggest a potential association between poor sleep quality and moderate to high-stress levels, which aligns with previous findings in university students [85–88]. This suggests that lifestyle interventions aimed at reducing stress – such as, physical activity, relaxation or mindfulness techniques – could help improve sleep quality [89]. However, caution should be exercised when interpreting the non-significant difference in sleep duration at night between stressed and non-stressed medical students due to limited data. Further large-scale studies should investigate the association between insufficient sleep duration and stress levels in medical students.

### Sleep disturbances and academic performance

The present meta-analysis did not find significant differences in poor sleep quality and EDS between poor and good academic performance levels. However, previous data showed that both poor sleep quality [43,48,60,90–93] and EDS [48,91,94] were associated with poor academic performance in medical students. This inconsistency could be explained by the limited data included in our meta-analysis. The difference in insufficient sleep duration according to academic performance could not be investigated, given the missing data. The literature remains inconsistent on the association between insufficient sleep duration and poor academic performance in medical students [48,92,95]. This aspect needs to be investigated in future studies.

Although complex and multifactorial, the relationship between sleep duration and sleep quality suggests there is a developmental path to EDS among medical students [45]. Addressing both sufficient sleep at night and good sleep quality is then likely to prevent EDS. Recent meta-analyses indicate that cognitive-behavioural therapy-based interventions (*e.g.* psychoeducation, sleep schedule) have substantial effects on sleep duration and other sleep parameters in college students [96–98]. In contrast, sleep hygiene-based interventions (*e.g.* sleep hygiene handout) and other psychotherapeutic interventions (*e.g.* Gestalt therapy or imagery rehearsal therapy) have an overall medium effect on sleep duration and sleep onset latency [96,99]. Also, available evidence indicates that sleep extension interventions increase the sleep duration by 45 minutes, on average, with direct sleep extension interventions (*e.g.* regularisation of sleep-wake schedules, bedtime extension) being more effective as compared to indirect interventions (*e.g.* behavioural-educational sleep intervention, sleep health promotion program) in college age population [97,100]. Some artificial intelligence platforms may offer a valuable resource to guide interventions and improve sleep outcomes for university students [101]. Additionally, medical schools can support students in managing disturbed sleep and its consequences by implementing sleep-promoting organisational policies (such as housing, noise reduction, and hours of study) and limiting on-campus access to sleep-inhibiting foods and beverages) [45,102].

### Strengths and limitations

To our knowledge, this is the first systematic review to focus on sleep disturbances commonly found among students in the MENA region. Most of the included studies were of good methodological quality and used validated tools to assess sleep disturbances, which strengthens the validity of our findings. Other strengths are the large number of included studies from most countries of the MENA region, expanding the generalisability of our findings.

We acknowledge that limitations lie in the between-study heterogeneity, which is inevitable in meta-analyses of epidemiological studies [46,103]. This heterogeneity may arise from factors such as mixed-gender populations, characteristics of data collection time and study period, the type of medical school, and the measurement tools used. Nevertheless, we performed several subgroup analyses and meta-regressions to explore potential sources of heterogeneity.

While most sleep disturbances were assessed using validated tools, the type of validated tool used had a variable impact on estimates, suggesting a contribution of the assessment tool to the observed heterogeneity along with mixed groups or unknown subgroup information.

Additionally, in our meta-regression analyses, we revealed a statistically significant contribution of data collection time, study period, and country income to the observed heterogeneity. However, analyses of other study characteristics, such as sample size, stressors during the study period, and stage of the training period, yielded non-significant results, likely due to the small number of included studies in these subcategories (<10 effect measures) [104]. However, the interpretation of the results based strictly on the statistical significance should be avoided, and the impact of these factors needs to be confirmed as student sleep needs and management during these periods might differ.

Although the observed heterogeneity brings uncertainty pertaining to the precision of the prevalence estimates, the large number of prevalence measures, mostly assessed using validated tools, provides adequate evidence that sleep disturbances are substantially prevalent among MENA medical students.

Although all included studies assessed sleep disturbances using self-reported screening questionnaires – which could be subject to recall bias – it has been demonstrated that objective sleep quality measurements are reflected in subjective sleep ratings [105]. Most studies included in this systematic review were constrained by non-response bias or limited generalisability. Our findings may not be generalisable to upper-middle income and low-income MENA countries, given the limited number of studies performed in these countries. Although we detected a publication bias related to the available EDS data, we are confident that the true prevalence of EDS is within the prediction interval. Despite these limitations and the presence of heterogeneity, the interpretation of the computed sleep disturbance estimates is not affected.

As most of the included studies were cross-sectional, the temporal sequencing of sleep disturbances and associated factors cannot be established, limiting conclusions on potential causal associations. However, this synthesis provides valuable insights that can generate hypotheses and guide the design of future research.

Several other factors are potentially related to sleep disturbances, such as socioeconomic factors, comorbidities, gender norms, access to mental health services, lifestyle factors, sleep timing, and the presence of sleeping disorders (*e.g.* insomnia) [47,60,72,106] and were not explored due to insufficient data. Further strengthening the evidence base for academic and non-academic factors disturbing sleep during medical training, using a longitudinal study design examining changes in sleep health before and during medical education has utility in the early detection and prevention of sleep disturbances and their consequences.

## CONCLUSIONS

We revealed that medical students frequently experience poor sleep quality, insufficient sleep duration at night, and EDS. Identified high-risk groups for these sleep disturbances include students in public medical schools, in preclinical years, with moderate or high-stress levels, and in low-income MENA countries. We found no differences in these sleep disturbances between students with poor and good academic performance levels. Given the complexity of sleep health, clear, accessible, and up-to-date sleep guides adapted to academic and local cultural settings could help prevent adverse consequences on students’ health and well-being.

## Additional material


Online Supplementary Document
