# Supplementary material for: Epidemiology of sleep disturbances among medical students in the Middle East and North Africa: a systematic review and meta-analysis
Source: J Glob Health. 2025 Apr 25;15:04099. doi: 10.7189/jogh.15.04099 (PMC12023807; doi:10.7189/jogh.15.04099)
Supplement: Online Supplementary Document [file jogh-15-04099-s001.pdf]

Supplement to: Chaabane S, Chaabna K, Khawaja S, Aboughanem J, Mamtani R, Cheema S. Epidemiology of sleep disturbances among medical students in the Middle East and North Africa: a systematic review and meta-analysis. J Glob Health. 2025;15:04099.

Supplementary Table S1: PRISMA checklist 2020.
Supplementary Box S1: Search strategy.
Supplementary Table S2: Characteristics of the included studies.
Supplementary Table S3: Evidence mapping of the included studies on the prevalence of sleep disorders among medical students in MENA.
Figure S1: Pooled prevalence of poor sleep quality in MENA medical students.
Figure S2: Pooled Pittsburgh Sleep Quality Index (PSQI) mean scores for poor sleep quality in MENA medical students.
Figure S3: Pooled prevalence of insufficient sleep duration in MENA medical students.
Figure S4: Pooled mean scores of insufficient sleep duration in MENA medical students.
Figure S5: Pooled prevalence of Excessive Daytime Sleepiness (EDS) in MENA medical students.
Figure S6: Pooled Epworth Sleepiness Scale (ESS) mean scores for Excessive Daytime Sleepiness (EDS) in MENA medical students.
Supplementary Table S4: Meta-analysis of sleep disturbances prevalence among medical students in MENA countries.
Supplementary Table S5: Meta-analysis of sleep disorders mean scores and mean sleeping time in MENA medical students.
Supplementary Table S6: Quality assessment of the included studies.

Table S1: PRISMA checklist 2020

Section and Topic	Item #	Checklist item	Location where item is reported Page number
**TITLE**			
Title	1	Identify the report as a systematic review.	p 1
**ABSTRACT**			
Abstract	2	See the PRISMA 2020 for Abstracts checklist.	Supp p 5
**INTRODUCTION**			
Rationale	3	Describe the rationale for the review in the context of existing knowledge.	p 4-5
Objectives	4	Provide an explicit statement of the objective(s) or question(s) the review addresses.	p 5
**METHODS**			
Eligibility criteria	5	Specify the inclusion and exclusion criteria for the review and how studies were grouped for the syntheses.	P 6-8
Information sources	6	Specify all databases, registers, websites, organisations, reference lists and other sources searched or consulted to identify studies. Specify the date when each source was last searched or consulted.	P 6
Search strategy	7	Present the full search strategies for all databases, registers and websites, including any filters and limits used.	P 6, Supp Box S1 p 6-7
Selection process	8	Specify the methods used to decide whether a study met the inclusion criteria of the review, including how many reviewers screened each record and each report retrieved, whether they worked independently, and if applicable, details of automation tools used in the process.	p 7-8
Data collection process	9	Specify the methods used to collect data from reports, including how many reviewers collected data from each report, whether they worked independently, any processes for obtaining or confirming data from study investigators, and if applicable, details of automation tools used in the process.	p 7-8
Data items	10a	List and define all outcomes for which data were sought. Specify whether all results that were compatible with each outcome domain in each study were sought (e.g. For all measures, time points, analyses), and if not, the methods used to decide which results to collect.	p 6-7
	10b	List and define all other variables for which data were sought (e.g. Participant and intervention characteristics, funding sources). Describe any assumptions made about any missing or unclear information.	p 6-7
Study risk of bias assessment	11	Specify the methods used to assess risk of bias in the included studies, including details of the tool(s) used, how many reviewers assessed each study and whether they worked independently, and if applicable, details of automation tools used in the process.	p 8
Effect measures	12	Specify for each outcome the effect measure(s) (e.g. Risk ratio, mean difference) used in the synthesis or presentation of results.	p 8

Section and Topic	Item #	Checklist item	Location where item is reported Page number
Synthesis methods	13a	Describe the processes used to decide which studies were eligible for each synthesis (e.g. Tabulating the study intervention characteristics and comparing against the planned groups for each synthesis (item #5)).	p 8-9
	13b	Describe any methods required to prepare the data for presentation or synthesis, such as handling of missing summary statistics, or data conversions.	p 8-10
	13c	Describe any methods used to tabulate or visually display results of individual studies and syntheses.	p 8-10
	13d	Describe any methods used to synthesize results and provide a rationale for the choice(s). If meta-analysis was performed, describe the model(s), method(s) to identify the presence and extent of statistical heterogeneity, and software package(s) used.	p 8-10
	13e	Describe any methods used to explore possible causes of heterogeneity among study results (e.g. Subgroup analysis, meta-regression).	p 8-10
	13f	Describe any sensitivity analyses conducted to assess robustness of the synthesized results.	p 8-10
Reporting bias assessment	14	Describe any methods used to assess risk of bias due to missing results in a synthesis (arising from reporting biases).	p 8-11
Certainty assessment	15	Describe any methods used to assess certainty (or confidence) in the body of evidence for an outcome.	p 11
**RESULTS**			
Study selection	16a	Describe the results of the search and selection process, from the number of records identified in the search to the number of studies included in the review, ideally using a flow diagram.	p 12
	16b	Cite studies that might appear to meet the inclusion criteria, but which were excluded, and explain why they were excluded.	P 12-22
Study characteristics	17	Cite each included study and present its characteristics.	Supp, Table S2 p 8-16
Risk of bias in studies	18	Present assessments of risk of bias for each included study.	Supp table S6 p 34-40
Results of individual studies	19	For all outcomes, present, for each study: (a) summary statistics for each group (where appropriate) and (b) an effect estimate and its precision (e.g. Confidence/credible interval), ideally using structured tables or plots.	Supp table S3 p 17-22, Fig S1-S6 p 23-27
Results of syntheses	20a	For each synthesis, briefly summarise the characteristics and risk of bias among contributing studies.	Fig 2 p 23
	20b	Present results of all statistical syntheses conducted. If meta-analysis was done, present for each the summary estimate and its precision (e.g. Confidence/credible interval) and measures of statistical heterogeneity. If comparing groups,	Supp, Table S4-S6 p 28-

Section and Topic	Item #	Checklist item	Location where item is reported Page number
		describe the direction of the effect.	33
	20c	Present results of all investigations of possible causes of heterogeneity among study results.	P 13-22<br>Supp, Table S4-S6 p 28-33
	20d	Present results of all sensitivity analyses conducted to assess the robustness of the synthesized results.	P 13-22<br>Supp, Table S4-S6 p 28-33
Reporting biases	21	Present assessments of risk of bias due to missing results (arising from reporting biases) for each synthesis assessed.	P 23, Fig 3
Certainty of evidence	22	Present assessments of certainty (or confidence) in the body of evidence for each outcome assessed.	P 23-24
**DISCUSSION**			
Discussion	23a	Provide a general interpretation of the results in the context of other evidence.	P 25
	23b	Discuss any limitations of the evidence included in the review.	P 25-30
	23c	Discuss any limitations of the review processes used.	P 25-30
	23d	Discuss implications of the results for practice, policy, and future research.	P 25-30
**OTHER INFORMATION**			
Registration and protocol	24a	Provide registration information for the review, including register name and registration number, or state that the review was not registered.	P 6
	24b	Indicate where the review protocol can be accessed, or state that a protocol was not prepared.	P 6
	24c	Describe and explain any amendments to information provided at registration or in the protocol.	N/A
Support	25	Describe sources of financial or non-financial support for the review, and the role of the funders or sponsors in the review.	P 32
Competing interests	26	Declare any competing interests of review authors.	P 32
Availability of data, code and other materials	27	Report which of the following are publicly available and where they can be found: template data collection forms; data extracted from included studies; data used for all analyses; analytic code; any other materials used in the review.	P 33

From: Page MJ, mckenzie JE, Bossuyt PM, Boutron I, Hoffmann TC, Mulrow CD, et al. The PRISMA 2020 statement: an updated guideline for reporting systematic reviews. BMJ 2021;372:n71. Doi: 10.1136/bmj.n71.For more information, visit: http://www.prisma-statement.org/

PRIMSA Abstract Checklist

Topic	No.	Item	Reported?
**TITLE**			
**Title**	1	Identify the report as a systematic review.	Yes
**BACKGROUND**			
**Objectives**	2	Provide an explicit statement of the main objective(s) or question(s) the review addresses.	Yes
**METHODS**			
**Eligibility criteria**	3	Specify the inclusion and exclusion criteria for the review.	Yes
**Information sources**	4	Specify the information sources (e.g. databases, registers) used to identify studies and the date when each was last searched.	Yes
**Risk of bias**	5	Specify the methods used to assess risk of bias in the included studies.	Yes
**Synthesis of results**	6	Specify the methods used to present and synthesize results.	Yes
**RESULTS**			
**Included studies**	7	Give the total number of included studies and participants and summarise relevant characteristics of studies.	Yes
**Synthesis of results**	8	Present results for main outcomes, preferably indicating the number of included studies and participants for each. If meta-analysis was done, report the summary estimate and confidence/credible interval. If comparing groups, indicate the direction of the effect (i.e. which group is favoured).	Yes
**DISCUSSION**			
**Limitations of evidence**	9	Provide a brief summary of the limitations of the evidence included in the review (e.g. study risk of bias, inconsistency and imprecision).	Yes
**Interpretation**	10	Provide a general interpretation of the results and important implications.	Yes
**OTHER**			
**Funding**	11	Specify the primary source of funding for the review.	Yes
**Registration**	12	Provide the register name and registration number.	Yes

From: Page MJ, McKenzie JE, Bossuyt PM, Boutron I, Hoffmann TC, Mulrow CD, et al. The PRISMA 2020 statement: an updated guideline for reporting systematic reviews. MetaArXiv. 2020, September 14. DOI: 10.31222/osf.io/v7gm2. For more information, visit: www.prisma-statement.org

Box S1: Search strategy

Search platform	Date of search	No of papers screened
**Pubmed**	15/02/2022	1037
("Sleep"[Mesh Terms] Or "Sleep Wake Disorders"[Mesh Terms] Or "Sleep*"[Text Word] Or Insomnia [Text Word] Or Somnolence [Text Word]) And ("Students, Medical"[Mesh Terms] Or ("Medical"[Text Word] And "Students"[Text Word]) Or "Medical Student*"[Text Word] Or ("Medical"[Text Word] And "Student"[Text Word])		
**Web of science**	15/02/2022	1009
**(**Sleep* Or Insomnia Or Somnolence) And ("Medical" And "Students") Or "Medical Student*")<br>(Sleep* Or Insomnia Or Somnolence) And ("Medical Student*")		
**Google scholar**	4/11/2022-13/5/2024	1361

Search 1a:
<u>With all of the words:</u> ~Sleep Quality
<u>Exact phrase</u>: Medical students
<u>With at least one of the words:</u> MENA "Middle East" "North Africa" Algeria Bahrain Djibouti Egypt Iraq Jordan Kuwait Lebanon Libya Morocco Oman

Search 1b:
<u>With all of the words</u>: ~Sleep Quality
<u>Exact phrase</u>: Medical Students
<u>With at least one of the words:</u> GCC "Gulf Cooperation Council" Maghreb Pakistan Palestine Qatar "Saudi Arabia" KSA Sudan Syria Tunisia "United Arab Emirates" UAE Yemen

Search 2a:
<u>With all of the words</u>: ~Sleep Quantity ~Duration
<u>Exact phrase</u>: Medical Students
<u>With at least one of the words:</u> MENA "Middle East" "North Africa" Algeria Bahrain Djibouti Egypt Iraq Jordan Kuwait Lebanon Libya Morocco Oman

Search 2b:
<u>With all of the words</u>: ~Sleep Quantity ~Duration
<u>Exact phrase</u>: Medical Students
<u>With at least one of the words:</u> GCC "Gulf Cooperation Council" Maghreb Pakistan Palestine Qatar "Saudi Arabia" KSA Sudan Syria Tunisia "United Arab Emirates" UAE Yemen

Search 3a:
<u>With all of the words</u>: ~Excessive Daytime Sleepiness ~EDS
<u>Exact phrase</u>: Medical Students
<u>With at least one of the words:</u> MENA "Middle East" "North Africa" Algeria Bahrain Djibouti Egypt Iraq Jordan Kuwait Lebanon Libya Morocco Oman

Search 3b:
<u>With all of the words</u>: ~Excessive Daytime Sleepiness ~EDS
<u>Exact phrase</u>: Medical Students
<u>With at least one of the words:</u> GCC "Gulf Cooperation Council" Maghreb Pakistan Palestine Qatar "Saudi Arabia" KSA Sudan Syria Tunisia "United Arab Emirates" UAE Yemen

Search 4a:
<u>With all of the words</u>: ~Sleep Disturbance
<u>Exact phrase</u>: Medical Students
<u>With at least one of the words:</u> MENA "Middle East" "North Africa" Algeria Bahrain Djibouti Egypt Iraq Jordan Kuwait Lebanon Libya Morocco Oman

Search 4b:
<u>With all of the words</u>: ~Sleep Disturbance
<u>Exact phrase</u>: Medical Students

With at least one of the words: GCC “Gulf Cooperation Council” Maghreb Pakistan Palestine Qatar “Saudi Arabia” KSA Sudan Syria Tunisia “United Arab Emirates” UAE Yemen

Search 5a:
With all of the words: ~Sleep Deprivation
Exact phrase: Medical Students
With at least one of the words: MENA "Middle East" "North Africa" Algeria Bahrain Djibouti Egypt Iraq Jordan Kuwait Lebanon Libya Morocco Oman

Search 5b:
With all of the words: ~Sleep Deprivation
Exact phrase: Medical Students
With at least one of the words: GCC “Gulf Cooperation Council” Maghreb Pakistan Palestine Qatar “Saudi Arabia” KSA Sudan Syria Tunisia “United Arab Emirates” UAE Yemen

---

Table S2: Characteristics of the included studies

First author, year of publication	Country	Study design	Response rate (%)	Sampling method	Year of data collection	Characteristics of study period category	University year	Medical university	Type of university (private, non-private)	Sex: n (%)	Sample size
**Abdulah, 2018[1]**	Iraq	CS	79.3	Simple random	2017	During regular teaching period	NR	University of Duhok	Public	M:137 (43.3)<br>F: 180 (56.7)	317
**Abdulghani, 2012[2]**	Saudi Arabia	CS	55	NR	2009-2010	During regular teaching period	Years 1-3	King Saud University	Public	M: 307 (62.5)<br>F: 184 (37.5)	491
**Abdulrahman, 2021[3]**	Saudi Arabia	CS	75	NR	NR	During regular teaching period	Years 1-6	Imam Mohammad Ibn Saud Islamic University, King Saud Bin Abdulaziz University for Health Sciences, Alfaisal University, King Saud University, King Abdulaziz University, Qassim University	Public	M: 374 (55.4)<br>F: 301 (44.6)	675
**Abu-Ismail, 2023[4]**	Jordan	CS	24.46	NR	2020	During regular teaching period	Years 1-6	NR	Public	M: 440 (36)<br>F: 783 (64)	1223
**Aftab, 2023[5]**	Saudi Arabia	CS	100	Convenience	2022-2023	During regular teaching period	NR	College of Medicine, Northern Border University, Arar	Public	M: 118 (59)<br>F: 82 (41)	200
**Al ani, 2024[6]**	Iraq	CS	73	Convenience	2023	During regular teaching period	Years 4-6	College of Medicine, University of Fallujah	Public	M: 53 (44.2)<br>F: 67 (55.8)	120
**Al Shamli, 2021[7]**	Oman	CS	42.2	All target population	2020	During regular teaching period	Years 1-7	Sultan Qaboos University	Public	M: 68 (26.9)<br>F: 185 (73.1)	253
**Al Shammari, 2020[8]**	Saudi Arabia	CS	NR	Stratified proportional random sampling	NR	During regular teaching period	Years 4-6	Imam Abdulrahman Bin Faisal University	Public	M: 66 (36.7)<br>F: 114 (63.3)	180
**Al Zahrani, 2016[9]**	Saudi Arabia	CS	NR	NR	NR	During regular teaching period	NR	Prince Sattam Bin Abdulaziz University	Public	M: 161 (100)	161
**Aladhab, 2023[10]**	Saudi Arabia	CS	NR	Random in social media of target samples	2021	NR	Pre-professionals, Years 1-5 and Intern	King Khalid University, King Faisal University, University of Hail, University of Najran, Taif University, King Saud University, King Saud Bin Abdulaziz University -Health Sciences. Northern Border University, Sulaiman AlRajhi University, Umm Al-Qura University, Jazan University, Imam Abdulrahman bin, Faisal University. Vision College, Taibah University. University of Tabuk, Qassim University. Jouf	Public and private	M: 460 (35.5)<br>F: 835 (64.5)	1295

								University, Al Rayyan International University, University of Oregon, Shaqra University, Al-Baha University, Almaarefa University, Dar al Uloom University, Ibn Sina National College, Majmaah University, Batterjee Medical College.			
**Al-Ansari, 2022[11]**	Bahrain	CS	67	Convenience	2019	During regular teaching period	Years 1-6	Arabian Gulf University	Public	M: 208 (32)<br>F: 442 (68)	650
**Alaswad, 2017[12]**	Saudi Arabia	CS	100	Simple random	2018	During regular teaching period	Years 1 and 3	Qassim University	Public	M: 78 (100)<br>F: 72 (100)	150
**Albaker, 2021[13]**	Saudi Arabia	CS	NR	Multiple steps stratified sampling	2018-2020	During regular teaching period	Years 1-5	Majmaah University	Public	M/F	163
**Albhlal, 2017[14]**	Saudi Arabia	CS	91.4	Stratified random	2013-2014	During regular teaching period	Years 1-5	Imam Muhammed bin Saud University	Public	M: 128 (100)	128
**Al-Bukhari, 2016[15]**	Saudi Arabia	CS	97.3	NR	2014	During regular teaching period	Years 1-5	Taibah University	Public	M: 261 (47)<br>F: 294 (53)	555
**Aldahash, 2018[16]**	Saudi Arabia	CS	NR	Random	2018	During regular teaching period	Years 4-6	University of Tabuk	Public	M: 75 (100)	75
**Aldhawyan, 2020[17]**	Saudi Arabia	CS	82.87	NR	2018-2019	During regular teaching period	Year 1	Imam Abdulrahman Bin Faisal University	Public	M: 252 (30)<br>F: 590 (70)	842
**Algarni, 2019[18]**	Saudi Arabia	CS	NR	Random one stage stratified cluster sampling	NR	During regular teaching period	NR	Jazan University	Public	M: 243 (54.7)<br>F: 201 (45.3)	444
**Algarni, 2021[19]**	Saudi Arabia	CS	NR	Stratified	2020	During regular teaching period	Years 1-5	Taibah University Medical College	Public	M: 138 (44.2)<br>F: 174 (55.8)	312
**Alghamdi, 2023[20]**	Saudi Arabia	CS	NR	Convenience	2022	During regular teaching period	Years 1-6	Ibn Sina medical college	Private	M: 241 (100)	241
**Al-Ghamdi, 2015[21]**	Saudi Arabia	CS	93.8	Convenience non-random	2013	During regular teaching period	Years 2-6	Prince Sattam bin Abdulaziz University	Public	M: 167 (100)	167
**Alhazzani, 2018[22]**	Saudi Arabia	CS	25.8	All target population	NR	During regular teaching period	Batches 10,11,12,13	King Saud bin Abdulaziz University	Public	M: 166 (100)	166
**Alhusseini, 2022[23]**	Saudi Arabia	CS	NR	Convenience	NR	During regular teaching period	Years 1-6	Alfaisal University	Public	M: 93 (38.6)<br>F: 148 (61.4)	241
**Ali, 2021[24]**	Saudi Arabia	CS	NR	NR	2017	During regular teaching period	Years 1-3	King Faisal University	Public	M: 91 (44.2)<br>F: 115 (55.8)	206
**Ali, 2023[25]**	Qatar	CS	10.5	All target population	2019	During regular teaching period	NR	Qatar University	Public	M/F	36
**Al-Kandari, 2017[26]**	Kuwait	CS	89.7	NR	2015	During regular teaching period	NR	Kuwait University	Public	M/F	120
**Alkhaibary, 2017[27]**	Saudi Arabia	CS	NR	All target population	2017	During regular teaching period	Years 1-2 and pre-clinical	King Saud bin Abdulaziz University and Al-Imam Muhammad Ibn Saud Islamic University	Public	M: 234 (100)	234
**Al-Khaliq, 2023[28]**	Iraq	CS	NR	Random	2020-2021	During regular teaching period	Years 1-5	University of Baghdad	Public	M: 70 (28)<br>F: 180 (72)	250
**Al-Khani, 2019[29]**	Saudi Arabia	CS	46	NR	2018	During regular teaching period	Preparatory, Years 1-5	Sulaiman Al Rajhi College	Private	M: 72 (76)<br>F: 23 (24)	95

**Almansour, 2020[30]**	Saudi Arabia	CS	NR	Stratified random	2017-2018	During regular teaching period	Years 1-5	King Saud University	Public	M: 215 (50)<br>F: 215 (50)	430
**Almetrek, 2015[31]**	Saudi Arabia	CS	86.9	Stratified	2014	During regular teaching period	NR	King Khalid University	Public	M: 267 (100)	267
**Almojali, 2017[32]**	Saudi Arabia	CS	86	Stratified random	2016	During regular teaching period	Years 1-4	King Saud bin Abdulaziz University	Public	M: 181 (68.8)<br>F: 82 (31.2)	263
**Almutairi, 2017[33]**	Saudi Arabia	CS	NR	All target population	2016	During regular teaching period	Years 1-5	Qassim University College of Medicine	Public and private	M: 327 (47)<br>F: 184 (53)	511
**Alnaser, 2021[34]**	Jordan	CS	NR	Respondent-driven sampling	2021	During COVID-19 pandemic/quarantine	Year 1 to Internship	Al-Balqa Applied University, University of Jordan, Mutah University, Hashemite University, Yarmouk University, and Jordan University for Science and Technology	Public	M: 870 (41.3)<br>F: 1234 (58.7)	2104
**Alnomsi, 2018[35]**	Saudi Arabia	CS	NR	Convenience	2017-2018	During regular teaching period	Years 4-6	University of Tabuk	Private	M: 82 (48.5)<br>F: 87 (51.5)	169
**Alotaibi, 2020[36]**	Saudi Arabia	CS	NR	NR	2019	During regular teaching period	Years 1-3	Imam Muhammad Ibn Saud Islamic University	Public	M: 100 (64.5)<br>F: 182 (35.5)	282
**Alotaibi, 2023[37]**	Saudi Arabia	Cohort	73	NR	2022	During regular teaching period (before and during Ramadan)	Years 1-6	Taif University	Public	M: 111 (50.5)<br>F: 109 (49.5)	220
**Alqahtani, 2017[38]**	Saudi Arabia	CS	85.8	NR	2014	During regular teaching period	Years 1-5	Imam University, King Saud University or King Saud Bin Abdul-Aziz University	Public	M: 145 (60.9)<br>F: 92 (38.7)	237
**Alqarni, 2018[39]**	Saudi Arabia	CS	NR	NR	2017	During regular teaching period	Years 2-6	Taif University	Public	M: 132 (44.1)<br>F: 167 (55.9)	299
**Alqudah, 2022[40]**	Jordan	CS	NR	NR	2018	During regular teaching period	Years 1-6	Jordan University	Public	M/F	299
**Alrasheed, 2023[41]**	Saudi Arabia	CS	100	Simple random	2022	Before and during COVID-19 pandemic quarantine	Year 1 - Intership	University of Tabuk	Public	M: 104 (37.3)<br>F: 175 (62.7)	279
**Alsaggaf, 2016[42]**	Saudi Arabia	CS	95	Random	2011-2012	During regular teaching period	Years 4-6	King Abdulaziz University	Public	M: 127 (42)<br>F: 178 (58)	305
**Al-sayed, 2014[43]**	Saudi Arabia	CS	98	Convenience	2011	During regular teaching period	Years 1-5	King Saud University	Public	M: 371 (51)<br>F: 358 (49)	729
**Al-senaidi, 2022[44]**	Saudi Arabia	CS	NR	NR	NR	During regular teaching period	Years 1-5	Imam University	Public	M: 63 (22.6)<br>F: 216 (77.4)	279
**Alshahrani, 2019[45]**	Saudi Arabia	CS	88	Convenience	2016	During regular teaching period	NR	King Saud University	Public	M/F	200
**Alshumrani, 2023[46]**	Saudi Arabia	CS	NR	NR	2021	During regular teaching period	Years 2-6	King Abdulaziz University	Public	M: 74 (28.5)<br>F: 186 (71.5)	260
**Alsulami, 2019[47]**	Saudi Arabia	CS	46.8	NR	NR	During regular teaching period	Year 2-6	King Abdulaziz University and Batterjee Medical College	Public	M: 292 (41.6)<br>F: 410 (58.4)	702
**Alsumairi, 2022[48]**	Saudi Arabia	CS	NR	Stratified cluster sampling	2021	During regular teaching period	Year 1-Intern	Taif University	Public	M: 209 (56.2)<br>F: 163 (43.8)	372

**Al-ubaidi, 2018[49]**	Bahrain	CS	65	All clerkship-training students	2017	During regular teaching period	First and second clerkship year	Arabian Gulf University, and Royal College of Surgeons in Ireland-Medical University of Bahrain	Public and private	M: 141 (40.6) F: 206 (59.4)	347
**Alzunidi, 2022[50]**	Saudi Arabia	CS	NR	Quota sampling	NR	During regular teaching period	Pre-clinical (4-7) and Clinical (8-13)	Almaarefa University for Science and Technology	Public	M/F	99
**Amin, 2016[51]**	Saudi Arabia	CS	88.8	Simple random	2012	During regular teaching period	Years 1-5	King Saud University	NR	M: 196 (57.5) F: 145 (42.5)	341
**Arsalan, 2015[52]**	Pakistan	CS	NR	Convenience	2015	During regular teaching period	NR	Medical College in Karachi	NR	M: 135 (33.33) F: 270 (66.67)	405
**Arshad, 2021[53]**	Pakistan	Cohort	86.4	Randomized consecutive	2019	During regular teaching period	NR	Rawalpindi Medical University	Public	M: 116 (47.93) F: 126 (52.06)	242
**Asiri, 2018[54]**	Saudi Arabia	CS	NR	Simple random	NR	During regular teaching period	NR	King Khalid University	Public	NR	286
**Attal, 2020[55]**	Yemen	CS	NR	Random	2017	Around final exams	Years 3-6	Sana'a University	Public	M: 176 (49) F: 184 (51)	360
**Attal, 2021[56]**	Yemen	CS	90.5	Convenience	2007	During regular teaching period	Years 1-6	Sana'a University	Public	M: 98 (41) F: 142 (59)	240
**Ayub, 2022[57]**	Pakistan	CS	100	Convenience	2020-2021	During COVID-19 pandemic/quarantine	NR	Medical colleges of Lahore and Faisalabad	Public and private	M: 24 (16) F: 126 (84)	150
**Bahammam, 2003[58]**	Saudi Arabia	CS	81	NR	1999	One week before Ramadan	NR	NR	Public	M: 31 (55) F: 25 (45)	56
**Bahammam, 2005[59]**	Saudi Arabia	CS	63	Random	2021	During regular teaching period	Years 1-3	King Saud University	Public	M: 129 (100)	129
**Bahammam, 2012[60]**	Saudi Arabia	CS	83	Systematic random	2009-2010	NR	First (L1), second (L2), third (L3)	King Saud University	Public	M: 273 (66.6) F: 137 (33.4)	410
**Bhatti, 2012[61]**	Pakistan	CS	NR	Random	2010-2011	During regular teaching period	Year 1 and final year	Islamic International Medical College, and IIMC-T Railway General Hospital	Private	M: 30 (50) F: 30 (50)	60
**Bokhari, 2020[62]**	Pakistan	CS	NR	Stratified random	NR	During regular teaching period	Years 1-5	Khawaja Muhammad Safdar Medical College	Public	M: 48 (31.2) F: 106 (68.8)	154
**Butt, 2018[63]**	Pakistan	CS	NR	Purposive sampling	NR	During regular teaching period	Years 1-5	Gujranwala Medical College	Public	M: 33 (22) F: 177 (78)	150
**Chaabna k, 2022[64]**	Qatar	CS	95.6	Quota sampling	2017-2018	During regular teaching period	NR	Weill Cornell Medical University-Qatar	Private	M: 21 (51.2) F: 20 (48.8)	41
**Chahine, 2023[65]**	Lebanon	CS	43.5	Snowball technique	2022	During regular teaching period	Years 6-7 & residency	Lebanese University School of Medicine	Public	M: 94 (41.6) F: 132 (58.4)	226
**El hangouche, 2018[66]**	Morocco	CS	76.2	Random	2017	During regular teaching period	Years 1-5	Faculty of Medicine and Pharmacy	Public	M: 134 (29.3) F: 323 (70.7)	457
**Elwasify, 2016[67]**	Egypt	CS	NR	NR	2015	During regular teaching period	NR	Assiut and Mansoura Universities	Public	M/F	1182
**Ezelarab, 2014[68]**	Egypt	CS	93	Random	2009-2010	During midsemester in their break times	Years 1-6	Ain Shams University	Public	M/F	435
**Fawzy, 2017[69]**	Egypt	CS	100	NR	2015	During regular teaching period	Year 1-6	Assiut University	Public	M: 248 (35) F: 452 (65)	700

**Gassara, 2016[70]**	Tunisia	CS	NR	NR	NR	During regular teaching period	NR	University College of Medicine	Public	M/F	74
**Gemnani, 2020[71]**	Pakistan	CS	NR	Stratified systematic random	2020	During regular teaching period	Years 2-4	SMBB Medical University	Public	M: 119 (46.7)<br>F: 136 (53.3)	255
**Ghabban, 2017[72]**	Saudi Arabia	CS	85.2	NR	2017	Days other than examinations, to avoid stress.	Years 2-6	University of Tabuk	Public	M: 137 (49.5)<br>F: 140 (50.5)	277
**Gulzar, 2023[73]**	Pakistan	CS	NR	NR	NR	During regular teaching period	NR	Ameer-ud-din Medical College, PGMI, and<br>King Edward Medical University	Public	M: 142 (44)<br>F: 181 (56)	323
**Hamed, 2015[74]**	UAE	CS	95	Universal sampling technique	2015	During regular teaching period	Different levels	Ajman University of Science and Technology	Private	M:50 (26)<br>F:140 (74)	190
**Hammad, 2024[75]**	Saudi Arabia	CS	90.4	Simple random	2023	During regular teaching period	NR	Medical colleges from Najran, King Khalid, Jazan universities	Public	M: 178 (52.7)<br>F: 160 (47.3)	338
**Hashmi, 2022[76]**	Pakistan	CS	NR	Convenience	NR	During regular teaching period	Year 3 and 4	HITEC-IMS	Private	M/F	60
**Hassan, 2023[77]**	Pakistan	CS	100	Randon using lottery ticket method	NR	During regular teaching period	Years 1 and 5	Nishtar medical university	Public	M: 52 (50)<br>F: 52 (50)	104
**Huma, 2023[78]**	Pakistan	CS	NR	NR	2021-2022	During COVID-19 pandemic/quarantine	Pre-Clinical and Clinical years	University of Lahore	Private	M/F	200
**Hussain, 2023[79]**	Pakistan	CS	100	Convenience	2023	During regular teaching period	Year 2	Faisalabad Medical University, Punjab.	Public	M: 74 (33)<br>F: 150 (67)	224
**Ibrahim, 2013[80]**	Saudi Arabia	CS	NR	Multistage stratified random sampling	2011-2012	During regular teaching period	2nd-interns	King Abdulaziz University	Public	M: 300 (50.3)<br>F: 297 (49.7)	597
**Ibrahim, 2017[81]**	Saudi Arabia	CS	NR	Multistage stratified sampling	NR	During regular teaching period	Basic and Clinical	King Abdulaziz University	Public	M: 206 (35.7)<br>F: 370 (64.3)	576
**Ibrahim, 2018[82]**	Saudi Arabia	CS	NR	Multistage stratified random sampling	2016-2017	During regular teaching period	Year 2-6	King Abdulaziz University	Public	M/F	610
**Ibrahim, 2023[83]**	Sudan	CS	99.4	Systematic random sampling	2022	During regular teaching period	Years 2-6	University of Khartoum	Public	M: 125 (36.8)<br>F: 215 (63.2)	340
**Ibrahim, 2024[84]**	Sudan	CS	99.4	Systematic random sampling	2022	During regular teaching period	Years 1-6	University of Khartoum, faculty of Medicine.	Public	M: 125 (36.8)<br>F: 215 (63.2)	340
**Irshad, 2022[85]**	Pakistan	CS	NR	NR	2019-2021	During regular teaching period	Year 1-5	Shifa College of Medicine	Private	M/F	284
**Ishaq, 2020[86]**	Pakistan	CS	NR	Convenience	2018	NR	NR	Jinnah Medical and Dental College, Karachi Medical College, Liaquat College of Medicine and Dentistry, and Al-Tibri Medical College	Public and private	M: 80 (26.6)<br>F: 220 (73.3)	300
**Jahangeer, 2021[87]**	Pakistan	CS	99.3 (from manual)<br>100 (from online)	Systematic random sampling	2020	During regular teaching period	Years 1-5	Dow Medical College	Public	M: 108 (18.8)<br>F: 465 (81.2)	573

**Javaid, 2020[88]**	Pakistan	CS	NR	Convenience	2017-2018	During regular teaching period	Years 2-5	King Edward Medical University	Public	M/F	418
**Javed, 2023[89]**	Pakistan	CS	100	Simple random sampling	2022	During regular teaching period	Years 1-5	Azra Naheed Medical College	Private	M: 199 (47.5)<br>F: 220 (52.5)	419
**Joudeh, 2024[90]**	Jordan	CS	21	All target population	2021	During regular teaching period	Years 1-internship	The University of Jordan, Mutah University, Jordan University of Science and Technology, Hashemite University, Yarmouk University and Al-Balqa Applied University.	Public	M: 870 (41.3)<br>F: 1234 (58.7)	2104
**Kazim, 2011[91]**	Pakistan	CS	96.9	Convenience	2011	Assessment Period	NR	Shifa College of Medicine	Private	M: 86 (100)<br>F: 103 (100)	189
**Khan, 2004[92]**	Pakistan	CS	NR	NR	2002	During regular teaching period	Years 1-4	Shifa College of Medicine	Private	M/F	112
**Khan, 2019[93]**	Pakistan	CS	NR	Convenience	2018	During regular teaching period	Years 1-5	University of Lahore	Private	M: 106 (48.2)<br>F: 114 (51.8)	220
**Khan, 2024[94]**	Pakistan	CS	96.3	Convenience sampling	2022	During regular teaching period	Years 1-5	NR	Private and public	M: 237 (61.6)<br>F: 148 (38.4)	274
**Khero, 2019[95]**	Pakistan	CS	NR	NR	2018	During regular teaching period	Years 1-5	Jinnah Medical University	Public	M: 74 (26.33)<br>F: 207 (73.66)	281
**Maalej, 2018[96]**	Tunisia	CS	NR	NR	2015-2016	During regular teaching period	NR	Sfax Medical School	Public	M: 61 (28.3)<br>F: 123 (71.7)	184
**Maheshwari, 2019[97]**	Pakistan	CS	45.54	NR	2019	During regular teaching period	Years 1-5	Public medical university of Karachi	Public	M: 266 (33.37)<br>F: 531 (66.62)	797
**Mahfouz, 2013[98]**	Saudi Arabia	CS	85	Random	NR	During regular teaching period	NR	Jazan University	Public	M: 120 (35.3)<br>F: 220 (64.7)	340
**Malik, 2024[99]**	Pakistan	CS	88	Stratified random sampling	NR	During regular teaching period	Years 1-4	NR	Public	M: 152 (51)<br>F: 148 (49)	300
**Mansour, 2016[100]**	Saudi Arabia	CS	54.2	NR	2013-2014	During regular teaching period	NR	Taibah University	Public	M: 38 (31.1)<br>F: 84 (68.9)	122
**Mansour, 2020[101]**	Saudi Arabia	CS	56	NR	NR	During regular teaching period	Premed-year 4	Qassim University	Public	M: 123 (44)<br>F: 156 (56)	279
**Margolis, 2004[102]**	UAE	CS	84	NR	2003	Excessive sleepiness in the week prior to the commencement of Ramadan	Years 1-4	University Faculty of Medicine and Health Sciences	Public	M: 39 (28)<br>F: 98 (72)	137
**Meo, 2022[103]**	Pakistan	CS	NR	Stratified random sampling	2020	During COVID-19 pandemic+ during quarantine	Years 1-5	King Saud University	Private	M: 271 (34.7)<br>F: 511 (65.3)	410
**Mirghani, 2015a[104]**	Sudan	CS	68	All target population	2014	During regular teaching period	Clinical stage	Omdurman University	Public	M: 10 (9.2)<br>F: 98 (90.8)	108
**Mirghani, 2015b[105]**	Sudan	Case–control	81.6	All target population	NR	During regular teaching period	5-6	University of Omdurman and Bahri	Public	M: 38 (27.1)<br>F: 102 (72.9)	140

**Mirza, 2021[106]**	Saudi Arabia	CS	17.5	All target population	2019	During regular teaching period	Years 2-6	Umm Al-Qura University	Public	M: 106 (45.89) F: 125 (54.11)	231
**Moalla, 2016[107]**	Tunisia	CS	NR	NR	NR	Assessment Period	NR	University of Sfax	Public	The sex ratio M/F was 0.66	NR
**Mohamed, 2021[108]**	Egypt	CS	NR	Simple random sampling	2018-2019	During regular teaching period	Years 1-6	Suez Canal University	Public	M: 43 (28.7) F: 107 (71.3)	150
**Mohamed, 2024[109]**	Saudi Arabia	CS	NR	Convenience	2021-2022	During regular teaching period	Years 4-6	KSAU-HS and KSU universities	Public	M: 187 (77) F: 56 (23)	243
**Mohammed, 2020[110]**	Sudan	CS	NR	Random	2020	During regular teaching period	NR	University of science and technology	Private	M: 40 (40) F: 60 (60)	100
**Nadeem, 2018[111]**	Pakistan	CS	85.8	NR	2017	During regular teaching period	NR	Rawalpindi Medical College, Federal Medical and Dental College, Ayub Medical College, King Edward Medical University, and Shifa College of Medicine	Public and private	M: 74 (27.8) F: 192 (72.2)	266
**Naeem, 2014[112]**	UAE	CS	NR	Random	NR	NR	NR	Ras Al Khaimah Medical and Health Sciences University	Public	M: 30 (25.6) F: 87 (74.4)	117
**Naeem, 2018[113]**	Pakistan	CS	NR	NR	NR	During regular teaching period	NR	King Edward Medical University	Public	M: 50 (50) F: 50 (50)	100
**Naseer, 2019[114]**	Pakistan	CS	NR	NR	2018	During regular teaching period	NR	Dow Medical College and Pakistan Air Force-Karachi Institute of Economics and Technology	Public	M/F	137
**Naveed, 2023[115]**	Pakistan	CS	NR	Convenience	2020	During COVID-19 pandemic/quarantine	NR	HITEC-IMS	Private	M: 81 (55.8) F: 65 (44.2)	146
**Nisar, 2019[116]**	Pakistan	CS	NR	Consecutive	2017-2018	During regular teaching period	Years 1-6	Three different medical schools in Karachi	NR	M: 141 (32) F: 289 (65.7)	440
**Qaiser, 2018[117]**	Saudi Arabia	CS	NR	Convenience	2011-2013	During regular teaching period	Years 2-4	King Saud Bin Abdulaziz University	Public	M: 101 (100)	101
**Qanash, 2021[118]**	Saudi Arabia	CS	61.8	NR	2019	During regular teaching period	NR	King Saud bin Abdulaziz University for Health Sceinces, King Abdulaziz University, and Jeddah University	Public	M/F	193
**Quronfulah, 2023 [119]**	Saudi Arabia	CS	NR	Convenience	2022	During regular teaching period	Students, interns, specialists	NR	NR	M: 308 (39.9) F: 463 (60.1)	156
**Rafeeq 2021[120]**	Pakistan	CS	100	Convenience	2019-2020	During regular teaching period	Years 1-4	Lahore Medical College	Private	M: 138 (46.8) F: 157 (53.2)	295
**Rafiq, 2017[121]**	Pakistan	CS	NR	NR	NR	During regular teaching period	NR	Nawaz Sharif Medical College	Public	M: 14 (17.5) F: 66 (83.6)	80
**Rejeb, 2018[122]**	Tunisia	CS	79	NR	2017	During regular teaching period	Pre-clinical and clinical years	Medical University of Sousse	Public	M: 78 (25.2) F: 232 (74.8)	310
**Safhi, 2020[123]**	Saudi Arabia	CS	NR	Stratified random sampling	2018	During regular teaching period	Years 2-6	King Saud University	Public	M: 165 (50.6) F: 161 (49.4)	326
**Saguem, 2022[124]**	Tunisia	CS	18.83	NR	2020	During COVID-19 pandemic+ during quarantine	Years 1-5	Faculty of Medicine of Sousse	Public	M: 44 (17.5) F: 207 (82.5)	251
**Saif, 2024[125]**	Pakistan	CS	80	Purposive sampling	NR	During assessment period	Years 1-5	Central Park Medical College, Lahore, Pakistan,	Private	M: 43 (42) F: 59 (58)	102

**Salama, 2017[126]**	Egypt	CS	100	Random stratification with allocation	2017	During regular teaching period	Years 1-6	Menoufia University	Public	M: 185 (36.6) F: 320 (63.4)	505
**Sameer, 2020[127]**	Pakistan	CS	73.9	Convenience	2018	During regular teaching period	Years 1-5	Public-sector university	Public	M: 194 (44) F: 247 (56)	441
**Satti, 2019[128]**	Pakistan	CS	74.71	NR	2019	During regular teaching period	Years 4	Rawalpindi Medical University	Public	M: 94 (42.9) F: 125 (57.1)	219
**Shafique, 2021[129]**	Pakistan	CS	NR	NR	2020	During regular teaching period	Years 1-5	Quaid-eAzam Medical College	Public	M: 49 (49) F: 51 (51)	100
**Shehata, 2020[130]**	Saudi Arabia	CS	NR	NR	2019-2020	During regular teaching period	Years 4-6 and Intern year	King Khalid University	Public	M: 256 (52.2) F: 234 (47.8)	490
**Shehata, 2022[131]**	Egypt	CS	NR	Randomized through selection of two random groups	2021-2022	During regular teaching period	Years 2-6 and house officers	Menoufia University	Public	M: 416 (40.3) F: 617 (59.7)	1033
**Shukri, 2019[132]**	Saudi Arabia	CS	100	Random	2017-2018	During regular teaching period	Years 4-6	Taif University	Public	M: 146 (55.9) F: 115 (44.1)	261
**Siddiqui, 2016[133]**	Saudi Arabia	CS	85	Convenience	2015	During regular teaching period	Academic levels 3-12	King Khalid University	Public	M: 206 (64.8) F: 112 (35.2)	318
**Surani, 2015[134]**	Pakistan	CS	77.5	Convenience	2013	During regular teaching period	Years 1-5	Dow Medical College, Sindh Medical College, Aga Khan University Medical College, Ziauddin Medical College and Baqai Medical College	Public	M: 204 (40.5) F: 300 (59.5)	504
**Suwayri, 2016[135]**	Saudi Arabia	CS	26.3	All target population	2016	During regular teaching period	Years 1-6	Al Imam Mohammed Ibn Saud University	Public	M: 111 (65.3) F: 59 (34.7)	170
**Swed, 2023[136]**	Syria	CS	97.2	NR	2022	During regular teaching period	NR	Damascus university, Tishreen university, Al-Baath university, Aleppo university and Tartus university.	Public	M: 717 (24.8) F: 2174 (75.2)	2891
**Tahir, 2020[137]**	Pakistan	CS	NR	Simple random	2017	During regular teaching period	Years 1-2	King Edward Medical University	Public	M: 98 (43.2) F: 129 (56.8)	227
**Talih, 2018[138]**	Lebanon	CS	42.7	All target population	2016	During regular teaching period	Years 1-4	American University of Beirut Medical Center	Private	M: 88 (51.2) F: 84 (48.8)	172
**Tauseef, 2021[139]**	Pakistan	CS	99.6	Convenience	2020-2021	During regular teaching period	Years 1-5	Allama Iqbal Medical College	Public	M: 109 (33.8) F: 213 (66.2)	322
**Thobani, 2023[140]**	Pakistan	CS	NR	NR	2019-2020	During regular teaching period	Years 1-5	Aga Khan University	Private	M: 178 (58.9) F: 124 (41.1)	302
**Toubasi, 2021[141]**	Jordan	CS	NR	NR	2020	During regular teaching period	Years 2-3	University of Jordan	Public	M: 132 (46.8) F: 150 (53.2)	282
**Traore, 2023[142]**	Morocco	CS	12.4	NR	2020	During regular teaching period	Years 1-7	Faculty of Medicine and Pharmacy of Casablanca	Public	M: 203 (40.1) F: 303 (59.9)	506
**Usman, 2017[143]**	Pakistan	CS	100	NR	NR	During regular teaching period	NR	Dow University of health Sciences and Jinnah Medical and dental College	Public and private	M: 87 (32) F: 183 (68)	270
**Varanasi, 2020[144]**	UAE	CS	NR	All target population	NR	During regular teaching period	NR	RAK Medical and Health Sciences University	Public	M/F	113

**Vohra, 2019[145]**	Saudi Arabia	CS	NR	Stratified random	2014-2015	During regular teaching period	Years 1-3	King Saud University	Public	M: 101 (100)	101
**Waqas, 2015[146]**	Pakistan	CS	93.9	Stratified random	2014	During regular teaching period	Years 1-5	Military Hospital Lahore Medical College and Institute of Dentistry	Private	M: 115 (43.7)<br>F: 148 (56.3)	263
**Yassin, 2020[147]**	Jordan	CS	29.7	All target population	NR	During regular teaching period	Years 2-6	Jordan University of Science and Technology or Yarmouk University	Public	M: 493 (47.4)<br>F: 548 (52.6)	1041
**Zafar, 2020 a[148]**	Saudi Arabia	CS	NR	Stratified random	NR	NR	Years 1-5	Dammam Medical College	Public	M: 51 (34.2)<br>F: 98 (65.8)	149
**Zafar, 2020 b[149]**	Sudan	CS	NR	Simple random	NR	During regular teaching period	Years 1-5	Dammam Medical College	Public and private	M: 73 (36.7)<br>F: 126 (63.3)	199
**Zainab, 2020[150]**	Pakistan	CS	NR	Purposive	2016-2017	NR	Years 1-5	Liaquat National Medical College	Private	M: 111 (30.8)<br>F: 249 (69)	360

Note: CS= Cross-sectional; NR= Not reported; M=Male; F=Female; UAE= United Arab Emirates; HITEC-IMS = HITEC Institute of Medical Sciences; SMBB = Shaheed Mohatarma Benazir Bhutto; IIMCT = Islamic International Medical College Trust; RAK= Ras Al-Khaimah; KSAU-HS = King Saud bin Abdulaziz University for Health Sciences; KSU = King Saud University.

Table S3: Evidence mapping of the included studies on the prevalence of sleep disorders among medical students in MENA

		Poor sleep quality					Insufficient sleep duration			Excessive daytime sleepiness				
	**Country**	**Prevalence**	**Mean score**	**Instrument used**	**Academic Performance**	**Stress**	**Prevalence**	**Mean score**	**Instrument used**	**Prevalence**	**Mean Score**	**Instrument used**	**Academic Performance**	**Stress**
**Abdulah, 2018**[1]	Iraq								SLEEP-50					
**Abdulghani, 2012**[2]	Saudi Arabia								Self-developed questionnaire			ESS		
**Abdulrahman, 2021**[3]	Saudi Arabia								Self-developed questionnaire†					
**Abu-Ismail, 2023**[4]	Jordan			CHERRIES					NR					
**Aftab, 2023**[5]	Saudi Arabia			PSQI										
**Al Ani, 2024**[6]	Iraq			PSQI										
**Al Shamli, 2021**[7]	Oman			PSQI										
**Al Shammari, 2020**[8]	Saudi Arabia			PSQI								ESS		
**Al Zahrani, 2016**[9]	Saudi Arabia								NR			ESS		
**Aladhab, 2023**[10]	Saudi Arabia											ESS		
**Al-Ansari, 2022**[11]	Bahrain			PSQI										
**Alaswad, 2017**[12]	Saudi Arabia		ϕ	Questionnaire adapted from the University of Kentucky College of Medicine†					Questionnaire adapted from the University of Kentucky College of Medicine†					
**Albaker, 2021[13]**	Saudi Arabia								Self-developed questionnaire†					
**Albhlal, 2017**[14]	Saudi Arabia								PSQI					
**Al-bukhari, 2016**[15]	Saudi Arabia								Self-developed questionnaire					
**Aldahash, 2018**[16]	Saudi Arabia								Self-developed questionnaire					
**Aldhawyan, 2020**[17]	Saudi Arabia			PSQI					PSQI					
**Algarni, 2019**[18]	Saudi Arabia								Self-developed questionnaire					
**Algarni, 2021**[19]	Saudi Arabia			PSQI					PSQI					
**Alghamdi, 2023**[20]	Saudi Arabia			SQS										
**Al-Ghamdi, 2015[21]**	Saudi Arabia								Self-developed questionnaire†					
**Alhazzani, 2018**[22]	Saudi Arabia			Questionnaire adapted from					Questionnaire adapted from the					

				the Groninger Sleep Quality Questionnaire					Groninger Sleep Quality Questionnaire					
**Alhusseini, 2022**[23]	Saudi Arabia			PSQI										
**Ali, 2021**[24]	Saudi Arabia								Self-developed questionnaire					
**Ali, 2023**[25]	Qatar		*	PSQI										
**Al-Kandari, 2017**[26]	Kuwait		Median	PSQI										
**Alkhaibary, 2017**[27]	Saudi Arabia								Self-developed questionnaire					
**AL-Khaliq, 2023**[28]	Iraq								NR					
**Al-Khani, 2019**[29]	Saudi Arabia			PSQI										
**Almansour, 2020**[30]	Saudi Arabia								Self-developed questionnaire					
**Almetrek, 2015**[31]	Saudi Arabia			PSQI										
**Almojali, 2017**[32]	Saudi Arabia			PSQI										
**Almutairi, 2017**[33]	Saudi Arabia								Self-developed questionnaire†					
**Alnaser, 2021**[34]	Jordan			SQS										
**Alnomsi, 2018[35]**	Saudi Arabia			PSQI					PSQI					
**Alotaibi, 2020**[36]	Saudi Arabia			PSQI										
**Alotaibi, 2023**[37]	Saudi Arabia								Self-developed questionnaire†					
**AlQahtani, 2017**[38]	Saudi Arabia								PSQI			ESS		
**Alqarni, 2018[39]**	Saudi Arabia			PSQI					PSQI					
**Alqudah, 2022**[40]	Jordan								NR			ESS		
**Alrasheed, 2023**[41]	Saudi Arabia								Self-developed questionnaire					
**Alsaggaf, 2016**[42]	Saudi Arabia			PSQI								ESS		
**Al-Sayed, 2014**[43]	Saudi Arabia			Self-developed questionnaire					Self-developed questionnaire					
**Al-Senaidi, 2022**[44]	Saudi Arabia			Self-developed questionnaire					Self-developed questionnaire					
**Alshahrani, 2019**[45]	Saudi Arabia			PSQI										
**Alshumrani, 2023**[46]	Saudi Arabia			PSQI					PSQI					
**Alsulami, 2019**[47]	Saudi Arabia			PSQI										
**Alsumairi, 2022**[48]	Saudi Arabia			PSQI										
**Al-Ubaidi, 2018**[49]	Bahrain								Self-developed questionnaire					

**Alzunidi, 2022**[50]	Saudi Arabia								Self-developed questionnaire†					
**Amin, 2016**[51]	Saudi Arabia								Self-developed questionnaire†					
**Arsalan, 2015**[52]	Pakistan			PSQI										
**Arshad, 2021**[53]	Pakistan			PSQI										
**Asiri, 2018**[54]	Saudi Arabia			PSQI										
**Attal, 2020**[55]	Yemen											ESS		
**Attal, 2021**[56]	Yemen			PSQI					PSQI					
**Ayub, 2022**[57]	Pakistan		ϕ	NR										
**Bahammam, 2003**[58]	Saudi Arabia											ESS		
**Bahammam, 2005**[59]	Saudi Arabia								Self-developed questionnaire			ESS		
**Bahammam, 2012**[60]	Saudi Arabia											ESS		
**Bhatti, 2012**[61]	Pakistan								Self-developed questionnaire					
**Bokhari, 2020**[62]	Pakistan			PSQI								ESS		
**Butt, 2018**[63]	Pakistan													
**Chaabna K, 2022**[64]	Qatar								PSQI					
**Chahine, 2023**[65]	Lebanon			PSQI								ESS		
**El Hangouche, 2018**[66]	Morocco			PSQI								ESS		
**Elwasify, 2016**[67]	Egypt			PSQI										
**EzElArab, 2014[68]**	Egypt								Self-developed questionnaire†			ESS		
**Fawzy, 2017**[69]	Egypt		Ω	PSQI					PSQI					
**Gassara, 2016**[70]	Tunisia			PSQI										
**Gemnani, 2020**[71]	Pakistan								Self-developed questionnaire			ESS		
**Ghabban, 2017**[72]	Saudi Arabia											ESS		
**Gulzar, 2023**[73]	Pakistan								Self-developed questionnaire					
**Hamed, 2015**[74]	UAE								Self-developed questionnaire					
**Hammad, 2024**[75]	Saudi Arabia			PSQI										
**Hashmi, 2022**[76]	Pakistan								NR					
**Hassan, 2023**[77]	Pakistan			PSQI										
**Huma, 2023**[78]	Pakistan		ϕ	Self-developed questionnaire				*	PSQI					
**Hussain, 2023**[79]	Pakistan			PSQI										
**Ibrahim, 2013**[80]	Saudi Arabia								Self-developed questionnaire†					
**Ibrahim, 2017**[81]	Saudi Arabia			PSQI								ESS		

**Ibrahim, 2018**[82]	Saudi Arabia			PSQI					PSQI					
**Ibrahim, 2023**[83]	Sudan			Self-developed questionnaire					Self-developed questionnaire					
**Ibrahim, 2024**[84]	Sudan								Questionnaire					
**Irshad, 2022**[85]	Pakistan								BCSQ-12-SS					
**Ishaq, 2020**[86]	Pakistan										Ω	ESS		
**Jahangeer, 2021**[87]	Pakistan	*		Self-developed questionnaire										
**Javaid, 2020**[88]	Pakistan			PSQI								ESS		
**Javed, 2023**[89]	Pakistan			PSQI					PSQI					
**Joudeh, 2024**[90]	Jordan		ϕ	SQS										
**Kazim, 2011**[91]	Pakistan								Self-developed questionnaire					
**Khan, 2004**[92]	Pakistan							*	Self-developed questionnaire					
**Khan, 2019**[93]	Pakistan								Self-developed questionnaire					
**Khan, 2024**[94]	Pakistan			PSQI										
**Khero, 2019**[95]	Pakistan			PSQI					PSQI					
**Maalej, 2018**[96]	Tunisia		*	PSQI										
**Maheshwari, 2019**[97]	Pakistan			PSQI					PSQI					
**Mahfouz, 2013**[98]	Saudi Arabia			PSQI				*	PSQI					
**Malik, 2024**[99]	Pakistan		ϕ	GSS										
**Mansour, 2016**[100]	Saudi Arabia								NR					
**Mansour, 2020**[101]	Saudi Arabia								Self-developed questionnaire					
**Margolis, 2004**[102]	UAE											ESS		
**Meo, 2022**[103]	Pakistan		*	PSQI					PSQI					
**Mirghani, 2015 a**[104]	Sudan								ESS			ESS		
**Mirghani, 2015 b**[105]	Sudan			PSQI					PSQI					
**Mirza, 2021**[106]	Saudi Arabia								Self-developed questionnaire					
**Moalla, 2016**[107]	Tunisia	*	*	PSQI										
**Mohamed, 2021**[108]	Egypt			PSQI										
**Mohamed, 2024**[109]	Saudi Arabia			PSQI					PSQI					
**Mohammed, 2020**[110]	Sudan								Self-developed questionnaire					
**Nadeem, 2018**[111]	Pakistan			PSQI					PSQI					
**Naeem, 2014**[112]	UAE											ESS		
**Naeem, 2018**[113]	Pakistan								Self-developed questionnaire†					
**Naseer, 2019**[114]	Pakistan								PSQI					

**Naveed, 2023**[115]	Pakistan		*	PSQI										
**Nisar, 2019**[116]	Pakistan			PSQI					PSQI					
**Qaiser, 2018**[117]	Saudi Arabia			PSQI					PSQI			ESS		
**Qanash, 2021**[118]	Saudi Arabia			PSQI										
**Quronfulah, 2023**[119]	Saudi Arabia		ϕ	SQS										
**Rafeeq 2021**[120]	Pakistan								Self-developed questionnaire*					
**Rafiq, 2017**[121]	Pakistan								Self-developed questionnaire†					
**Rejeb, 2018**[122]	Tunisia			PSQI					PSQI					
**Safhi, 2020**[123]	Saudi Arabia			PSQI					PSQI					
**Saguem, 2022**[124]	Tunisia			PSQI					PSQI					
**Saif, 2024**[125]	Pakistan						Π		Self-developed questionnaire†					
**Salama, 2017**[126]	Egypt			PSQI					PSQI			Questionnaire		
**Sameer, 2020**[127]	Pakistan								GHQ-12			ESS		
**Satti, 2019**[128]	Pakistan			PSQI										
**Shafique, 2021[129]**	Pakistan			PSQI					PSQI					
**Shehata, 2020[130]**	Saudi Arabia			PSQI										
**Shehata, 2022**[131]	Egypt			PSQI					PSQI					
**Shukri, 2019**[132]	Saudi Arabia			QoL					QoL					
**Siddiqui, 2016**[133]	Saudi Arabia			PSQI					PSQI					
**Surani, 2015**[134]	Pakistan			PSQI								ESS		
**Suwayri, 2016**[135]	Saudi Arabia		Median	PSQI										
**Swed, 2023**[136]	Syria								NR					
**Tahir, 2020**[137]	Pakistan			PSQI										
**Talih, 2018**[138]	Lebanon								Self-developed questionnaire					
**Tauseef, 2021**[139]	Pakistan								Self-developed questionnaire					
**Thobani, 2023**[140]	Pakistan			PSQI								ESS		
**Toubasi, 2021**[141]	Jordan			PSQI					PSQI					
**Traore, 2023**[142]	Morocco			Spiegel scale					YIAT Questionnaire			ESS		
**Usman, 2017**[143]	Pakistan								NR					
**Varanasi, 2020**[144]	UAE			Self-developed questionnaire†					Self-developed questionnaire†			ESS		
**Vohra, 2019[145]**	Saudi Arabia			PSQI					PSQI					
**Waqas, 2015**[146]	Pakistan			PSQI					PSQI					
**Yassin, 2020**[147]	Jordan								Self-developed questionnaire					

**Zafar, 2020 a[148]**	Saudi Arabia											ESS		
**Zafar, 2020 b[149]**	Sudan			PSQI								ESS		
**Zainab, 2020[150]**	Pakistan											ESS		

Notes: ESS: Epworth Sleepiness Scale; CHERRIES: Checklist for Reporting Results of Internet E-Surveys; PSQI: Pittsburgh Sleep Quality Index; BCSQ-12-SS: Burnout; Clinical Subtypes Questionnaire; QoL: Quality of Life; SQS: Sleep Quality Scale; GSS: General Sleep Scale; GHQ-12: General Health Questionnaire; YIAT: Young's Internet Addiction Test; NR: Not Reported; M: Male; F: Female; CS: Cross-sectional study; †piloted and/or validated; *Insufficient data to compute prevalence/mean (excluded from the meta-analysis); $^{\Pi}$ Number of hours of sleep among high achievers before examination (excluded from the meta-analysis); $^{\phi}$ Not included in the mean score sub-group meta analyses (mean score assessed using a non-PSQI tool); $^{\Omega}$ Mean scores for gender and academic years were included according to the relevant sub-group meta-analysis.

Reported   Not Reported

Figure S1: Pooled prevalence of poor sleep quality in MENA medical students according to the instrument type

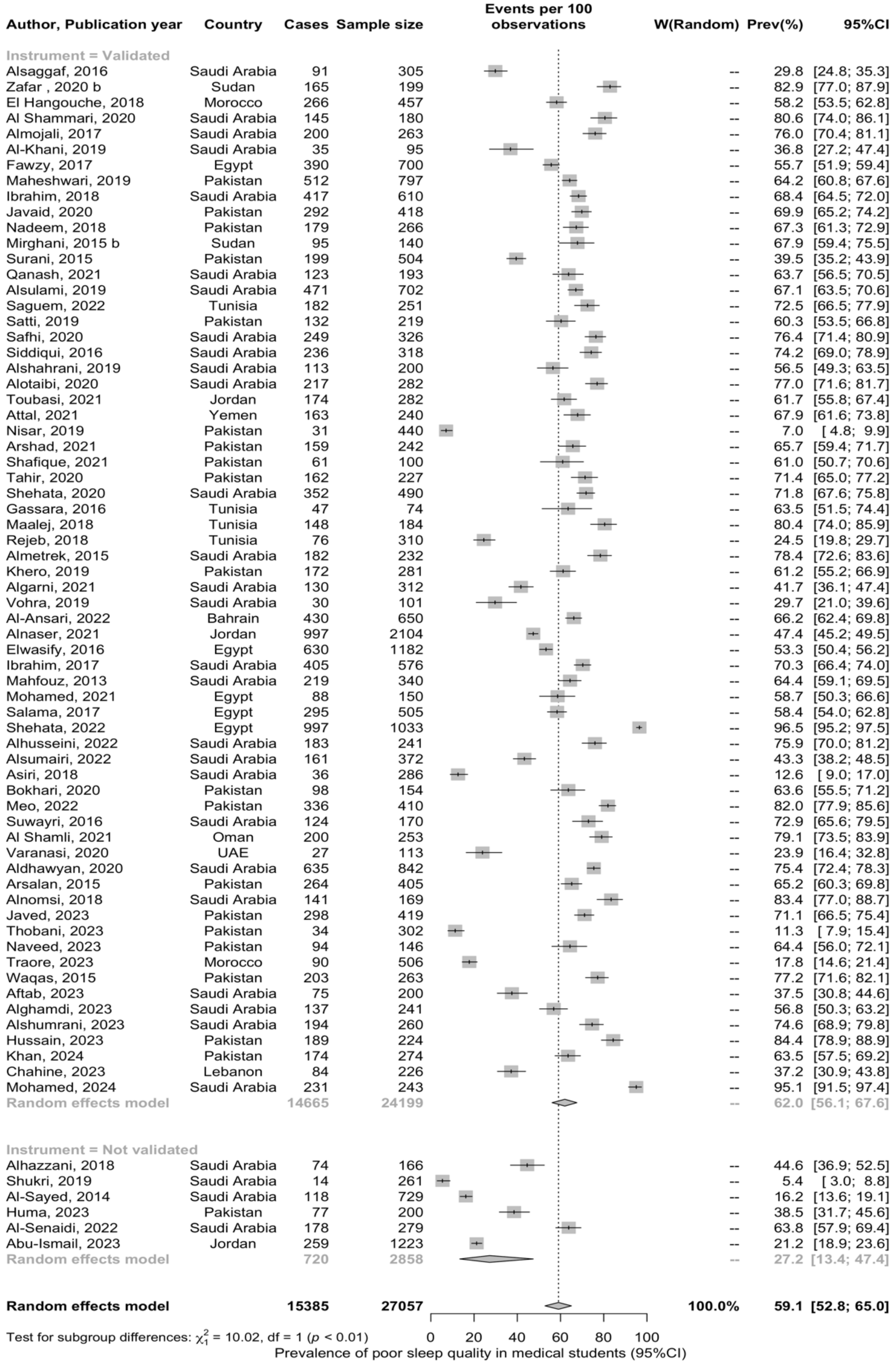

Note: UAE- United Arab Emirates.

Figure S2: Pooled Pittsburgh Sleep Quality Index (PSQI) mean scores for poor sleep quality in MENA medical students according to the instrument type

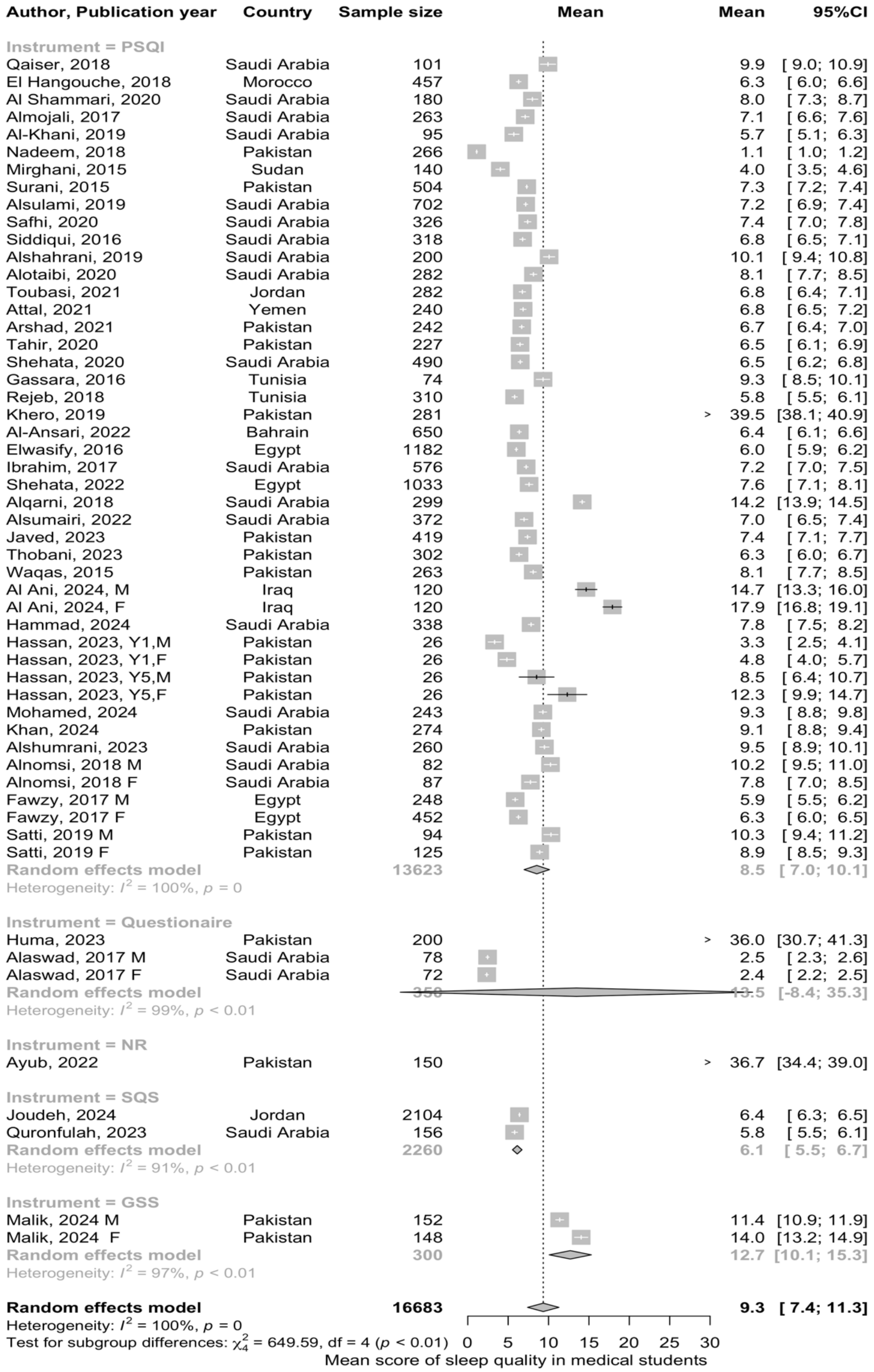

Note: PSQI- Pittsburgh Sleep Quality Index; SQS- Sleep Quality Scale; GSS- General Sleep Scale; NR- Not Reported; M- Male; F- Female.

Figure S3: Pooled prevalence of insufficient sleep duration in MENA medical students according to country classification

Author, Publication year	Country	Cases	Sample size	W(Random)	Prev(%)	95%CI
WBCountry = Lower Middle Income						
Khan, 2019	Pakistan	129	220	--	58.6	[51.8; 65.2]
Fawzy, 2017	Egypt	331	700	--	47.3	[43.5; 51.1]
Maheshwari, 2019	Pakistan	688	797	--	86.3	[83.7; 88.6]
Nadeem, 2018	Pakistan	183	266	--	68.8	[62.9; 74.3]
Naseer, 2019	Pakistan	40	137	--	29.2	[21.7; 37.6]
Sameer, 2020	Pakistan	285	441	--	64.6	[60.0; 69.1]
Saguem, 2022	Tunisia	39	251	--	15.5	[11.3; 20.6]
Nisar, 2019	Pakistan	256	440	--	58.2	[53.4; 62.8]
Shafique, 2021	Pakistan	57	100	--	57.0	[46.7; 66.9]
Tauseef, 2021	Pakistan	235	322	--	73.0	[67.8; 77.8]
Rejeb, 2018	Tunisia	120	310	--	38.7	[33.3; 44.4]
Rafiq, 2017	Pakistan	45	80	--	56.2	[44.7; 67.3]
Naeem, 2018	Pakistan	89	100	--	89.0	[81.2; 94.4]
Khero, 2019	Pakistan	193	281	--	68.7	[62.9; 74.1]
Gemnani, 2020	Pakistan	142	255	--	55.7	[49.4; 61.9]
Rafeeq 2021	Pakistan	204	295	--	69.2	[63.5; 74.4]
Talih, 2018	Lebanon	73	172	--	42.4	[35.0; 50.2]
Irshad, 2022	Pakistan	85	284	--	29.9	[24.7; 35.6]
Bhatti, 2012	Pakistan	31	60	--	51.7	[38.4; 64.8]
Khan, 2004	Pakistan	57	112	--	50.9	[41.3; 60.5]
Usman, 2017	Pakistan	239	270	--	88.5	[84.1; 92.1]
Javed, 2023	Pakistan	323	419	--	77.1	[72.8; 81.0]
Hashmi, 2022	Pakistan	27	60	--	45.0	[32.1; 58.4]
Gulzar, 2023	Pakistan	309	323	--	95.7	[92.8; 97.6]
Traore, 2023	Morocco	146	506	--	28.9	[24.9; 33.0]
Waqas, 2015	Pakistan	73	263	--	27.8	[22.4; 33.6]
Random effects model		4399	7464	--	58.9	[48.8; 68.3]
WBCountry = High Income						
Qaiser, 2018	Saudi Arabia	49	101	--	48.5	[38.4; 58.7]
Mansour, 2016	Saudi Arabia	40	122	--	32.8	[24.6; 41.9]
Al Zahrani, 2016	Saudi Arabia	79	161	--	49.1	[41.1; 57.1]
Almansour, 2020	Saudi Arabia	353	430	--	82.1	[78.1; 85.6]
Abdulghani, 2012	Saudi Arabia	249	491	--	50.7	[46.2; 55.2]
Safhi, 2020	Saudi Arabia	240	326	--	73.6	[68.5; 78.3]
Siddiqui, 2016	Saudi Arabia	212	318	--	66.7	[61.2; 71.8]
Albaker, 2021	Saudi Arabia	15	163	--	9.2	[5.2; 14.7]
Algarni, 2021	Saudi Arabia	155	312	--	49.7	[44.0; 55.4]
Algarni, 2019	Saudi Arabia	140	444	--	31.5	[27.2; 36.1]
Mansour, 2020	Saudi Arabia	198	279	--	71.0	[65.3; 76.2]
Ali, 2021	Saudi Arabia	128	206	--	62.1	[55.1; 68.8]
Abdulrahman, 2021	Saudi Arabia	475	675	--	70.4	[66.8; 73.8]
Vohra, 2019	Saudi Arabia	70	101	--	69.3	[59.3; 78.1]
AlQahtani, 2017	Saudi Arabia	186	237	--	78.5	[72.7; 83.5]
Albhlal, 2017	Saudi Arabia	59	128	--	46.1	[37.2; 55.1]
Alqarni, 2018	Saudi Arabia	215	299	--	71.9	[66.4; 76.9]
Amin, 2016	Saudi Arabia	279	341	--	81.8	[77.3; 85.8]
Shukri, 2019	Saudi Arabia	167	261	--	64.0	[57.8; 69.8]
Hamed, 2015	UAE	122	190	--	64.2	[57.0; 71.0]
Alzunidi, 2022	Saudi Arabia	70	99	--	70.7	[60.7; 79.4]
Varanasi, 2020	UAE	91	113	--	80.5	[72.0; 87.4]
Al-Sayed, 2014	Saudi Arabia	182	729	--	25.0	[21.9; 28.3]
Alkhaibary, 2017	Saudi Arabia	130	234	--	55.6	[48.9; 62.0]
Alrasheed, 2023	Saudi Arabia	140	279	--	50.2	[44.2; 56.2]
Al-Senaidi, 2022	Saudi Arabia	238	279	--	85.3	[80.6; 89.2]
Chaabna K, 2022	Qatar	25	41	--	61.0	[44.5; 75.8]
Mirza, 2021	Saudi Arabia	142	231	--	61.5	[54.9; 67.8]
Ibrahim, 2013	Saudi Arabia	353	597	--	59.1	[55.1; 63.1]
Al-bukhari, 2016	Saudi Arabia	412	555	--	74.2	[70.4; 77.8]
Almutairi, 2017	Saudi Arabia	347	511	--	67.9	[63.7; 71.9]
Al-Ghamdi, 2015	Saudi Arabia	114	167	--	68.3	[60.6; 75.2]
Alotaibi, 2023	Saudi Arabia	130	220	--	59.1	[52.3; 65.7]
Alshumrani, 2023	Saudi Arabia	224	260	--	86.2	[81.3; 90.1]
Mohamed, 2024	Saudi Arabia	128	243	--	52.7	[46.2; 59.1]
Random effects model		6157	10143	--	61.7	[55.2; 67.9]
WBCountry = Upper Middle Income						
Toubasi, 2021	Jordan	134	282	--	47.5	[41.6; 53.5]
Abdulah, 2018	Iraq	165	317	--	52.1	[46.4; 57.7]
Alqudah, 2022	Jordan	232	299	--	77.6	[72.4; 82.2]
Abu-Ismail, 2023	Jordan	990	1223	--	80.9	[78.6; 83.1]
Yassin, 2020	Jordan	602	1041	--	57.8	[54.8; 60.9]
AL-Khaliq, 2023	Iraq	139	250	--	55.6	[49.2; 61.9]
Random effects model		2262	3412	--	63.1	[51.6; 73.3]
WBCountry = Low Income						
Attal, 2021	Yemen	51	240	--	21.2	[16.3; 27.0]
Mohammed, 2020	Sudan	62	100	--	62.0	[51.7; 71.5]
Mirghani, 2015	Sudan	45	108	--	41.7	[32.3; 51.5]
Swed, 2023	Syria	1205	2891	--	41.7	[39.9; 43.5]
Ibrahim, 2023	Sudan	220	340	--	64.7	[59.4; 69.8]
Ibrahim, 2024	Sudan	220	340	--	64.7	[59.4; 69.8]
Random effects model		1803	4019	--	48.9	[35.3; 62.5]
**Random effects model**		**14621**	**25038**	**100.0%**	**59.8**	**[54.7; 64.6]**

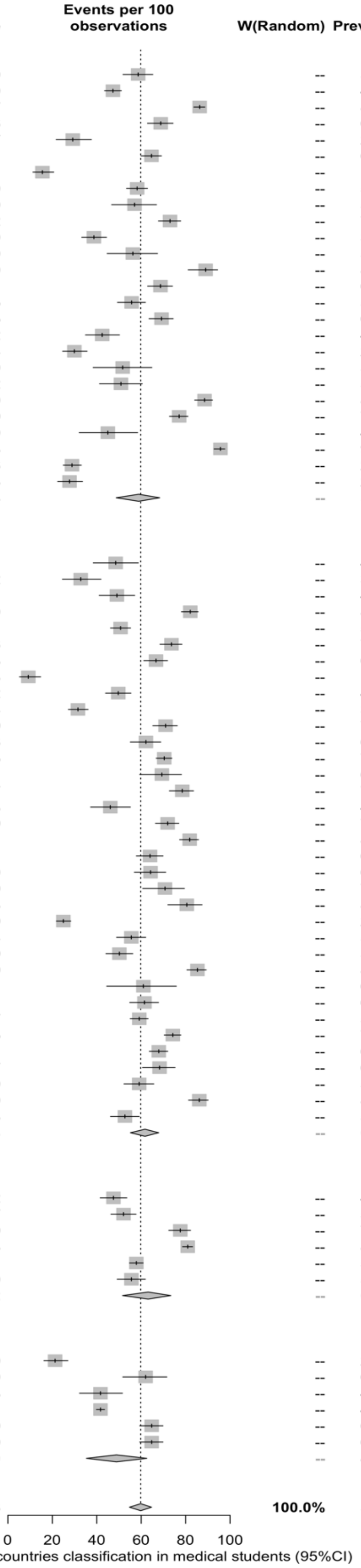

Test for subgroup differences: $\chi^2_3 = 3.13$, df = 3 ($p = 0.37$)

Note: UAE- United Arab Emirates.

Figure S4: Pooled mean scores of insufficient sleep duration in MENA medical students according to the instrument type

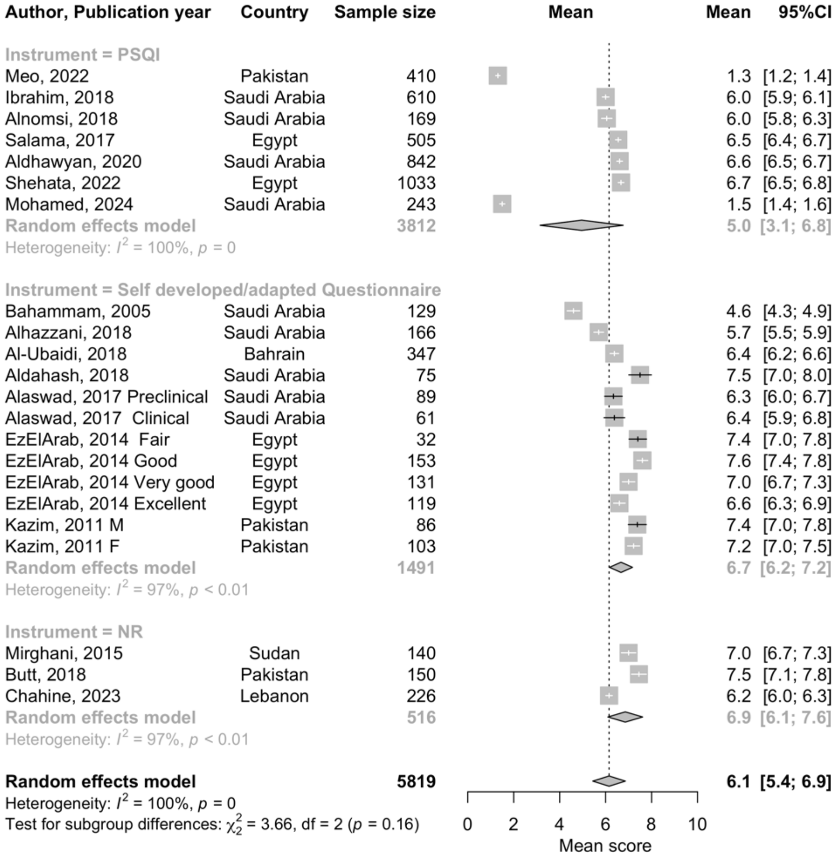

Note: PSQI- Pittsburgh Sleep Quality Index; NR- Not Reported; M- Male; F-Female.

Figure S5: Pooled prevalence of Excessive Daytime Sleepiness (EDS) in MENA medical students according to the instrument type

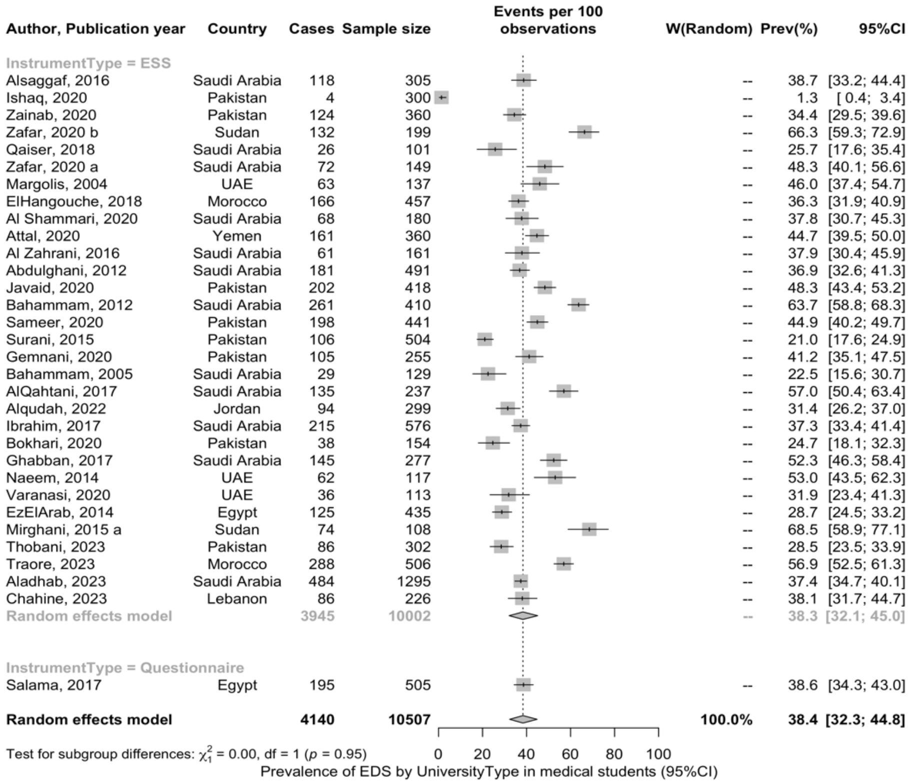

Note: ESS- Epworth Sleepiness Scale; UAE- United Arab Emirates.

Figure S6: Pooled Epworth Sleepiness Scale (ESS) mean scores for Excessive Daytime Sleepiness (EDS) in MENA medical students according to the instrument type

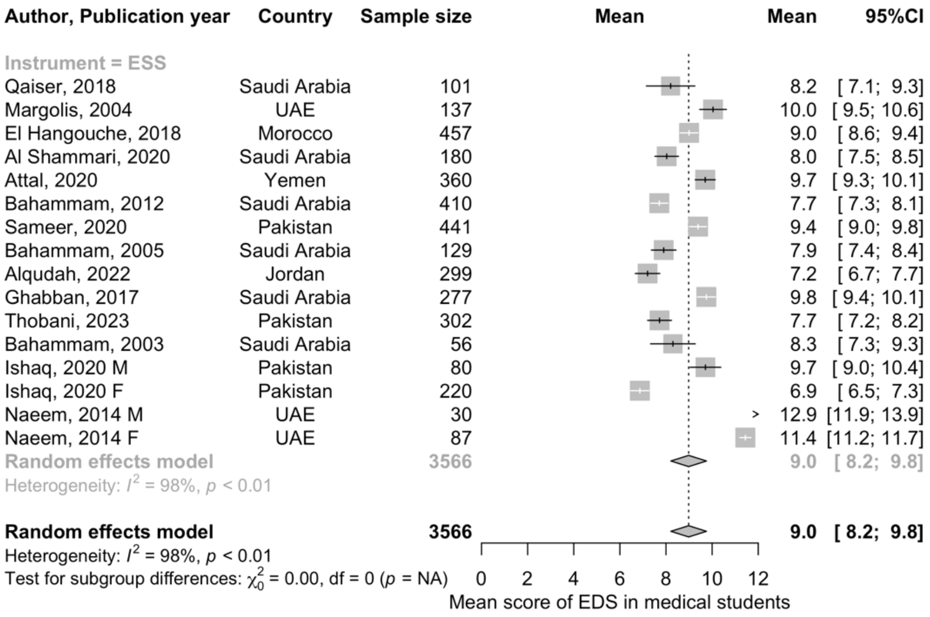

Note: ESS- Epworth Sleepiness Scale; UAE- United Arab Emirates; M- Male; F-Female.

Table S4: Meta-analysis of sleep disturbances prevalence among medical students in MENA countries

	Number of prevalence measures	Total sample size	Prevalence range (%)	Effect size		Subgroup Comparison (Q between subgroup tests p-value)		Heterogeneity between studies I^2 (%)
				Weighted average prevalence (%)	95% CI			
**Poor Sleep Quality**								
Countries classification (n=72)								
High-income	34	11000	5.4-95.1	59.2	49.7-68.0	**0.0187**		98.0
Upper-middle income	3	3609	21.2-61.7	42.1	23.8-62.8			99.3
Lower-middle income	32	11869	7.0-96.5	59.0	49.7-67.7			98.1
Low-income	3	579	67.9-82.9	73.5	64.2-81.1			86.2
Sex (n=106)								
M	36	5397	5.6-80.6	61.0	54.6-67.0	0.1622	**<0.0001**	92.7
F	31	6297	19.4-88.0	67.0	61.1-72.4			93.3
M/F	38	15802	5.4-96.5	57.6	47.5-67.1	N/A		98.6
N/R	1	286	12.6	12.6	9.0-17.0	N/A		N/A
Training period (n=89)								
Preclinical	22	5056	19.1-84.4	64.1	55.7-71.8	**<0.0001**	**<0.0001**	97.0
Clinical	25	5515	5.4-95.1	59.5	47.7-70.3			97.5
Late clinical years	1	226	37.2	37.2	30.9-43.8			N/A
Mixed training periods	27	14301	7.0-96.5	58.1	47.3-68.1	N/A		98.6
Unknown training period	14	4063	12.6-80.4	56.8	45.5-67.5	N/A		96.3
Data collection time (n=72)								
Before 2020	55	18996	5.4-83.4	57.4	50.7-63.8	0.5223		97.7
2020-2021	9	5420	17.8-82.0	58.0	41.2-73.1			99.1
After 2021	8	2641	37.2-96.5	71.3	47.3-87.3			98.7
Type of university (n=72)								
Public	58	23228	5.4-96.5	59.5	52.9-65.9	0.9123	0.1405	98.2
Private	9	2245	11.3-83.4	58.5	40.3-74.6			98.0
Mixed public and private	3	739	63.5-82.9	71.9	60.8-80.8	N/A		90.6
NR	2	845	7.0-65.2	27.4	3.9-77.9	N/A		99.6
Type of tool (n=72)								
Validated	66	24199	7.0-96.5	62.0	56.1-67.6	**0.0015**		97.7
Not Validated	6	2858	5.4-63.8	27.2	13.4-47.4			98.4
Academic performance (n= 22)								
Good academic performance	13	3213	10.7-96.6	65.7	49.8-78.7	0.6146		97.7
Poor academic performance	9	1224	15.0-96.0	72.4	48.5-87.9			92.6
Stress (n=14)								
Stressed	7	1243	40.6-92.3	79.5	67.2-88.1	**0.0058**		95.8
Not Stressed	7	617	13.8-69.8	53.5	39.2-67.3			85.0
Study period (n=72)								
Regular teaching period	67	23946	5.4-96.5	58.8	52.2-65.1	0.6832		98.2

During COVID-19 period with quarantine	5	3111	38.5-82.0	62.2	46.7-75.6			98.0
**Insufficient sleep duration**								
Countries (n=73)								
High-income	35	10143	9.2-86.2	61.7	55.2-67.9	0.3724		97.0
Upper-middle income	6	3412	47.5-80.9	63.1	51.6-73.3			97.9
Lower-middle income	26	7464	15.5-95.7	58.9	48.8-68.3			97.9
Low-income	6	4019	21.2-64.7	48.9	35.3-62.5			97.1
Sex (n=73)								
M/F combined	67	24146	9.2-95.7	60.1	54.6-65.3	0.4262		97.8
One gender study	6	892	46.1-69.3	56.3	48.7-63.7			81.8
Training period (n=73)								
Preclinical	7	1874	47.5-71.0	60.7	53.9-67.2	**0.0379**	0.1751	90.3
Clinical	5	912	21.2-64.0	44.3	31.2-58.2			95.6
Mixed training periods	46	16624	9.2-86.3	60.8	54.8-66.5	N/A		97.7
NR	15	5628	27.8-95.7	61.3	46.5-74.2	N/A		97.5
Data collection time (n=73)								
Before 2020	59	20378	9.2-89.0	59.0	53.9-63.8	0.8567		97.3
2020-2021	11	3857	15.5-95.7	64.5	44.8-80.3			98.9
After 2021	3	803	52.7-64.7	59.1	53.3-64.7			76.4
Type of university (n=73)								
Public	57	21335	9.2-95.7	60.1	54.1-65.8	0.2307	0.0596	97.9
Private	12	2216	27.8-77.1	53.5	44.4-62.4			95.8
Mixed public and private	3	1047	67.9-88.5	76.5	62.5-86.4	N/A		94.8
NR	1	440	58.2	58.2	53.4-62.8	N/A		N/A
Study period (n=74)								
Regular teaching period	72	24787	9.2-95.7	60.5	55.6-65.2	**<0.0001**		97.6
During COVID-19 period with quarantine	1	251	15.5	15.5	11.3-20.6			N/A
During Ramadan	1	220	63.6	63.6	56.9-70.0			N/A
**Excessive Daytime Sleepiness**								
Countries classification (n=32)								
High-income	15	4678	22.5-63.7	41.6	36.0-47.4	**0.0002**		92.0
Upper-middle income	1	299	31.4	31.4	26.2-37.0			NA
Lower-middle income	13	4863	1.3-56.9	30.7	20.7-42.9			95.4
Low-income	3	667	44.7-68.5	59.7	46.9-71.4			93.9
Sex (n=41)								
M	12	1636	3.8-48.9	27.3	20.6-35.2	0.3611		85.4
F	9	1575	0.5-58.1	37.7	18.8-61.3			83.8
M/F	20	7296	21-68.5	42.6	36.7-48.7	NA		95.3
Training period (n=32)								
Preclinical	3	1030	22.5-63.7	40.3	22.6-61.0	0.4271	0.5789	97.8
Clinical	4	953	37.8-68.5	47.0	35.4-59.0			90.2
Late clinical years	1	226	38.1	38.1	31.7-44.7			NA
Mixed training periods	20	7607	21.0-66.3	39.8	34.6-45.2	NA		94.3
NR	4	691	1.3-53.0	20.8	4.7-58.5	NA		95.7
Data collection time (n=32)								
Before 2020	29	8480	1.3-68.5	37.8	31.2-44.8	0.4667		94.2
2020-2021	2	1801	37.4-56.9	46.9	33.7-60.5			98.2

After 2021	1	226	38.1	38.1	31.7-44.7			NA
Type of university (n=32)								
Public	27	8051	21.0-68.5	40.8	36.2-45.5	**0.0042**	**0.0135**	93.5
Private	2	662	28.5-34.4	31.6	27.6-35.9			62.9
Mixed public and private	3	1794	1.3-66.3	20.3	2.2-74.2	NA		98.3
Study period (n=32)								
Regular teaching period	31	10147	1.3-68.5	38.1	31.9-44.8	0.1226		94.5
Assessment period	1	360	44.7	44.7	39.5-50.0			NA
Academic performance (n=5)								
Good academic performance	4	1343	29.0-63.7	40.3	27.7-34.9	0.1293		97.3
Poor academic performance	1	40	25.0	25.0	12.7-41.2			NA
Assessment tool (n=32)								
ESS	31	10002	1.3-68.5	38.3	32.1-45.0	0.9454		94.5
Questionnaire	1	505	38.6	38.6	34.3-43.0			NA

Notes: NR: Not Reported. N/A: Not applicable; PSQI = Pittsburgh Sleep Quality Index; ESS= Epworth Sleepiness Scale; M = Male; F = Female.
Weighted average prevalence measures were obtained using random-effect model. p-value ≤ 0.05 was considered statistically significant. Pooled prevalence measures for each subgroup includes only studies reporting the relevant stratified prevalence measures for the subgroup. The total number of prevalence measures in each outcome may then vary according to the subgroup analysis. Significant results are highlighted in bold (p-value<= 0.05). Preclinical year (1st and 2nd year); clinical years (3rd to 6th year). Before 2020- Likely to be before COVID-19 pandemic; 2020-2021 – Likely to be during COVID-19 pandemic with restrictions; After 2021- Likely to be after COVID-19 pandemic. MENA low-income countries (Sudan, Syria, and Yemen); lower-middle-income countries (Algeria, Djibouti, Egypt, Lebanon, Morocco, Pakistan, Palestine, and Tunisia), upper-middle-income countries (Iraq, Jordan, and Libya) and high-income countries (Bahrain, Kuwait, Oman, Qatar, Saudi Arabia, and United Arab Emirates (UAE)) according to the World Bank classification[151]. The stressed group includes students with moderate and high levels of stress as recommended by KPDS10 scale (moderate and high levels of stress require specialist referral). The good academic performance group includes students with grades of excellent, very good, good, >=70% on the percentage scale, 3-5 on a 5-point scale, or 3-4 on a 4-points scale. Poor academic performance includes Pass, Fail, <70% in the percentage scale, <3 on a 5-point scale or 4-points scale. Before 2020 refers to the period likely before the COVID-19 pandemic; 2020-2021likely during the COVID-19 pandemic; after 2021 likely the after COVID-19 pandemic.

Table S5: Meta-analysis of sleep disturbance mean scores and mean sleeping time in MENA medical students

	Number of mean measures	Total sample size	Mean range	Effect size		Subgroup Comparison (Q between subgroup tests p-value)		Heterogeneity between studies I^2 (%)
				Weighted Mean score/mean sleep duration	95% CI			
**Poor Sleep Quality**								
Countries classification (n=46)								
High-income	19	5864	5.7-14.2	8.2	7.3-9.1	0.35		99
Upper-middle income	3	522	6.8-17.9	13.1	6.6-19.6			100
Lower-middle income	24	7237	1.1-39.5	8.2	5.4-11.0			100
Low-income	0	-	-	-	-			-
Gender (n=53)								
M	13	1560	3.3-14.7	9.0	7.3-10.8	0.99	0.80	99
F	12	1972	4.8-17.9	9.0	6.8-11.2			99
M/F	28	10598	1.1-39.5	8.1	5.8-10.5	N/A		100
Training period (n=48)								
Preclinical	6	1543	3.3-8.1	6.0	4.7-7.4	**<0.01**	**0.02**	96
Clinical	13	2433	4.0-17.9	9.5	7.5-11.5			99
Mixed training periods	23	8820	5.7-39.5	8.9	6.1-11.7	N/A		100
Unknown	6	2227	1.1-10.1	6.9	4.3-9.4	N/A		100
Data collection time (n=46)								
Before 2020	35	11131	1.1-39.5	8.2	6.3-10.2	0.29		100
2020-2021	1	260	9.5	9.5	8.9-10.1			N/A
After 2021	6	2128	7.6-17.9	11.0	7.7-14.4			99
NR	4	104	3.3-12.3	7.1	3.2-11.0			95
Type of university (n=46)								
Public	38	11835	3.3-39.5	8.9	7.0-10.7	0.26	0.40	99
Private	6	1248	5.7-10.2	7.6	6.3-8.8			96
Mixed public and private	2	540	1.1-9.1	5.1	-2.7-13.0	N/A		100
Type of tool (n=54)								
PSQI	46	13623	1.1-39.5	8.5	7.0-10.1	**<0.01**		100
Self-developed questionnaire	3	350	2.4-36.0	13.5	-8.4-35.3			99
NR	1	150	36.7	36.7	34.4-39.0			NA
SQS	2	2260	5.8-6.4	6.1	5.5-6.7			91
GSS	2	300	11.4-14.0	12.7	10.1-15.3			97
Academic performance (n=12)								
Good academic performance	6	1423	4.0-17.5	10.7	6.4-14.9	0.15		100
Poor academic performance	6	498	10.6-20.2	14.4	11.7-17.1			98
Sampling category (n=46)								
Non-probability-based sampling	12	3061	6.4-14.7	8.7	7.4-10.0	0.31	0.54	98
Probability based sampling	17	4818	3.3-17.9	7.7	6.1-9.2			98

NR	17	5744	1.1-39.5	9.2	5.3-13.1	N/A		100
Sample size category (n=46)								
>100	37	13087	1.1-39.5	8.7	6.8-10.6	0.60		100
<=100	9	536	3.3-12.3	8.0	6.1-9.8			97
**Mean sleep duration (min)**								
Countries classification (n=22)								
High-income	10	2731	1.5-7.5	5.7	4.7-6.7	**0.05**		100
Upper-middle income	0	-	-	-	-			-
Lower-middle income	11	2948	1.3-7.5	6.5	5.4-7.5			100
Low-income	1	140	7.0	7.0	6.7-7.3			NA
Sex (n=22)								
M	4	456	4.6-7.5	6.3	4.9-7.7	0.19	**0.02**	98
F	1	103	7.2	7.2	7.0-7.5			NA
M/F combined	17	5260	1.3-7.6	6.1	5.2-6.9	N/A		100
Training period (n=22)								
Preclinical	4	1407	4.6-6.6	6.0	5.1-6.9	0.82	**<0.01**	98
Clinical	6	854	1.5-7.5	5.7	4.0-7.4			100
Late clinical period	1	226	6.2	6.2	6.0-6.3			NA
Mixed training periods	9	3143	1.3-7.6	6.3	5.0-7.6	N/A		100
Unknown	2	189	7.2-7.4	7.3	7.1-7.5	N/A		0
Data collection time (n=22)								
Before 2020	18	3907	4.6-7.6	6.6	6.3-7.0	**<0.01**		97
2020-2021	1	410	1.3	1.3	1.2-1.4			NA
After 2021	3	1502	1.5-6.7	4.8	1.6-8.0			100
Type of university (n=22)								
Public	17	4704	1.5-7.6	6.3	5.6-7.0	0.58	0.80	100
Private	4	768	1.3-7.4	5.5	2.7-8.3			100
Mixed public and private	1	347	6.4	6.4	6.2-6.6	N/A		NA
Assessment tool (n=22)								
PSQI	7	3812	1.3-6.7	5.0	3.1-6.8	0.16		100
Self-developed questionnaire/Adapted questionnaire	12	1491	4.6-7.5	6.7	6.2-7.2			97
NR	3	516	6.2-7.5	6.9	6.1-7.6			97
Academic performance (n=4)								
Good academic performance	3	403	6.6-7.6	7.1	6.5-7.6	0.34		94
Poor academic performance	1	32	7.4	7.4	7.0-7.8			NA
Sampling category (n=22)								
Probability based sampling	15	4000	1.3-7.6	6.2	5.4-7.0	0.81	0.47	100
Non-probability-based sampling	6	977	1.5-7.5	6.0	4.1-7.8			100
NR	1	842	6.6	6.6	6.5-6.7	N/A		N/A
Sample size category (n=22)								

>100	17	5476	1.3-7.6	5.9	5.0-6.8	**0.03**		100
<=100	5	343	6.3-7.5	7.0	6.5-7.5			87
Stress level (n=3)								
Stressed	2	197	6.0-6.1	6.1	6.0-6.3	0.38		0
Not Stressed	1	29	6.3	6.3	5.9-6.7			N/A
**Excessive Daytime Sleepiness**								
Countries classification (n=16)								
High-income	9	1407	7.7-12.9	9.4	8.2-10.5	**<0.01**		98
Upper-middle income	1	299	7.2	7.2	6.7-7.7			NA
Lower-middle income	5	1500	6.9-9.7	8.5	7.5-9.6			97
Low-income	1	360	9.7	9.7	9.3-10.1			NA
Sex (n=16)								
M	4	340	7.9-12.9	9.7	7.4-11.9	0.84	0.70	96
F	2	307	6.9-11.4	9.2	4.7-13.6			100
M/F	10	2919	7.2-10.0	8.7	8.1-9.3	N/A		94
Training period (n=16)								
Preclinical	2	539	7.7-7.9	7.8	7.5-8.1	0.20	**0.02**	0
Clinical	2	540	8.0-9.7	8.9	7.2-10.5			96
Mixed training periods	7	2014	7.2-10.0	8.8	8.0-9.6	N/A		94
Unknown	5	473	6.9-12.9	9.8	7.7-11.9	N/A		99
Data collection time (n=16)								
Before 2020	16	3566	6.9-12.9	9.0	8.2-9.8	NA		98
Type of university (n=16)								
Public	13	2964	7.2-12.9	9.2	8.3-10.1	**<0.01**	**0.02**	98
Private	1	302	7.7	7.7	7.2-8.2			NA
Mixed public and private	2	300	6.9-9.7	8.3	5.5-11.1			98
Study period (n=16)								
Regular teaching period	15	3206	6.9-12.9	8.9	8.1-9.8	0.11		98
Assessment period	1	360	9.7	9.7	9.3-10.1			NA
Assessment tool (n=16)								
ESS	16	3566	6.9-12.9	9.0	8.2-9.8	NA		98
Sampling category (n=16)								
Non-probability-based sampling	4	842	6.9-9.7	8.5	7.2-9.8	0.33	0.55	97
Probability based sampling	7	1653	7.7-12.9	9.5	8.1-11.0			99
NR	5	1071	7.2-10.0	8.6	7.5-9.7	N/A		96
Sample size category (n=16)								
>100	12	3313	6.9-10.0	8.5	7.8-9.1	**0.04**		96
<=100	4	253	8.3-12.9	10.6	8.7-12.5			96

Note: NR = Not Reported; NA = Not Applicable. GSS = General Sleep Scale; SQS = Sleep Quality Scale; M = Male; F = Female.
All mean score for poor sleep quality were measured using Pittsburgh Sleep Quality Index (PSQI). All mean score for EDS were measured using Epworth Sleepiness Scale (ESS). Significant results are highlighted in bold (p-value<= 0.05). Preclinical year include 1st and 2nd year; clinical years include 3rd to 6th year. MENA low-income countries include Sudan, Syria, and Yemen; lower-middle-income countries include Algeria, Djibouti, Egypt, Lebanon, Morocco, Pakistan, Palestine, and Tunisia; upper-middle-income countries include Iraq, Jordan, and Libya; and high-income countries include Bahrain, Kuwait, Oman, Qatar, Saudi Arabia, and United Arab Emirates (UAE) according to the World Bank classification[151]. The stressed group includes students with moderate and high levels of stress as recommended by KPDS10 scale (moderate and high levels of stress require specialist referral). The good academic performance group includes students with grades of excellent, very good, good, >=70% on the percentage scale, 3-5 on a 5-point scale, or 3-4 on a 4-points scale. Poor academic performance includes Pass, Fail, <70% in the percentage scale, <3 on a 5-point scale or 4-points scale. Before 2020 refers to the period likely before the COVID-19 pandemic; 2020-2021likely during the COVID-19 pandemic; after 2021 likely the after COVID-19 pandemic

Table S6: Quality assessment of the included studies

First author, year of publication	1. Was the study's target population a close representation of the national population in relation to relevant variables?	2. Was the sampling frame a true or close representation of the target population?	3. Was some form of random selection used to select the sample, OR was a census undertaken?	4. Was the likelihood of nonresponse bias minimal?	5. Were data collected directly from the subjects (as opposed to a proxy)?	6. Was an acceptable case definition used in the study?	7. Was the study instrument that measured the parameter of interest shown to have validity and reliability?			8. Was the same mode of data collection used for all subjects?	9. Was the length of the shortest prevalence period for the parameter of interest appropriate?	10. Were the numerator(s) and denominator(s) for the parameter of interest appropriate?
							7A.Quality	7B.Quantity	7C.EDS			
Abdulah, 2018[1]	High Risk	Low Risk	Low Risk	Low Risk	Low Risk	Low Risk	NA	Low Risk	NA	Low Risk	Low Risk	Low Risk
Abdulghani, 2012[2]	High Risk	Low Risk	High Risk	High Risk	Low Risk	Low Risk	NA	High Risk	Low Risk	Low Risk	Low Risk	Low Risk
Abdulrahman, 2021[3]	Low Risk	Low Risk	High Risk	Low Risk	Low Risk	Low Risk	NA	Low Risk	NA	Low Risk	Low Risk	Low Risk
Abu-Ismail, 2023[4]	High Risk	Low Risk	High Risk	High Risk	Low Risk	Low Risk	Low Risk	High Risk	NA	Low Risk	Low Risk	Low Risk
Aftab, 2023[5]	High Risk	High Risk	High Risk	Low Risk	Low Risk	Low Risk	Low Risk	NA	NA	Low Risk	Low Risk	Low Risk
Al Ani, 2024[6]	High Risk	Low Risk	High Risk	High Risk	Low Risk	Low Risk	Low Risk	NA	NA	Low Risk	Low Risk	High Risk
Al Shamli, 2021[7]	High Risk	Low Risk	Low Risk	High Risk	Low Risk	Low Risk	Low Risk	NA	NA	Low Risk	Low Risk	Low Risk
Al Shammari, 2020[8]	High Risk	Low Risk	Low Risk	High Risk	Low Risk	Low Risk	Low Risk	NA	Low Risk	Low Risk	Low Risk	Low Risk
Al Zahrani, 2016[9]	High Risk	High Risk	High Risk	High Risk	Low Risk	Low Risk	NA	High Risk	Low Risk	Low Risk	Low Risk	Low Risk
Al-Ansari, 2022[11]	High Risk	Low Risk	High Risk	High Risk	Low Risk	Low Risk	Low Risk	NA	NA	Low Risk	Low Risk	Low Risk
Al-Bukhari, 2016[15]	High Risk	Low Risk	High Risk	Low Risk	Low Risk	High Risk	NA	High Risk	NA	Low Risk	Low Risk	Low Risk

Al-Ghamdi, 2015[21]	High Risk	Low Risk	High Risk	Low Risk	Low Risk	Low Risk	NA	Low Risk	NA	Low Risk	Low Risk	Low Risk
Al-Kandari, 2017[26]	High Risk	Low Risk	High Risk	Low Risk	Low Risk	Low Risk	Low Risk	NA	NA	Low Risk	Low Risk	Low Risk
Al-Khaliq, 2023[28]	High Risk	Low Risk	Low Risk	High Risk	Low Risk	Low Risk	NA	High Risk	NA	Low Risk	Low Risk	Low Risk
Al-Khani, 2019[29]	High Risk	High Risk	High Risk	High Risk	Low Risk	Low Risk	Low Risk	NA	NA	Low Risk	Low Risk	Low Risk
Al-sayed, 2014[43]	High Risk	Low Risk	High Risk	Low Risk	Low Risk	Low Risk	High Risk	High Risk	NA	Low Risk	Low Risk	Low Risk
Al-senaidi, 2022[44]	High Risk	Low Risk	High Risk	High Risk	Low Risk	Low Risk	High Risk	High Risk	NA	Low Risk	Low Risk	Low Risk
Al-ubaidi, 2018[49]	Low Risk	Low Risk	Low Risk	High Risk	Low Risk	Low Risk	NA	High Risk	NA	Low Risk	Low Risk	Low Risk
Aladhab, 2023[10]	Low Risk	Low Risk	High Risk	High Risk	Low Risk	Low Risk	NA	NA	Low Risk	Low Risk	Low Risk	Low Risk
Alaswad, 2017[12]	High Risk	High Risk	Low Risk	Low Risk	Low Risk	Low Risk	Low Risk	Low Risk	NA	Low Risk	Low Risk	Low Risk
Albaker, 2021[13]	High Risk	Low Risk	Low Risk	High Risk	Low Risk	Low Risk	NA	Low Risk	NA	Low Risk	Low Risk	Low Risk
Albhlal, 2017[14]	High Risk	Low Risk	Low Risk	Low Risk	Low Risk	Low Risk	Low Risk	NA	NA	Low Risk	Low Risk	Low Risk
Aldahash, 2018[16]	High Risk	High Risk	Low Risk	High Risk	Low Risk	High Risk	NA	High Risk	NA	Low Risk	Low Risk	Low Risk
Aldhawyan, 2020[17]	High Risk	High Risk	High Risk	Low Risk	Low Risk	Low Risk	Low Risk	Low Risk	NA	Low Risk	Low Risk	Low Risk
Algarni, 2019[18]	High Risk	Low Risk	Low Risk	High Risk	Low Risk	Low Risk	NA	High Risk	NA	Low Risk	Low Risk	Low Risk
Algarni, 2021[19]	High Risk	Low Risk	Low Risk	High Risk	Low Risk	Low Risk	Low Risk	Low Risk	NA	Low Risk	Low Risk	Low Risk
Alghamdi, 2023[20]	High Risk	High Risk	High Risk	High Risk	Low Risk	Low Risk	Low Risk	NA	NA	Low Risk	Low Risk	Low Risk
Alhazzani, 2018[22]	High risk	Low Risk	Low Risk	High Risk	Low Risk	Low Risk	High Risk	High Risk	NA	Low Risk	Low Risk	Low Risk
Alhusseini, 2022[23]	High Risk	Low Risk	High Risk	High Risk	Low Risk	Low Risk	NA	Low Risk	NA	Low Risk	Low Risk	Low Risk
Ali, 2021[24]	High Risk	Low Risk	High Risk	High Risk	Low Risk	Low Risk	NA	High Risk	NA	Low Risk	Low Risk	Low Risk
Ali, 2023[25]	High Risk	Low Risk	Low Risk	High Risk	Low Risk	Low Risk	Low Risk	NA	NA	Low Risk	Low Risk	Low Risk
Alkhaibary, 2017[27]	Low Risk	Low Risk	Low Risk	High Risk	Low Risk	Low Risk	NA	High Risk	NA	Low Risk	Low Risk	Low Risk
Almansour, 2020[30]	High Risk	Low Risk	Low Risk	High Risk	Low Risk	Low Risk	NA	High Risk	NA	Low Risk	Low Risk	Low Risk
Almetrek, 2015[31]	High Risk	Low Risk	Low Risk	Low Risk	Low Risk	Low Risk	Low Risk	NA	NA	Low Risk	Low Risk	Low Risk
Almojali, 2017[32]	High Risk	Low Risk	Low Risk	Low Risk	Low Risk	Low Risk	Low Risk	NA	NA	Low Risk	Low Risk	Low Risk
Almutairi, 2017[33]	High Risk	Low Risk	Low Risk	High Risk	Low Risk	Low Risk	NA	Low Risk	NA	Low Risk	Low Risk	Low Risk
Alnaser, 2021[34]	Low Risk	Low Risk	High Risk	High Risk	Low Risk	Low Risk	Low Risk	NA	NA	Low Risk	Low Risk	Low Risk

Alnomsi, 2018[35]	High Risk	Low Risk	High Risk	High Risk	Low Risk	Low Risk	Low Risk	Low Risk	NA	Low Risk	Low Risk	Low Risk
Alotaibi, 2020[36]	High Risk	Low Risk	High Risk	High Risk	Low Risk	Low Risk	Low Risk	NA	NA	Low Risk	Low Risk	Low Risk
Alotaibi, 2023[37]	High Risk	High Risk	High Risk	High Risk	Low Risk	Low Risk	NA	Low Risk	NA	Low Risk	Low Risk	Low Risk
Alqahtani, 2017[38]	Low Risk	Low Risk	High Risk	Low Risk	Low Risk	Low Risk	Low Risk	NA	Low Risk	Low Risk	Low Risk	Low Risk
Alqarni, 2018[39]	High Risk	Low Risk	High Risk	High Risk	Low Risk	Low Risk	Low Risk	Low Risk	NA	Low Risk	Low Risk	Low Risk
Alqudah, 2022[40]	High Risk	Low Risk	High Risk	High Risk	Low Risk	Low Risk	NA	High Risk	Low Risk	Low Risk	Low Risk	Low Risk
Alrasheed, 2023 [41]	High Risk	Low Risk	Low Risk	Low Risk	Low Risk	Low Risk	NA	High Risk	NA	Low Risk	Low Risk	Low Risk
Alsaggaf, 2016[42]	High Risk	Low Risk	Low Risk	Low Risk	Low Risk	Low Risk	Low Risk	NA	Low Risk	Low Risk	Low Risk	Low Risk
Alshahrani, 2019[45]	High Risk	Low Risk	High Risk	Low Risk	Low Risk	Low Risk	Low Risk	NA	NA	Low Risk	Low Risk	Low Risk
Alshumrani, 2023[46]	High Risk	High Risk	High Risk	High Risk	Low Risk	Low Risk	Low Risk	Low Risk	NA	Low Risk	Low Risk	Low Risk
Alsulami, 2019[47]	Low Risk	Low Risk	High Risk	High Risk	Low Risk	Low Risk	Low Risk	NA	NA	Low Risk	Low Risk	Low Risk
Alsumairi, 2022[48]	High Risk	Low Risk	Low Risk	High Risk	Low Risk	Low Risk	Low Risk	NA	NA	Low Risk	Low Risk	Low Risk
Alzunidi, 2022[50]	High Risk	Low Risk	High Risk	High Risk	Low Risk	Low Risk	NA	Low Risk	NA	Low Risk	Low Risk	Low Risk
Amin, 2016[51]	High Risk	Low Risk	Low Risk	Low Risk	Low Risk	Low Risk	NA	Low Risk	NA	Low Risk	Low Risk	Low Risk
Arsalan, 2015[52]	High Risk	Low Risk	High Risk	High Risk	Low Risk	Low Risk	Low Risk	NA	NA	Low Risk	Low Risk	Low Risk
Arshad, 2021[53]	High Risk	Low Risk	High Risk	Low Risk	Low Risk	Low Risk	Low Risk	NA	NA	Low Risk	Low Risk	Low Risk
Asiri, 2018[54]	High Risk	Low Risk	Low Risk	High Risk	Low Risk	Low Risk	Low Risk	NA	NA	Low Risk	Low Risk	Low Risk
Attal, 2020[55]	High Risk	Low Risk	Low Risk	High Risk	Low Risk	Low Risk	NA	NA	Low Risk	Low Risk	Low Risk	Low Risk
Attal, 2021[56]	High risk	Low Risk	High Risk	Low Risk	Low Risk	Low Risk	Low Risk	Low Risk	NA	Low Risk	Low Risk	Low Risk
Ayub, 2022[57]	Low Risk	Low Risk	High Risk	Low Risk	Low Risk	High Risk	High Risk	NA	NA	Low Risk	Low Risk	Low Risk
Bahammam, 2003[58]	High Risk	Low Risk	High Risk	Low Risk	Low Risk	Low Risk	NA	NA	Low Risk	Low Risk	Low Risk	Low Risk
Bahammam, 2005[59]	High Risk	Low Risk	Low Risk	High Risk	Low Risk	Low Risk	NA	High Risk	Low Risk	Low Risk	Low Risk	Low Risk
Bahammam, 2012[60]	High Risk	Low Risk	Low Risk	Low Risk	Low Risk	Low Risk	NA	NA	Low Risk	Low Risk	Low Risk	Low Risk
Bhatti, 2012[61]	Low Risk	Low Risk	Low Risk	High Risk	Low Risk	Low Risk	NA	High Risk	NA	Low Risk	Low Risk	Low Risk
Bokhari, 2020[62]	High Risk	Low Risk	Low Risk	High Risk	Low Risk	Low Risk	Low Risk	NA	Low Risk	Low Risk	Low Risk	Low Risk
Butt, 2018[63]	High Risk	Low Risk	High Risk	High Risk	Low Risk	Low Risk	NA	High Risk	NA	Low Risk	Low Risk	Low Risk

Chaabna k, 2022[64]	High Risk	Low Risk	High Risk	Low Risk	Low Risk	Low Risk	NA	Low Risk	NA	Low Risk	Low Risk	Low Risk
Chahine, 2023[65]	High Risk	High Risk	High Risk	High Risk	Low Risk	Low Risk	Low Risk	High Risk	Low Risk	Low Risk	Low Risk	Low Risk
El hangouche, 2018[66]	High Risk	Low Risk	Low Risk	Low Risk	Low Risk	Low Risk	Low Risk	NA	Low Risk	Low Risk	Low Risk	Low Risk
Elwasify, 2016[67]	Low Risk	Low Risk	High Risk	High Risk	Low Risk	Low Risk	Low Risk	NA	NA	Low Risk	Low Risk	Low Risk
Ezelarab, 2014[68]	High Risk	Low Risk	Low Risk	Low Risk	Low Risk	Low Risk	NA	Low Risk	Low Risk	Low Risk	Low Risk	Low Risk
Fawzy, 2017[69]	High Risk	Low Risk	High Risk	Low Risk	Low Risk	Low Risk	Low Risk	Low Risk	NA	Low Risk	Low Risk	Low Risk
Gassara, 2016[70]	High Risk	Low Risk	High Risk	High Risk	Low Risk	Low Risk	Low Risk	NA	NA	Low Risk	Low Risk	Low Risk
Gemnani, 2020[71]	High Risk	Low Risk	Low Risk	High Risk	Low Risk	Low Risk	NA	High Risk	Low Risk	Low Risk	Low Risk	Low Risk
Ghabban, 2017[72]	High Risk	Low Risk	High Risk	Low Risk	Low Risk	Low Risk	NA	NA	Low Risk	Low Risk	Low Risk	Low Risk
Gulzar, 2023[73]	Low Risk	Low Risk	High Risk	High Risk	Low Risk	Low Risk	NA	High Risk	NA	Low Risk	Low Risk	Low Risk
Hamed, 2015[74]	High Risk	Low Risk	High Risk	Low Risk	Low Risk	Low Risk	NA	High Risk	NA	Low Risk	Low Risk	Low Risk
Hammad, 2024[75]	High Risk	High Risk	Low Risk	Low Risk	Low Risk	Low Risk	Low Risk	NA	NA	Low Risk	Low Risk	High Risk
Hashmi, 2022[76]	High Risk	High Risk	High Risk	High Risk	Low Risk	Low Risk	NA	High Risk	NA	Low Risk	Low Risk	Low Risk
Hassan, 2023[77]	High Risk	High Risk	Low Risk	Low Risk	Low Risk	Low Risk	Low Risk	NA	NA	Low Risk	Low Risk	High Risk
Huma, 2023[78]	High Risk	Low Risk	High Risk	High Risk	Low Risk	Low Risk	High Risk	Low Risk	NA	Low Risk	Low Risk	Low Risk
Hussain, 2023[79]	High Risk	High Risk	High Risk	Low Risk	Low Risk	Low Risk	Low Risk	NA	NA	Low Risk	Low Risk	Low Risk
Ibrahim, 2013[80]	High Risk	Low Risk	Low Risk	High Risk	Low Risk	Low Risk	NA	Low Risk	NA	Low Risk	Low Risk	Low Risk
Ibrahim, 2017[81]	High Risk	Low Risk	Low Risk	High Risk	Low Risk	Low Risk	Low Risk	NA	Low Risk	Low Risk	Low Risk	Low Risk
Ibrahim, 2018[82]	High Risk	Low Risk	Low Risk	High Risk	Low Risk	Low Risk	Low Risk	Low Risk	NA	Low Risk	Low Risk	Low Risk
Ibrahim, 2023[83]	High Risk	Low Risk	Low Risk	Low Risk	Low Risk	Low Risk	High Risk	High Risk	NA	Low Risk	Low Risk	Low Risk
Ibrahim, 2024[84]	High Risk	High Risk	Low Risk	Low Risk	Low Risk	Low Risk	NA	High Risk	NA	Low Risk	Low Risk	High Risk
Irshad, 2022[85]	High Risk	Low Risk	High Risk	High Risk	Low Risk	Low Risk	NA	Low Risk	NA	Low Risk	Low Risk	Low Risk
Ishaq, 2020[86]	Low Risk	Low Risk	High Risk	High Risk	Low Risk	Low Risk	NA	NA	Low Risk	Low Risk	Low Risk	Low Risk
Jahangeer, 2021[87]	High Risk	Low Risk	Low Risk	Low Risk	Low Risk	Low Risk	High Risk	NA	NA	Low Risk	Low Risk	Low Risk
Javaid, 2020[88]	High Risk	Low Risk	High Risk	High Risk	Low Risk	Low Risk	Low Risk	NA	Low Risk	Low Risk	Low Risk	Low Risk
Javed, 2023[89]	High Risk	Low Risk	Low Risk	Low Risk	Low Risk	Low Risk	Low Risk	Low Risk	NA	Low Risk	Low Risk	Low Risk

Joudeh, 2024[90]	Low Risk	Low Risk	Low Risk	High Risk	Low Risk	Low Risk	Low Risk	NA	NA	Low Risk	Low Risk	High Risk
Kazim, 2011[91]	High Risk	Low Risk	High Risk	Low Risk	Low Risk	Low Risk	NA	High Risk	NA	Low Risk	Low Risk	Low Risk
Khan, 2004[92]	High Risk	Low Risk	High Risk	High Risk	Low Risk	Low Risk	NA	High Risk	NA	Low Risk	Low Risk	Low Risk
Khan, 2019[93]	High Risk	Low Risk	High Risk	High Risk	Low Risk	Low Risk	NA	High Risk	NA	Low Risk	Low Risk	Low Risk
Khan, 2024[94]	Low Risk	High Risk	High Risk	Low Risk	Low Risk	Low Risk	Low Risk	NA	NA	Low Risk	Low Risk	Low Risk
Khero, 2019[95]	High Risk	Low Risk	High Risk	High Risk	Low Risk	Low Risk	Low Risk	Low Risk	NA	Low Risk	Low Risk	Low Risk
Maalej, 2018[96]	High Risk	Low Risk	High Risk	High Risk	Low Risk	Low Risk	Low Risk	NA	NA	Low Risk	Low Risk	Low Risk
Maheshwari, 2019[97]	High Risk	Low Risk	High Risk	High Risk	Low Risk	Low Risk	Low Risk	Low Risk	NA	Low Risk	Low Risk	Low Risk
Mahfouz, 2013[98]	High Risk	Low Risk	Low Risk	Low Risk	Low Risk	Low Risk	Low Risk	Low Risk	NA	Low Risk	Low Risk	Low Risk
Malik, 2024[99]	High Risk	High Risk	High Risk	Low Risk	Low Risk	High Risk	High Risk	NA	NA	Low Risk	Low Risk	NA
Mansour, 2016[100]	High Risk	Low Risk	High Risk	High Risk	Low Risk	Low Risk	NA	High Risk	NA	Low Risk	Low Risk	Low Risk
Mansour, 2020[101]	High Risk	Low Risk	High Risk	High Risk	Low Risk	Low Risk	NA	High Risk	NA	Low Risk	Low Risk	Low Risk
Margolis, 2004[102]	High Risk	High Risk	High Risk	Low Risk	Low Risk	Low Risk	NA	NA	Low Risk	Low Risk	Low Risk	Low Risk
Meo, 2022[103]	High Risk	Low Risk	Low Risk	High Risk	Low Risk	Low Risk	Low Risk	Low Risk	NA	Low Risk	Low Risk	Low Risk
Mirghani, 2015a[104]	High Risk	Low Risk	High Risk	High Risk	Low Risk	Low Risk	NA	Low Risk	Low Risk	Low Risk	Low Risk	Low Risk
Mirghani, 2015b[105]	High Risk	High Risk	High Risk	Low Risk	Low Risk	Low Risk	Low Risk	Low Risk	NA	Low Risk	Low Risk	Low Risk
Mirza, 2021[106]	High Risk	Low Risk	Low Risk	High Risk	Low Risk	Low Risk	NA	High Risk	NA	Low Risk	Low Risk	Low Risk
Moalla, 2016[107]	High Risk	Low Risk	High Risk	High Risk	Low Risk	Low Risk	NA	Low Risk	NA	Low Risk	Low Risk	Low Risk
Mohamed, 2021[108]	High Risk	Low Risk	Low Risk	High Risk	Low Risk	Low Risk	Low Risk	NA	NA	Low Risk	Low Risk	Low Risk
Mohamed, 2024[109]	High Risk	High Risk	High Risk	High Risk	Low Risk	Low Risk	Low Risk	Low Risk	NA	Low Risk	Low Risk	Low Risk
Mohammed, 2020[110]	High Risk	Low Risk	Low Risk	High Risk	Low Risk	Low Risk	NA	High Risk	NA	Low Risk	Low Risk	Low Risk
Nadeem, 2018[111]	Low Risk	Low Risk	High Risk	Low Risk	Low Risk	Low Risk	Low Risk	Low Risk	NA	Low Risk	Low Risk	Low Risk
Naeem, 2014[112]	High Risk	High Risk	Low Risk	High Risk	Low Risk	Low Risk	NA	NA	Low Risk	Low Risk	Low Risk	Low Risk
Naeem, 2018[113]	High Risk	Low Risk	High Risk	High Risk	Low Risk	Low Risk	NA	Low Risk	NA	Low Risk	Low Risk	Low Risk
Naseer, 2019[114]	Low Risk	Low Risk	High Risk	High Risk	Low Risk	Low Risk	NA	Low Risk	NA	Low Risk	Low Risk	Low Risk
Naveed, 2023[115]	High Risk	Low Risk	High Risk	High Risk	Low Risk	Low Risk	Low Risk	NA	NA	Low Risk	Low Risk	Low Risk

Nisar, 2019[116]	Low Risk	Low Risk	High Risk	High Risk	Low Risk	Low Risk	Low Risk	Low Risk	NA	Low Risk	Low Risk	Low Risk
Qaiser, 2018[117]	High Risk	Low Risk	High Risk	High Risk	Low Risk	Low Risk	Low Risk	Low Risk	Low Risk	Low Risk	Low Risk	Low Risk
Qanash, 2021[118]	Low Risk	Low Risk	High Risk	High Risk	Low Risk	Low Risk	Low Risk	NA	NA	Low Risk	Low Risk	Low Risk
Quronfulah, 2023[119]	High Risk	High Risk	High Risk	High Risk	Low Risk	High Risk	Low Risk	NA	NA	Low Risk	Low Risk	High Risk
Rafeeq 2021[120]	High Risk	Low Risk	High Risk	Low Risk	Low Risk	Low Risk	NA	High Risk	NA	Low Risk	Low Risk	Low Risk
Rafiq, 2017[121]	High Risk	Low Risk	High Risk	High Risk	Low Risk	Low Risk	Low Risk	NA	NA	Low Risk	Low Risk	Low Risk
Rejeb, 2018[122]	High Risk	Low Risk	High Risk	Low Risk	Low Risk	Low Risk	Low Risk	Low Risk	NA	Low Risk	Low Risk	Low Risk
Safhi, 2020[123]	High Risk	Low Risk	Low Risk	High Risk	Low Risk	Low Risk	Low Risk	Low Risk	NA	Low Risk	Low Risk	Low Risk
Saguem, 2022[124]	High Risk	Low Risk	High Risk	High Risk	Low Risk	Low Risk	Low Risk	Low Risk	NA	Low Risk	Low Risk	Low Risk
Saif, 2024[125]	High Risk	Low Risk	High Risk	Low Risk	Low Risk	High Risk	NA	Low Risk	NA	Low Risk	Low Risk	Low Risk
Salama, 2017[126]	High risk	Low Risk	Low Risk	Low Risk	Low Risk	Low Risk	Low Risk	Low Risk	High Risk	Low Risk	Low Risk	Low Risk
Sameer, 2020[127]	High Risk	Low Risk	High Risk	High Risk	Low Risk	Low Risk	NA	Low Risk	Low Risk	Low Risk	Low Risk	Low Risk
Satti, 2019[128]	High Risk	High Risk	High Risk	High Risk	Low Risk	Low Risk	Low Risk	NA	NA	Low Risk	Low Risk	Low Risk
Shafique, 2021[129]	High Risk	Low Risk	High Risk	High Risk	Low Risk	Low Risk	Low Risk	NA	NA	Low Risk	Low Risk	Low Risk
Shehata, 2020[130]	High Risk	Low Risk	High Risk	High Risk	Low Risk	Low Risk	Low Risk	NA	NA	Low Risk	Low Risk	Low Risk
Shehata, 2022[131]	High Risk	Low Risk	Low Risk	High Risk	Low Risk	Low Risk	Low Risk	Low Risk	NA	Low Risk	Low Risk	Low Risk
Shukri, 2019[132]	High Risk	Low Risk	Low Risk	Low Risk	Low Risk	Low Risk	Low Risk	Low Risk	NA	Low Risk	Low Risk	Low Risk
Siddiqui, 2016[133]	High Risk	High Risk	High Risk	Low Risk	Low Risk	Low Risk	Low Risk	Low Risk	NA	Low Risk	Low Risk	Low Risk
Surani, 2015[134]	Low Risk	Low Risk	High Risk	Low Risk	Low Risk	Low Risk	Low Risk	NA	Low Risk	Low Risk	Low Risk	Low Risk
Suwayri, 2016[135]	High Risk	Low Risk	Low Risk	High Risk	Low Risk	Low Risk	Low Risk	NA	NA	Low Risk	Low Risk	Low Risk
Swed, 2023[136]	Low Risk	Low Risk	High Risk	Low Risk	Low Risk	High Risk	NA	High Risk	NA	Low Risk	Low Risk	Low Risk
Tahir, 2020[137]	High Risk	Low Risk	Low Risk	High Risk	Low Risk	Low Risk	Low Risk	NA	NA	Low Risk	Low Risk	Low Risk
Talih, 2018[138]	High Risk	Low Risk	Low Risk	High Risk	Low Risk	Low Risk	NA	High Risk	NA	Low Risk	Low Risk	Low Risk
Tauseef, 2021[139]	High Risk	Low Risk	High Risk	Low Risk	Low Risk	Low Risk	NA	High Risk	NA	Low Risk	Low Risk	Low Risk
Thobani, 2023[140]	High Risk	Low Risk	High Risk	High Risk	Low Risk	Low Risk	Low Risk	NA	Low Risk	Low Risk	Low Risk	Low Risk
Toubasi, 2021[141]	High Risk	Low Risk	High Risk	High Risk	Low Risk	Low Risk	Low Risk	Low Risk	NA	Low Risk	Low Risk	Low Risk

Traore, 2023[142]	High Risk	Low Risk	High Risk	High Risk	Low Risk	Low Risk	Low Risk	Low Risk	Low Risk	Low Risk	Low Risk	Low Risk
Usman, 2017[143]	High Risk	Low Risk	High Risk	Low Risk	Low Risk	Low Risk	NA	High Risk	NA	Low Risk	Low Risk	Low Risk
Varanasi, 2020[144]	High Risk	Low Risk	Low Risk	High Risk	Low Risk	Low Risk	Low Risk	Low Risk	Low Risk	Low Risk	Low Risk	Low Risk
Vohra, 2019[145]	High Risk	High Risk	Low Risk	High Risk	Low Risk	Low Risk	Low Risk	Low Risk	NA	Low Risk	Low Risk	Low Risk
Waqas, 2015[146]	High Risk	Low Risk	Low Risk	Low Risk	Low Risk	Low Risk	Low Risk	Low Risk	NA	Low Risk	Low Risk	Low Risk
Yassin, 2020[147]	High Risk	Low Risk	Low Risk	High Risk	Low Risk	Low Risk	NA	High Risk	NA	Low Risk	Low Risk	Low Risk
Zafar, 2020 a[148]	High Risk	Low Risk	Low Risk	High Risk	Low Risk	Low Risk	Low Risk	NA	Low Risk	Low Risk	Low Risk	Low Risk
Zafar, 2020 b[149]	High Risk	Low Risk	Low Risk	High Risk	Low Risk	Low Risk	NA	NA	Low Risk	Low Risk	Low Risk	Low Risk
Zainab, 2020[150]	High Risk	Low Risk	High Risk	High Risk	Low Risk	Low Risk	NA	NA	Low Risk	Low Risk	Low Risk	Low Risk

Note: NA = Not Applicable. The methodological quality assessment of the included studies was assessed using the Risk of Bias (RoB) tool for prevalence studies[152].

High risk of bias   Low risk of bias   Not applicable

Reference list

1 Abdulah DM, Piro RS. Sleep disorders as primary and secondary factors in relation with daily functioning in medical students. Annals of Saudi medicine. 2018;38:57-64.

2 Abdulghani HM, Alrowais NA, Bin-Saad NS, Al-Subaie NM, Haji AM, Alhaqwi AI. Sleep disorder among medical students: relationship to their academic performance. Medical teacher. 2012;34:S37-S41.

3 Bin Abdulrahman KA, Khalaf AM, Bin Abbas FB, Alanezi OT. The lifestyle of Saudi medical students. International Journal of Environmental Research and Public Health. 2021;18:7869.

4 Abu-Ismail L, Abuawwad MT, Taha MJ, Khamees Aa, Abu Ismail DY, Sanwar M, et al. Prevalence of Dry Eye Disease Among Medical Students and Its Association with Sleep Habits, Use of Electronic Devices and Caffeine Consumption: A Cross-Sectional Questionnaire. Clinical Ophthalmology. 2023:1013-23.

5 Aftab T, Khyzer E. Smartphone addiction and its association with hypertension and quality of sleep among medical students of Northern Border University, Arar, Saudi Arabia. Saudi Medical Journal. 2023;44:1013.

6 Al Ani HM, Al Shawi AF, Lafta RK, Abdulqadir O, Nadhim S, Abdulkarim S. Influence of stress, anxiety, and depression on sleep quality and academic performance of medical students in Fallujah University, Iraq. International Journal of Social Psychiatry. 2024:00207640241229381.

7 Al Shamli S, Al Omrani S, Al-Mahrouqi T, Chan MF, Al Salmi O, Al-Saadoon M, et al. Perceived stress and its correlates among medical trainees in Oman: a single-institution study. Taiwanese Journal of Psychiatry. 2021;35:188.

8 Al Shammari MA, Al Amer NA, Al Mulhim SN, Al Mohammedsaleh HN, AlOmar RS. The quality of sleep and daytime sleepiness and their association with academic achievement of medical students in the eastern province of Saudi Arabia. Journal of Family & Community Medicine. 2020;27:97.

9 Al-Zahrani JM, Aldossari KK, Abdulmajeed I, Al-Ghamdi SH, Al-Shamrani AM, Al-Qahtani NS. Daytime sleepiness and academic performance among medical students. Health Science Journal. 2016;10:1.

10 Aladhab W, Adnan S, Alenezi AM, Al Aithan A, Alkhlaif Z, Alsuhaymi A, et al. The association of excessive daytime sleepiness with psychological distress among Saudi medical students.

11 Al-Ansari AM, Jahrami HA, Hamadeh RR, Sater MS, Janahi AI, Janahi AK, et al. An Evaluation of the Prevalence of the Risk of Disordered Eating, Poor Sleep Quality and Perceived Stress in Medical Students. 2022.

12 Alaswad W, Alsuhibani R, Sharaf F. Differene of Sleeping Patterns and Habit between First and Third Year Medical Students. Internatonal Journal of Medical and Health Research. 2017;3:16-21.

13 Abdulmalik B Albaker SMA, Alhanoof S Almuhideb, Ohoud A Alobaid, Fai M Alanazi, Taghreed O alfadhel, Bushra S Alsakran. Impact of Video Games on Physical and Psychological Wellbeing Among Medical Students. J Res Med Dent Sci. 2021;9 327-33.

14 Albhlal L, Alanzi F, Ghannam K, Alqahtani A, Alenazi A, Alanazi M. Sleep disturbance patterns among medical students, Saudi Arabia. Arch Med. 2017;9.

15 Al-bukhari I, Al-Malki K, Kashkari M, Alrifai A, Adnan M. Prevalence and factors affecting irritable bowel syndrome among medical students at Taibah university. Clinical Medicine Research. 2016;5:1-5.

16 Aldahash FD, Alasmari SA, Alnomsi SJ, Alshehri AM, Alharthi NF, Aloufi AAH, et al. Relationship of body mass index to sleep duration, and current smoking among medical students in Tabuk City, Saudi Arabia. Electronic physician. 2018;10:7273.

17 Aldhawyan AF, Alfaraj AA, Elyahia SA, Alshehri SZ, Alghamdi AA. Determinants of subjective poor sleep quality in social media users among freshman college students. Nature and Science of Sleep. 2020:279-88.

18 Algarni ASA, Alqahtani WSS, Alotaibi FSA, Asiri MAM, Al Hoban MAM, Alshehri ZKS, et al. Gender Differences in Habits for a Healthy Lifestyle among Medical Students, Saudi Arabia. INDO AMERICAN JOURNAL OF PHARMACEUTICAL SCIENCES. 2019;6:636-42.

19 Algarni SA, Aljohani AS. Effect of smartphone addiction on sleep quality among medical students at Taibah University, Medina, Saudi Arabia. Medical Science. 2021;25:3266-78.

20 Alghamdi IK, Alrefai AM, Alghamdi TA, Nawawi AT, Badawy YA, Alghamdi I, et al. Prevalence of Contributing Factors Leading to the Development of Insulin Resistance Among Male Medical Students at a Private College in Saudi Arabia. Cureus. 2023;15.

21 Sameer AG, Faisal A, Abdullah A, Abdulaziz A, Abdulrahman T, Abdulrahman A, et al. A study of impact and prevalence of irritable bowel syndrome among medical students. International Journal of Medicine and Medical Sciences. 2015;7:139-47.

22 Alhazzani N, Masudi E, Algarni A, Alaklabi SM, Alomari AS, Alghamdi RN. The relationship between sleep patterns and academic performance among medical students at King Saud Bin Abdulaziz University for Health Sciences. The Egyptian Journal of Hospital Medicine. 2018;70:1131-4.

23 Alhusseini NK, Ramadan M, Almasry Y, Atout M, Hamsho K, Mahmoud M, et al. Effects of Sleep Quality on Academic Performance and Psychological Distress Among Medical Students in Saudi Arabia. Health Scope. 2022;11.

24 Ali A, Albahrani A, Alnasser A, Alsalman A, Alaithan M, Alswaidan M, et al. The Effect of caffeine on sleep among medical students at King Faisal University Saudi Arabia. Middle East Journal of Family Medicine. 2021;7:51.

25 Ali RM, Zolezzi M, Awaisu A, Eltorki Y. Sleep Quality and Sleep Hygiene Behaviours Among University Students in Qatar. International Journal of General Medicine. 2023:2427-39.

26 Al-Kandari S, Alsalem A, Al-Mutairi S, Al-Lumai D, Dawoud A, Moussa M. Association between sleep hygiene awareness and practice with sleep quality among Kuwait University students. Sleep health. 2017;3:342-7.
27 Alkhaibary A, Alghanim F, Najdi A, Bajaber M, Alakeel A, Aljabir M, et al. Effect of Video Games on Medical Students' Academic Performance: A Two-Institutions, Cross-Sectional Study. Age. 2017;19:21.
28 Abd AL-Khaliq IM. Prevalence of Irritable Bowel Syndrome and its Association with Anxiety among Students of AL-Kindy College of Medicine. South Asian Res J Bio Appl Biosci. 2023;5:60-7.
29 Al-Khani AM, Sarhandi MI, Zaghloul MS, Ewid M, Saquib N. A cross-sectional survey on sleep quality, mental health, and academic performance among medical students in Saudi Arabia. BMC research notes. 2019;12:1-5.
30 Almansour A, AlJammaz F, Ahmeda A, Alfawaz M, Abdulsalam K, AlSheikh A, et al. The Prevalence of Sleep Deprivation and its influence on Students' Life Attending Medical School at King Saud University. Int J Pharm Phytopharm Res. 2020;10:149-56.
31 Almetrek M, Alqahtani M, Alsamghan A, Alqahtani H, Alshahrani M, Alshahrani S, et al. Sleep quality among male medical students in King Khalid University. Journal of the Neurological Sciences. 2015;357:e173.
32 Almojali AI, Almalki SA, Alothman AS, Masuadi EM, Alaqeel MK. The prevalence and association of stress with sleep quality among medical students. Journal of epidemiology and global health. 2017;7:169-74.
33 Almutairi M, AlQazlan M, Alshebromi A, Alawad M, Zafar M. Prevalence of irritable bowel syndrome and its associated factors among medical students. Int J Med Res Health Sci. 2017;6:1-10.
34 Alnaser AR, Joudeh RM, Zitoun OA, Battah A, Al-Odat I, Jum'ah M, et al. The impact of COVID-19 pandemic on medical students' mental health and sleep quality in Jordan: a nationwide cross-sectional study. Middle East Current Psychiatry. 2021;28:1-10.
35 Shelian Juweed Alnomsi KSA, Omar Yarub Alali , Waled Mohammed Albalawi, Khalid Mohammed Albalawi, Wedyan Saleh Albalawi, Hyder Osman Mirghani. The Chronotype (Eveningness-Morningness) Effects on Academic Achievement among Medical Students in Tabuk City, Saudi Arabia. The Egyptian Journal of Hospital Medicine. 2018;71(7):3504-7.
36 Alotaibi AD, Alosaimi FM, Alajlan AA, Abdulrahman KAB. The relationship between sleep quality, stress, and academic performance among medical students. Journal of family & community medicine. 2020;27:23.
37 Alotaibi MI, Elsamad G, Aljardahi AN, Alghamdi AN, Alotaibi AI, Alorabi HM, et al. Changes in dietary and lifestyle behaviors and mental stress among medical students upon Ramadan diurnal intermittent fasting: a prospective cohort study from Taif/Saudi Arabia. BMC Public Health. 2023;23:1462.
38 AlQahtani MS, Alkhaldi TM, Al-Sultan AM, Bin Shihah AS, Aleid AS, Alzahrani ZK, et al. Sleeping disorders among medical students in Saudi Arabia; in relation to anti-insomnia medications. The Egyptian Journal of Hospital Medicine. 2017;69:2750-3.
39 Alqarni AB, Alzahrani NJ, Alsofyani MA, Almalki AA. The interaction between sleep quality and academic performance among the medical students in Taif university. The Egyptian Journal of Hospital Medicine. 2018;70:2202-8.
40 Alqudah M, Balousha SA, Balusha AA, Al-U'datt DaG, Saadeh R, Alrabadi N, et al. Daytime sleepiness among medical colleges' students in Jordan: Impact on academic performance. Sleep Disorders. 2022;2022.
41 Alrasheed T, Alwakeel A, Alarki M, Alkonani A, Albalawi R, Shaman M, et al. Cardiovascular disease risk factors among medical students at Tabuk University, Saudi Arabia during COVID-19 quarantine. Medical Science. 2023;27:e108ms2902.
42 Alsaggaf MA, Wali SO, Merdad RA, Merdad LA. Sleep quantity, quality, and insomnia symptoms of medical students during clinical years: relationship with stress and academic performance. Saudi medical journal. 2016;37:173.
43 Al-Sayed AA, Al-Rashoudi AH, Al-Eisa AA, Addar AM, Al-Hargan AH, Al-Jerian AA, et al. Sedative drug use among King Saud University medical students: a cross-sectional sampling study. Depression research and treatment. 2014;2014.
44 Al-Senaidi YI, Alanazi RM, Alsarhan LK, Alhomood LS, Alshalan LZ, Aldrebi WAA, et al. Sleep patterns and predictors of disturbed sleep among medical students at Imam Mohammad Ibn Saud Islamic University in Riyadh, Saudi Arabia, in 2022. MIDDLE EAST JOURNAL OF FAMILY MEDICINE.7:58.
45 Alshahrani M, Al Turki Y. Sleep hygiene awareness: Its relation to sleep quality among medical students in King Saud University, Riyadh, Saudi Arabia. Journal of family medicine and primary care. 2019;8:2628.
46 Alshumrani R, Shalabi B, Sultan A, Wazira L, Almutiri S, Sharkar A. Consumption of energy drinks and their effects on sleep quality among medical students. Journal of Family Medicine and Primary Care. 2023;12:1609-14.
47 Alsulami A, Bakhsh D, Baik M, Merdad M, Aboalfaraj N. Assessment of sleep quality and its relationship to social media use among medical students. Medical Science Educator. 2019;29:157-61.
48 Alsumairi NA, Alwagdani HA, Aloufi AO. Impact of smartphone overuse on sleep quality among medical students in Taif, Saudi Arabia. International Journal of Medicine in Developing Countries. 2022;6:979-91.
49 Al Ubaidi BA, Jassim G, Salem A. Burnout syndrome in medical students in the Kingdom of Bahrain. Global Journal of Health Science. 2018;10:86.
50 Alzunidi MA, Alowayyid JA, Benhadi RA, Farouk HH, Alshammari MH, Sultan AS, et al. Sleep Patterns and Academic Performance among Medicine and Pharm D students in Almaarefa University 0-0. Middle East Journal of Family Medicine. 2022;7:0.

51 Amin HS, Almazroua IS, Alsahlan AS, Alrishan MA, Elmourad HM, Alotaibi MM, et al. Effect of sleep deprivation on the attitude and performance of medical students, Riyadh, Saudi Arabia. Int J Med Sci Public Health. 2016;5:575-80.
52 Arsalan B, Ahmed F. Sleep hygiene and quality among medical Students. IJR. 2015;2:487-90.
53 Arshad D, Joyia UM, Fatima S, Khalid N, Rishi AI, Rahim NUA, et al. The adverse impact of excessive smartphone screen-time on sleep quality among young adults: A prospective cohort. Sleep Science. 2021;14:337.
54 Asiri AK, Almetrek MA, Alsamghan AS, Mustafa O, Alshehri SF. Impact of Twitter and WhatsApp on sleep quality among medical students in King Khalid University, Saudi Arabia. Sleep and Hypnosis (Online). 2018;20:247-52.
55 Attal BA, Al-Ammar FK, Bezdan M. Validation of the Arabic version of the Epworth sleepiness scale among the Yemeni medical students. Sleep disorders. 2020;2020.
56 Attal BA, Bezdan M, Abdulqader A. Quality of sleep and its correlates among Yemeni medical students: a cross-sectional study. Sleep disorders. 2021;2021.
57 Ayub S, Zafar MS, Kiran R, Asif A. Frustration intolerance, self-efficacy and sleep quality in medical students during pandemic of covid-19. Journal of Pakistan Psychiatric Society. 2022;19.
58 BaHammam A. Sleep pattern, daytime sleepiness, and eating habits during the month of Ramadan. Sleep and Hypnosis. 2003;5:165-74.
59 Bahammam AS, Al-Khairy OK, Al-Taweel AA. Sleep habits and patterns among medical students. Neurosciences Journal. 2005;10:159-62.
60 BaHammam AS, Alaseem AM, Alzakri AA, Almeneessier AS, Sharif MM. The relationship between sleep and wake habits and academic performance in medical students: a cross-sectional study. BMC medical education. 2012;12:1-6.
61 Bhatti AA, Khan UA, Khan HF. Sleep habits of first year and final year medical student. Rawal Med J. 2012;37:148-51.
62 Bokhari NM, Zafar M. Daytime sleepiness and sleep quality among undergraduate Medical students in Sialkot, Pakistan. Dr Sulaiman Al Habib Medical Journal [Internet]. 2020;2:51-5.
63 Butt AH, Bashir MM, Hassan U. CROSS SECTIONAL STUDY ON THE QUALITY AND PATTERNS OF SLEEP IN RELATION TO CONSUMPTION OF ENERGY DRINKS AMONG STUDENTS. INDO AMERICAN JOURNAL OF PHARMACEUTICAL SCIENCES. 2018;5:7741-5.
64 Chaabna K, Mamtani R, Abraham A, Maisonneuve P, Lowenfels AB, Cheema S. Physical activity and its barriers and facilitators among university students in Qatar: a cross-sectional study. International Journal of Environmental Research and Public Health. 2022;19:7369.
65 Chahine S, Kassem J, Wanna S, Almawy E, Matar H, Salameh P. Assessment of Sleep Among Lebanese University Medical Trainees During Clinical Years and Association With Shifts, Psychological Stress, and Fatigue. The Primary Care Companion for CNS Disorders. 2023;25:48938.
66 Hangouche AJE, Jniene A, Aboudrar S, Errguig L, Rkain H, Cherti M, et al. Relationship between poor quality sleep, excessive daytime sleepiness and low academic performance in medical students. Advances in medical education and practice. 2018:631-8.
67 Elwasify M, Barakat DH, Fawzy M, Elwasify M, Rashed I, Radwan DN. Quality of sleep in a sample of Egyptian medical students. Middle East Current Psychiatry. 2016;23:200-7.
68 Ez H, Rabie M, Ali D. Sleep behavior and sleep problems among a medical student sample in relation to academic performance: A cross-sectional questionnaire-based study. Middle East Current Psychiatry. 2014;21:72-80.
69 Fawzy M, Hamed SA. Prevalence of psychological stress, depression and anxiety among medical students in Egypt. Psychiatry research. 2017;255:186-94.
70 Gassara I, Ennaoui R, Halwani N, Turki M, Aloulou J, Amami O. Sleep quality among medical students. European Psychiatry. 2016;33:S594.
71 Gemnani VK, Shaikh AN, Mangrio RH, Tunio MI, Abbasi SA, Malik A. Prevalence of daytime sleepiness and its impact on academic performane amongst the university students. Rawal Medical Journal. 2020;45:959-.
72 Ghabban AI, Almouwalled MN, Al Orabi SA, Almutairi NS, Althagafi HM, Alqhamdi FS, et al. Excessive Daytime Sleepiness and Its Risk Factors Among Medical Students, Medical College, University of Tabuk, Kingdom of Saudi Arabia. Age.19:21.78.
73 Gulzar A, Javed A, Liaquat A, Javed D, Zahid M, Mumtaz H. Lifestyle and dietary habits change before and during quarantine and subsequent weight gain. Journal of Community Hospital Internal Medicine Perspectives. 2023;13:5.
74 Hamed H, Miskey A, Alkurd R, Ghazal Z, Sami R, Abduljaleel N, et al. The effect of sleeping pattern on the academic performance of undergraduate medical students at Ajman University of Science and Technology. Journal of Pharmacy and Pharmaceutical Sciences. 2015;4:18-21.
75 Hammad MA, Alyami MHF, Awed HS. The association between internet addiction and sleep quality among medical students in Saudi Arabia. Annals of Medicine. 2024;56:2307502.
76 Hashmi MRUI, Shoaib S, Sajjad SS, Amer A, Minhas SA, Amer A, et al. Effects of Diet and Physical Activity on BMI of Medical Students: Cross Sectional Study. Pakistan Journal of Medical & Health Sciences. 2022;16:125-.
77 Hassan H, Muzammil M, Malik MT, Karim MA, Lodhi MA, Jamil U. RELATIONSHIP OF SLEEP QUALITY WITH MENTAL WELL-BEING AND ACADEMIC PRODUCTIVITY. Pakistan Journal of Physiology. 2023;19:15-9.
78 Huma S, Qasim MF, Kamal MA, Khan FA, Naveen N. Frustration Intolerance, Self-Efficacy and Sleep Quality in Medical Students During Pandemic of Covid-19. Pakistan Journal of Medical & Health Sciences. 2023;17:604-.

79 Hussain M, Altaf M, Nadeem A, Aziz N, Waryam F. SLEEP QUALITY AND SLEEP PARALYSIS IN PAKISTANI UNDERGRADUATE MEDICAL STUDENTS. Journal of Population Therapeutics and Clinical Pharmacology. 2023;30.

80 Ibrahim NKR, Battarjee WF, Almehmadi SA. Prevalence and predictors of irritable bowel syndrome among medical students and interns in King Abdulaziz University, Jeddah. Libyan Journal of Medicine. 2013;8.

81 Ibrahim N, Badawi F, Mansouri Y, Ainousa A, Jambi S, Fatani A. Sleep quality among medical students at King Abdulaziz University: a cross-sectional study. J Community Med Health Educ. 2017;7:2161-711.

82 Ibrahim NK, Baharoon BS, Banjar WF, Jar AA, Ashor RM, Aman AA, et al. Mobile phone addiction and its relationship to sleep quality and academic achievement of medical students at King Abdulaziz University, Jeddah, Saudi Arabia. Journal of research in health sciences. 2018;18:e00420.

83 Ibrahim D, Ahmed RM, Bashir M, Mohammad AZ, Ibrahim B, Mohammed T, et al. How generalized anxiety disorder and perceived stress affect the lives of medical students in Sudan? 2023.

84 Ibrahim D, Ahmed RM, Mohammad AZ, Ibrahim B, Mohammed T, Mohamed ME, et al. Prevalence and correlates of generalized anxiety disorder and perceived stress among Sudanese medical students. BMC psychiatry. 2024;24:68.

85 Irshad K, Ashraf I, Azam F, Shaheen A. Burnout prevalence and associated factors in medical students in integrated modular curriculum: A cross-sectional study. Pakistan Journal of Medical Sciences. 2022;38:801.

86 Ishaq M, Riaz S, Iqbal N, Siddiqui S, Moin A, Sajjad S, et al. Prevalence of restless legs syndrome among medical students of Karachi: an experience from a developing country. Sleep Disorders. 2020;2020.

87 Jahangeer SMA, Hasnain N, Tariq MT, Jamil A, Zia SY, Amir W. Frequency and Association of Stress Levels with Modes of Commuting Among Medical Students of a Developing Country. The Malaysian Journal of Medical Sciences: MJMS. 2021;28:113.

88 Javaid R, Momina A, Sarwar MZ, Naqi SA. Quality of Sleep and Academic Performance among Medical University Students. Medical Education. 2020.

89 Javed M, Qureshi MA, Latif MZ. ASSESSMENT OF QUALITY OF SLEEP IN MEDICAL STUDENTS BY USING PITTSBURGH SLEEP QUALITY INDEX. Pakistan Postgraduate Medical Journal. 2023;34:12-7.

90 Joudeh RM, Jarrar RaF, Alnaser AR, Battah A, Hindi M, Battah AA, et al. Illicit drug use among medical students and its association with gender, psychological distress sleep quality and exposure to psychiatry: a nationwide study. Mental Health Review Journal. 2024;29:64-78.

91 Kazim M, Abrar A. SLEEP PATTERNS AND ACADEMIC PERFORMANCE IN STUDENTS OF A MEDICAL COLLEGE IN PAKISTAN. KUST Medical Journal. 2011;3.

92 Khan UA, Pasha SN, Khokhar SK, Rizvi AA. Sleep habits and their consequences: a survey. Rawal Med J. 2004;29:3-7.

93 Khan K, Waqas M, Sarwar R, Ahmad S, Faizan M. Effects of insomnia on daily performance of medical students: a cross sectional study conducted in university of Lahore, Pakistan. Rawal Medical Journal. 2019;44:622-.

94 Hassan S, Saqib W, Nazami A, Ghafoor K, Khan AN, Akhtar L. Association Between Sleep Patterns and Academic Performance among Medical Students. Pakistan Journal of Educational Research and Evaluation (PJERE). 2024;11.

95 Khero M, Fatima M, Shah MAA, Tahir A, Siddiqui A. Comparison of the status of sleep quality in basic and clinical medical students. Cureus. 2019;11.

96 Maalej M, Guirat M, Mejdoub Y, Omri S, Feki R, Zouari L, et al., editors. Quality of sleep, anxiety and depression among medical students during exams period: a cross sectional study. JOURNAL OF SLEEP RESEARCH; 2018: WILEY 111 RIVER ST, HOBOKEN 07030-5774, NJ USA.

97 Maheshwari G, Shaukat F. Impact of poor sleep quality on the academic performance of medical students. Cureus. 2019;11.

98 Mahfouz MS, Ageely H, Al-Saruri S, Aref L, Hejje N, Al-Attas S, et al. Sleep quality among students of the faculty of medicine in Jazan University, Saudi Arabia. Middle-East Journal of Scientific Research. 2013;16:508-13.

99 Malik N, Amama A, Shabbir A, Iqbal N, Tauseef H, Zia S, et al. Addressing Environmental Factors for SDG 3-Health and Wellbeing: Perceived Stress, Sleep Quality, and Coping among Medical Students in Pakistan. THE ASIAN BULLETIN OF GREEN MANAGEMENT AND CIRCULAR ECONOMY. 2024;4:4 (1)-89.

100 Mansour TMA, Yousef M. Nightmares among young medical students. Biomedical Research. 2016;27:437-41.

101 Mansour AE, Almokhlef S, Alqifari R, Alduwayrij M. Lifestyle diseases and associated risk behaviours among medical students in Saudi Arabia. Middle East J Fam Med. 2020;7:30.

102 Margolis SA, Reed RL. Effect of religious practices of Ramadan on sleep and perceived sleepiness of medical students. Teaching and learning in medicine. 2004;16:145-9.

103 Meo SA, Alkhalifah JM, Alshammari NF, Alnufaie WS, Algoblan AF. Impact of COVID-19 pandemic on sleep quality among medical and general science students: King Saud University Experience. Pakistan Journal of Medical Sciences. 2022;38:639.

104 Mirghani HO, Ahmed MA, Elbadawi AS. Daytime sleepiness and chronic sleep deprivation effects on academic performance among the Sudanese medical students. Journal of Taibah University Medical Sciences. 2015;10:467-70.

105 Mirghani HO, Mohammed OS, Almurtadha YM, Ahmed MS. Good sleep quality is associated with better academic performance among Sudanese medical students. BMC research notes. 2015;8:1-5.

106 Mirza AA, Baarimah H, Baig M, Mirza AA, Halawani MA, Beyari GM, et al. Academic and non-academic life stressors and their impact on psychological wellbeing of medical students. AIMS Public Health. 2021;8:563.

107 Moalla M, Maalej M, Nada C, Sellami R, Thabet JB, Zouari L. Sleep disorders, depression and anxiety among medicine university students in Sfax. European Psychiatry. 2016;33:s268-s9.

108 Mohamed RA, Moustafa HA. Relationship between smartphone addiction and sleep quality among faculty of medicine students Suez Canal University, Egypt. The Egyptian Family Medicine Journal. 2021;5:105-15.

109 Mohamed T, Masaudi E, Kambal M, Alsubaii A, Sharahili A, Alharbi A, et al. Relationship Between Sleep Duration and Academic Performance Among Saudi Medical Students at"KSAU-HS"and KSU Riyadh, Saudi Arabia. International Journal of Medicine in Developing Countries. 2024;8:066-.

110 Mohammed YAB. PREVALENCE OF SLEEP DEPRIVATION AMONG MEDICAL STUDENTS IN THE UNIVERSITY OF SCIENCE AND TECHNOLOGY, KHARTOUM. 2020.

111 Nadeem A, Cheema MK, Naseer M, Javed H. Comparison of quality of sleep between medical and non-medical undergraduate Pakistani students. J Pak Med Assoc. 2018;68:1465-70.

112 Naeem A, Kiblawi MA, Ahmad E, Samad SBA, Samad AA, Naeem A, et al. Comparison of daytime sleepiness in medical university students using epworth sleepiness scale (ESS). Nursing. 2014;7:24.

113 Naeem A, Khan U, Ali A. EFFECT OF EXCESSIVE MOBILE PHONE USAGE (HOURS) ON SLEEP PATTERNS AMONG THE MEDICAL STUDENTS OF KING EDWARD MEDICAL UNIVERSITY LAHORE. INDO AMERICAN JOURNAL OF PHARMACEUTICAL SCIENCES. 2018;5:6400-5.

114 Naseer W, Gul O, Saeed H, Qizilbash FH, Jawed Q, Mohsin SF, et al. Assessment and comparison of sleep patterns among medical and non-medical undergraduates of Karachi: A cross-sectional study-SPECIAL REPORT. JPMA The Journal of the Pakistan Medical Association. 2019;69:917-21.

115 Naveed T. Assessment of Sleep Quality among Medical Students During the Covid-19 Pandemic. Pakistan Journal of Medical & Health Sciences. 2023;17:253-.

116 Nisar M, Mohammad RM, Arshad A, Hashmi I, Yousuf SM, Baig S, et al. Influence of dietary intake on sleeping patterns of medical students. Cureus. 2019;11.

117 Qaiser DH, Albanyan OA. Sleeping disturbances/disorders in medical students of king Saud bin Abdulaziz University for Health Sciences, Riyadh. Journal of Pioneering Medical Sciences. 2018;8:9.

118 Qanash S, Al-Husayni F, Falata H, Halawani O, Jahra E, Murshed B, et al. Effect of Electronic Device Addiction on Sleep Quality and Academic Performance Among Health Care Students: Cross-sectional Study. JMIR medical education. 2021;7:e25662.

119 Quronfulah BS, Aboalshamat KT, Badri HM, Mahmoud MA, Rajeh MT, Badawoud AM, et al. The Effect of Sleep Quality on Psychological Distress Among Saudi Healthcare Students and Professionals. International Journal of Pharmaceutical Research & Allied Sciences. 2023;12.

120 Rafeeq S, Naman MS, Ijaz MT, Touseef M, Rai IA, Chaudhry MA. Association of excessive smartphone use with sleep in students of a private medical college in Lahore, Pakistan. Rawal Med J. 2021;46:947-50.

121 Rafiq CA, Ali AA, Ahmad T. Effect of Sleep Disturbance on Academic Performance of Students of Public Sector Medical College of Pakistan. INTERNATIONAL JOURNAL OF ADVANCED BIOTECHNOLOGY AND RESEARCH. 2017;8:546-+.

122 Rejeb H, Kaddoussi R, Saida IB, Khelifa MB, Najjar A, Aissa S, et al. Sleep patterns and predictors of poor sleep quality among Tunisian medical students (MS). Eur Respiratory Soc; 2018.

123 Safhi MA, Alafif RA, Alamoudi NM, Alamoudi MM, Alghamdi WA, Albishri SF, et al. The association of stress with sleep quality among medical students at King Abdulaziz University. Journal of Family Medicine and Primary Care. 2020;9:1662.

124 Saguem B, Nakhli J, Romdhane I, Nasr S. Predictors of sleep quality in medical students during COVID-19 confinement. L'encephale. 2022;48:3-12.

125 Saif M, Ahmed H, Shabbeer F, Ilyas H. Unlocking the secrets of success: learning and relaxing techniques among high achiever medical students attending a private medical school in Lahore. Sleep. 2024;11:19.

126 Ahmed Salama A. Sleep Quality in Medical Students, Menoufia University, Egypt. The Egyptian Family Medicine Journal. 2017;1:1-21.

127 Sameer HM, Imran N, Tarar TN. EXCESSIVE DAYTIME SLEEPINESS AND ITS RELATION WITH QUALITY OF LIFE AND ACADEMIC PERFORMANCE IN MEDICAL STUDENTS. Khyber Medical University Journal. 2020;12:299-304.

128 Satti MZ, Khan TM, Azhar MJ, Javed H, Yaseen M, Raja MT, et al. Association of physical activity and sleep quality with academic performance among fourth-year MBBS students of Rawalpindi Medical University. Cureus. 2019;11.

129 Zarafshan Shafique, Faiza Syed, Safia Naz, Saba Urooj, Sadia Khan, Javed. S. Assessment of factors affecting the sleep hygiene of medical students in Bahawalpur, Pakistan: a cross-sectional study. 2021.

130 Shehata Farag Shehata, Mohammed Saad Alshahrani, Mohammed Saad Aldarami, Faisal Ali Asiri, Alghamdi HA. Prevalence and association between sleep, stress, and physical activity among medical students in southern region, Saudi Arabia. World family medicine. 2020:93-101.

131 Shehata YA, Sharfeldin AY, El Sheikh GM. Sleep Quality as a Predictor for Academic Performance in Menoufia University Medical Students. The Egyptian Journal of Hospital Medicine. 2022;89:5101-5.

132 Shukri AK. Correlation between Demographic and Socio-Economic Factors among Undergraduate Medical Students in Taif University, Saudi Arabia. International Journal of Pharmaceutical Research & Allied Sciences. 2019;8.

133 Siddiqui AF, Al-Musa H, Al-Amri H, Al-Qahtani A, Al-Shahrani M, Al-Qahtani M. Sleep patterns and predictors of poor sleep quality among medical students in King Khalid University, Saudi Arabia. The Malaysian journal of medical sciences: MJMS. 2016;23:94.
134 Surani AA, Zahid S, Surani A, Ali S, Mubeen M, Khan RH. Sleep quality among medical students of Karachi, Pakistan. J Pak Med Assoc. 2015;65:380-2.
135 Al Suwayri SM. The impact of social media volume and addiction on medical student sleep quality and academic performance: a cross-sectional observational study. Imam Journal of Applied Sciences. 2016;1:80.
136 Swed S, Bohsas H, Alibrahim H, Rais MA, Elsayed M, Nashwan AJ, et al. Internet Addiction and Depression among Syrian College Students: A Cross-Sectional Study. 2023.
137 Tahir M, Haiy AU, Tahir M, Kuraishi RT, Saqib S. Sleeping Habits among Medical Students of King Edward Medical University, Associated Stress and Effects on Academic Performance. Annals of King Edward Medical University. 2020;26:379-83.
138 Talih F, Daher M, Daou D, Ajaltouni J. Examining burnout, depression, and attitudes regarding drug use among Lebanese medical students during the 4 years of medical school. Academic Psychiatry. 2018;42:288-96.
139 Tauseef H, Siddque H, Akhtar M, Hurera A. Frequency of irritable bowel syndrome (IBS) and its risk factors among MBBS students of Allama Iqbal Medical College, Lahore. Pak J Med Health Sci. 2021;15:2062-7.
140 Thobani H, Fatima SS. The Prevalence and Risk Factors For Poor Sleep Quality Amongst Medical Students: A Cross Sectional Study Funding. BioSight. 2023;4:24-32.
141 Toubasi A, Khraisat B, AbuAnzeh R, Kalbouneh H. A cross sectional study: The association between sleeping quality and stress among second and third medical students at the University of Jordan. The International Journal of Psychiatry in Medicine. 2022;57:134-52.
142 Traore B, Aguilo Y, Hassoune S, Nani S. Determinants of internet addiction among medical students in Casablanca: a cross-sectional study. Global Health Journal. 2023.
143 Usman G, Abbas K, Zaheer M, Kaghazwala T, Mushtaque A, Alamgir M. Patterns of sleep and the metabolic and neurobehavioral effects of sleep deprivation reported by medical students. Ann Jinnah Sindh Med Uni. 2017;3:75-9.
144 Varanasi S, Tom C, Syed M, Sultana SS, Goud B, Kumar JB. Healthy sleep keeps troubles away: A study on the effects of sleep deprivation on stress in undergraduate students of RAKMHSU. Indian Journal of Basic and Applied Medical Research. 2020;10:65-72.
145 Dr. Mohammed Vohra BA, Waleed AlTulaiqi, Turki AlOtaibi, Mohammed AlShehri, Ali AlQahtani, Abdulaziz AlBulaihed , Abdullah AlKhamri THE ASSOCIATION BETWEEN CAFFEINE CONSUMPTION AND SLEEPING HABITS AMONG MEDICAL STUDENTS. INDO AMERICAN JOURNAL OF PHARMACEUTICAL SCIENCES. 2019;06(03):4913-21.
146 Waqas A, Khan S, Sharif W, Khalid U, Ali A. Association of academic stress with sleeping difficulties in medical students of a Pakistani medical school: a cross sectional survey. PeerJ. 2015;3:e840.
147 Yassin A, Al-Mistarehi A-H, Yonis OB, Aleshawi AJ, Momany SM, Khassawneh BY. Prevalence of sleep disorders among medical students and their association with poor academic performance: A cross-sectional study. Annals of medicine and Surgery. 2020;58:124-9.
148 Zafar M, Omer EO, Elfatih M, Ansari K, Kareem A, Fares R, et al. Daytime sleepiness and sleep quality among undergraduate medical students in Dammam, Saudi Arabia. Indian Journal of Medical Specialities. 2020;11:148.
149 Zafar M, Omer EO, Hassan ME, Ansari K. Association of sleep disorder with academic performance among medical students in Sudan. Russian Open Medical Journal. 2020;9:208.
150 Zainab S, Soomro RA, Khoso A, Qazi NA. Frequency and Predictors of Sleep Disorders in Undergraduate Medical Students. Journal of Liaquat University of Medical & Health Sciences. 2020;19:109-15.
151 World Bank Group country classifications by income level. 2023. Available: https://datatopics.worldbank.org/world-development-indicators/the-world-by-income-and-region.html. Accessed.
152 Hoy D, Brooks P, Woolf A, Blyth F, March L, Bain C, et al. Assessing risk of bias in prevalence studies: modification of an existing tool and evidence of interrater agreement. Journal of Clinical Epidemiology. 2012;65:934-9.
